# Xenoacremones D–H, Bioactive Tyrosine-decahydrofluorene Analogues from the Plant-Derived Fungus *Xenoacremonium sinensis*

**DOI:** 10.3390/md20060375

**Published:** 2022-05-31

**Authors:** Zhiguo Liu, Li Liu, Anqi Wang, Li Li, Sinan Zhao, Yanan Wang, Yi Sun

**Affiliations:** 1 Institute of Chinese Materia Medica, China Academy of Chinese Medical Sciences, Beijing 100700, China; liuzhiguo1321@163.com (Z.L.); lliu1990@icmm.ac.cn (L.L.); mickey-wang@139.com (A.W.); 18530869018@163.com (S.Z.); 2 Institute of Materia Medica, Chinese Academy of Medical Sciences & Peking Union Medical College, Beijing 100050, China; annaleelin@imm.ac.cn (L.L.); wangyanan@imm.ac.cn (Y.W.)

**Keywords:** tyrosine-decahydrofluorene, endophytic fungus, *Xenoacremonium sinensis*, anti-inflammatory, cytotoxic activity

## Abstract

Five novel tyrosine-decahydrofluorene analogues, xenoacremones D–H (**1–5**), each bearing a fused 6/5/6 tricarbocyclic core and a 13-membered *para*-cyclophane ring system, were isolated from the endophytic fungus *Xenoacremonium sinensis*. Compound **1** was a novel polyketide synthase–nonribosomal peptide synthetase (PKS–NRPS) tyrosine-decahydrofluorene hybrid containing a 6/5/6/6/5 ring system. Their structures were elucidated from comprehensive spectroscopic analysis and electronic circular dichroism (ECD) calculations. All compounds were evaluated for their inhibitory activities on LPS-induced NO production in macrophages and their cytotoxicities against the NB4 and U937 cell lines. Compounds **3** and **5** exhibited potent anti-inflammatory activities in vitro. Compounds **1** and **3**–**5** displayed significant antiproliferative activity against the tumor cell lines (IC_50_ < 20 µM).

## 1. Introduction

**Figure 1:**
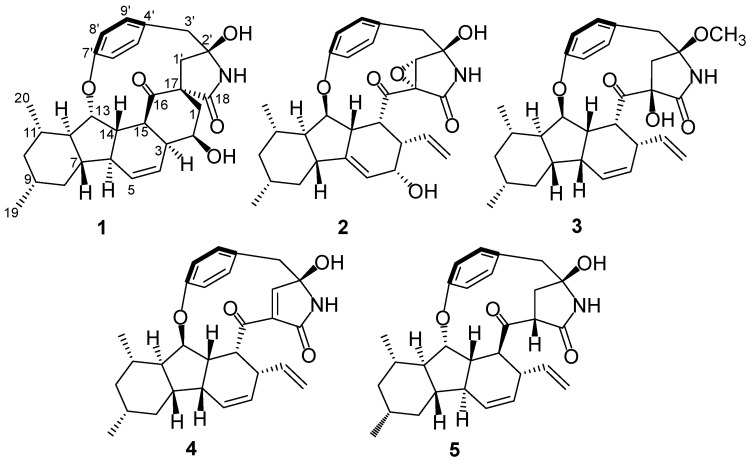
Structures of compounds **1**–**5.**

Endophytic fungi from medicinal plants are known for their ability to produce a variety of bioactive natural products with novel skeletons [1,2,3,4]. Their secondary metabolites, such as alkaloids, terpenoids and polyketides, possess antibacterial, cytotoxic, anti-inflammatory, antidiabetic and other biological activities [5,6,7]. The genus *Xenoacremonium* belongs to the family Nectriaceae, which is rarely reported [8]. *X. sinensis* was first isolated by our team and identified as a new species in 2019 (Figure S1). Filamentous fungi such as Nectriaceae can produce a variety of polyketide–nonribosomal peptide (PK–NRP) hybrids. The members of the unusual family of tyrosine-decahydrofluorenes are rare polyketide synthase–nonribosomal peptide synthetase (PKS-NRPS) secondary metabolites in nature; they include hirsutellones A–F [9,10], pyrrocidines A, B and their analogues [11,12,13], GKK1032A_2_ [14], penicipyrrodiether [15] and trichogamide A [16]. These PKS-NRPS hybrids were isolated from various fungal species, some of which were from the family Nectriaceae, such as *Hirsutella* spp. [9,17], *Neonectria ramulariae* [12,18] and *Acremonium zeae* [19]. They share a structural skeleton that contains a fused [6,5,6] tricarbocyclic decahydrofluorene, a γ-lactam and a 13-membered *para*-cyclophane ether ring system, and they possess antitumor, antifungal, antibacterial and antitubercular activities [20]. The unique PKS-NRPS skeletons have attracted considerable attention from chemists, and hirsutellones have been successively synthesized using different methods [21,22,23,24,25]. In our previous research on antitumor secondary metabolites from endophytic fungi, we obtained three novel tyrosine-decahydrofluorene derivatives, xenoacremones A–C, from *X. sinensis*, isolated from twigs of the mangrove plant *Ceriops tagal*. The biosynthetic pathway of these compounds was clarified by gene deletion in *X. sinensis* and heterologous expression investigation [26]. Subsequent studies on the bioactive analogues in *X. sinensis* (ML-31) have obtained five new tyrosine-decahydrofluorene derivatives, xenoacremones D–H (**1**–**5**) (Figure 1). Herein, we describe the isolation, structural elucidation and biological activities of compounds **1**–**5**.

## 2. Results and Discussion

### 2.1. Structure Elucidation

Xenoacremone D (**1**) was obtained as a white solid, and its molecular formula was determined to be C_29_H_35_NO_5_ by high-resolution electrospray ionization mass spectrometry (HRESIMS) data (Figure 1), requiring 13 degrees of unsaturation. The ^1^H NMR data of **1** (Table 1) revealed a 1,4-substituted benzene ring with axial chirality (*δ*_H_ 6.96, 6.95, 6.94 and 6.70), two olefinic protons (*δ*_H_ 6.02 and 5.60), two oxygenated methines (*δ*_H_ 5.16 and 4.75) and two doublet methyls (*δ*_H_ 1.11 and 0.96). The ^13^C NMR and HSQC spectra (Table 1) showed one ketone and one amide carbonyl group, ten quaternary carbons, ten methines, five methylenes and two methyls. 

**Figure 2:**
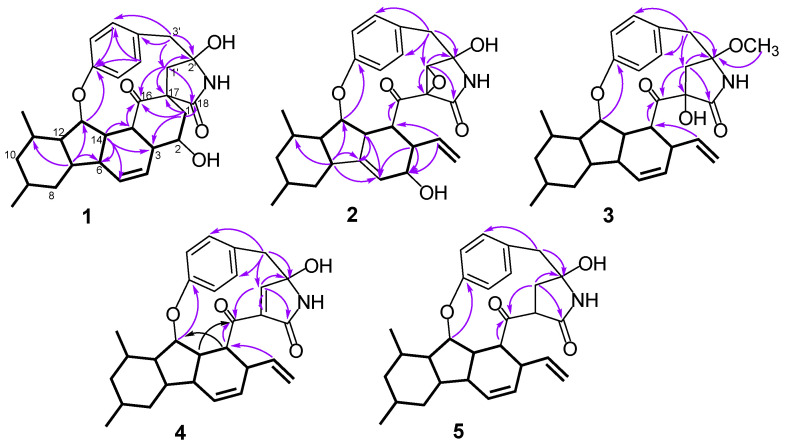
Key HMBC and ^1^H-^1^H COSY correlations of compounds **1**–**5**.

Analysis of its 2D NMR data confirmed the whole structure, which had a tyrosine-decahydrofluorene skeleton and resembled that of hirsutellone B and pyrrospirone A [9,13]. The proton spin systems from H-1 to H-15 observed in the ^1^H-^1^H COSY spectrum, as well as the HMBC correlations from H-7 to C-6, C-11 and C-13 and from H-14 to C-3, C-5, C-6 and C-15, indicated the presence of a decahydrofluorene moiety. Furthermore, the ^1^H-^1^H COSY cross-peaks of H-1/H-2/H-3 and the HMBC correlations from H-1 to C-3, C-16, C-17 and C-18 and from H-1’ to C-1, C-16 and C-17 revealed the presence of a methylene (C-1), an oxygenated methine (C-2) and a quaternary carbon (C-17) in **1**, which were different from those of hirsutellone B (Figure 2). The HMBC correlations and the degrees of unsaturation indicated that the methylene at C-1 was linked at C-17 to form a cyclohexane moiety, and C-17 was the connectivity of a spiro center between the cyclohexane and *γ*-lactam ring. Additional HMBC correlations from H-1’ and H-3’ to a quaternary carbon C-2’ (*δ*_C_ 83.5) led to the assignment of C-2’ for the γ-position of the lactam ring, and its up-field shift revealed the attachment of a hydroxyl group. In addition, the HMBC correlations from H-1’ to C-3’, C-16 and C-18 and from H-3’ to C-5’ and C-9’ completed the linkages of the phenyl and 6/5/6/6/5 pentacarbocyclic moieties to form the 13-membered macrocyclic ether of **1**. Consequently, its planar structure containing a spiro-ring system was determined. 

**Figure 3:**
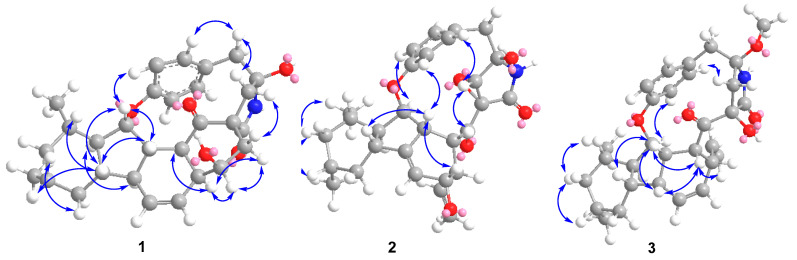
Key NOE correlations of compounds **1**–**3**.

**Figure 4:**
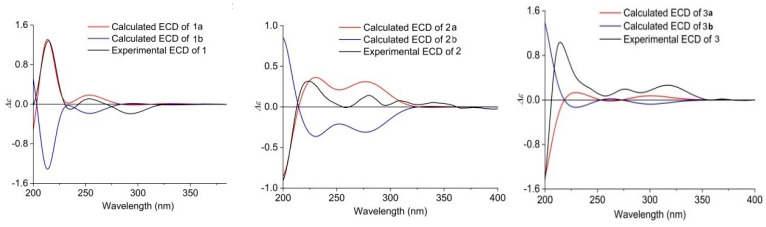
Experimental and calculated ECD of compounds **1**–**3** (in MeOH).

The relative configuration of **1** was ascertained by the NOESY experiment. The NOE cross-peaks of H-7/H-11, H-7/H-9, H-12/H-8a and H-12/H-10a indicated these protons were axial positions. Additional NOE cross-peaks of H-7/H-14, and H-13/H-6’ placed these protons in the β-orientation (Figure 3). However, the NOE cross-peaks of H-6/H-12 and H-15/H-3 indicated that they were co-facial. Further NOE cross-peaks of H-1/H-1’β and H-1α/H-3 revealed that the methylene at C-1 was β-oriented and C-18 of the *γ*-lactam was α-oriented. The vicinal coupling constant (*J*_12,13_ = 8.3 Hz) between H-12 and H-13 implied their trans-configuration, and H-13 was axial. Moreover, the Δ^4,5^ geometry was assigned as *Z* by its coupling constant (*J* = 9.1 Hz). To determine the absolute stereochemistry of **1,** the theoretically calculated electronic circular dichroism (ECD) spectra were obtained by time-dependent density functional theory (TDDFT). The conformations of **1a** and **1b** (**1b** was the enantiomer of **1a**) were compared using ECD calculations at the B3LYP level. The Cotton effects were identical with the calculated curve of the enantiomer **1a** (Figure 4), which confirmed its absolute configuration as 2*R*,3*S*,6*S*,7*S*,9*R*,11*S*,12*R*,13*S*,14*S*,15*S*,17*S*,2’*R*.

Xenoacremone E (**2**) was shown via HRESIMS to have a molecular formula of C_29_H_33_NO_6_. The ^1^H and ^13^C NMR data (Table 1) of **2** were also similar to those of hirsutellone B. The position of the double bond in **2** was deduced by the ^1^H-^1^H COSY cross-peaks between H-3 and H-5 and key HMBC correlations from H-7, H-14 and H-5 to C-6 and from H-7, H-14 and H-3 to C-5 (Figure 2). Further HMBC correlations from H-3 to C-4 and C-5 and from H-2 to C-4, as well as the chemical shifts of H-4 (*δ*_H_ 3.84, dd, *J* = 4.2, 1.2 Hz) and C-4 (*δ*_C_ 67.3), indicated the presence of a hydroxyl group at C-4. Additional HMBC correlations from H-3’ to C-1’ (*δ*_C_ 63.5) and from H-1’ (*δ*_H_ 3.6, s) to the quaternary carbons C-17 (*δ*_C_ 58.7), C-18 and C-2’, combined with the degree of unsaturation of **2**, indicated the presence of an epoxide moiety at C-1’ and C-17 in the *γ*-lactam ring. The NOE correlations (Figure 3) of H-14 to H-3/H-7, H-15 to H-1’ and H-1’ to H-9’ indicated that these hydrogens were co-facial and β-oriented, while the correlations of H-13 to H-6’, H-10α to H_3_-19/H_3_-20 hinted that these hydrogens were α-oriented. The ECD spectrum of **2** was determined and the Cotton effects were identical with the calculated curve of the enantiomer **2a** (Figure 4). Thus, the absolute configuration of **2** was assigned (3*S*,4*S*,7*R*,9*R*,11*S*,12*R*,13*R*,14*R*,15*R*,17*S*,1’*S*,2’*R*) by comparison of the experimental and calculated ECD spectra (Figure 4).

Xenoacremone F (**3**) was determined to be C_30_H_37_NO_5_ on the basis of its HR ESIMS and NMR spectra (Table 1). Comparison of its NMR data with those of **2** revealed that **3** had one more methoxyl group (*δ*_C_ 49.7, *δ*_H_ 3.24) and one less epoxide group than **2**. The HMBC correlations from –OCH_3_ to C-2’ confirmed the assignment of the methoxyl group at C-2’ (Figure 2). Further HMBC correlations from H-1’ to C-17, C-18 and C-2’ indicated that the hydroxyl group was located at C-17 in the γ-lactam ring. The NOE correlations (Figure S3) of H-6 to H-7/H-14, H-7 to H-9/H-11/H-14, H-15 to H-1’ and H-3’β to H-9’ indicated that these hydrogens were in the β-orientation, similar to those of **2**. The NOE correlations of H-13 to H-6’, H-9’ to H-1’α and H-3’α, as well as H-10α to H_3_-19/H_3_-20/H-12 indicated their α-orientation. The ECD spectrum was determined to elucidate the absolute configuration, which was compared to the experimental ECD curve. The ECD spectrum of **3** generated for the tyrosine-decahydrofluorene rings resembled that of compound **2**, which was consistent with the experimental data of **3a** (Figure S4). Therefore, the absolute configuration of **3** was established as depicted in Figure 1.

Xenoacremone G (**4**) has the molecular formula C_29_H_33_NO_4_ with 14 degrees of unsaturation, as determined by HR ESIMS data. The ^1^H and ^13^C NMR spectroscopic data of **4** (Table 1) were similar to those of **3**, and the differences were the absence of the methoxyl group at C-2’ and the presence of an extra double bond (*δ*_C_ 153.7, *δ*_H_ 6.44; *δ*_C_ 134.8) in **4**. Further analysis of the 2D NMR data, particularly the HMBC correlations (Figure 2) from H-1’ to C-17 and C-2’ and from H-3’ to C-1’, confirmed the location of the double bond at C-1’ and C-17. The relative configuration of **4** was assigned as depicted by key NOE correlations (Figure S3) of H-6 to H-14, H-14 to H-3, H-3’β to H-9’, H-13 to H-6’ and H-15 to H-1’. The absolute configuration of **4** was identified by comparing its experimental and calculated ECD data (Figure S3). Therefore, **4** was confirmed as 3*R*,6*R*,7*S*,9*R*,11*S*,12*R*,13*R*,14*S*,15*R*,2’*R*, and it had ECD effects similar to those of **2** (Figure S4).

The HR ESIMS data of xenoacremone H (**5**) suggested that it had the molecular formula of C_29_H_35_NO_4_. Comprehensive analysis of NMR data for the two compounds indicated that **5** possessed the similar planar structure as **4**, where a pair of double bond in *γ*-lactam ring disappeared (Table 1). The NOESY correlations (Figure S3) of H-12 to H-6 and H-15 to H-6 placed these hydrogens in the *α*-orientation, while the correlations of H-7 to H-14, H-13 to H-11/H-8’ and H-3 to H-14/H-17 indicated that these hydrogens were in the β-orientation, indicating that **5** had a similar stereochemistry than **1**. The vicinal coupling constant (*J*_12,13_ = 7.7 Hz) between H-12 and H-13 implied their trans-configuration, which was consistent with that of compound **1**. The absolute configuration of **5** was determined by ECD calculation (Figure S4). Its ECD curve was similar to that of **1** (Figure 4). The experimental ECD spectrum of **5** was in accordance with the calculated ECD spectrum for **5a**. Therefore, the absolute configuration of **5** was established as 3*R*,6*S*,7*S*,9*R*,11*S*,12*R*,13*S*,14*S*,15*S*,17*S*,2’*S*.

### 2.2. Biological Assay

Compounds **1–5** were evaluated for their anti-inflammatory activities based on the inhibition of nitric oxide (NO) production in LPS-induced RAW264.7 macrophages. The results showed that compounds **3** and **5** exhibited significant inhibitory activities against the production of NO, with IC_50_ values in the vicinity of 12.8 and 6.7 μM, respectively (Table 2). Compound **5** showed the most potent inhibitory activity, which was stronger than the positive control resveratrol and compounds such as caffeic acid phenthyl ester (9.3 μM) and aspirin (43.2 μM). All the compounds were also examined for their cytotoxicity against the NB4 and U937 cell lines. Compounds **1** and **3**–**5** displayed cytotoxicities with IC_50_ values less than 20 µM (Table 3). 

## 3. Materials and Methods

### 3.1. General Experimental Procedures

NMR spectra were measured on a Bruker ARX-600 spectrometer (600 MHz, Bruker Co., Ltd., Karlsruhe, Germany). The ^1^H and ^13^C NMR chemical shifts of **1–4** were recorded relative to the solvent peaks of CD_3_OD (*δ*_H_ 3.31 and *δ*_C_ 49.00), and the ^1^H and ^13^C NMR chemical shifts of **5** were recorded relative to the solvent peaks of CDCl_3_ (*δ*_H_ 7.26 and *δ*_C_ 77.16). Optical rotations were recorded with a PerkinElmer 241 polarimeter. ECD spectra were obtained on a JASCO J-815 spectrometer (Tokyo, Japan). HRESIMS data were recorded on a Waters Vion QTOF/MS spectrometer (Waters Micromass, Manchester, UK) in positive electrospray ionization mode. High-performance liquid chromatography (HPLC) was carried out on an Agilent 1260 quaternary system with a UV detector (Agilent, Technologies Co., Ltd., Palo Alto, CA, USA) combined with analytical, semipreparative or preparative Cosmosil C18-MSII columns (250 mm × 4.6 mm or 250 mm × 10 mm). A UPLC reversed-phase C18 analytical column (35 °C, 2.1 mm × 100 mm, 1.7 μm, BEH, Waters) was adopted. Thin-layer chromatography (TLC) was performed on a silica gel plate GF254 (Qingdao Haiyang Chemical Co., Ltd., Qingdao, China). Column chromatography (CC) was performed on Sephadex LH-20 (Pharmacia Fine Chemical Co., Ltd., Uppsala, Sweden), ODS (50 μm, YMC, Tokyo, Japan) and silica gel (200–300 mesh, Qingdao Haiyang Chemical Ltd., Qingdao, China) columns. Human carcinoma cell lines U937, NB4 and A549 were obtained from the Chinese National Infrastructure of Cell Line Resource (NICR). PBS was purchased from HyClone (Solarbio, Beijing, China). FBS was purchased from Every Green (TIANHANG, China). CCK-8 was purchased from Dojindo (Beijing, China).

### 3.2. Fungal Material

The endophytic fungus ML-31 was isolated from twigs of the mangrove plant *Ceriops tagal* collected in Hainan Province, China, in July 2013. The plant species was identified by Yi Sun, and the fungus was identified as *X. sinensis* on the basis of its rRNA gene sequence. The accession number for the biosynthetic cluster (*xen*) is MT876600 in the GenBank database at NCBI. The strain was deposited at the Institute of Chinese Materia Medica, China Academy of Chinese Medical Sciences.

### 3.3. Extraction and Isolation

The fungus ML-31 was cultured on rice medium for 7 days. Then, fermented rice substrate was cut into small fragments, which were ultrasonically extracted with EtOAc three times. The solvent was then removed under reduced pressure (under vacuum) to yield the total extract (5.3 g). The crude extract was fractionated by ODS flash CC (5 × 30 cm) and eluted with 2 L each of MeOH-H_2_O (20:80, 40:60, 60:40, 80:20, 100:0). The fraction eluted with 80% MeOH was subjected to Sephadex LH-20 chromatography (CH_2_Cl_2_:MeOH 1:1) to obtain six subfractions (A–F), and subfraction B was subsequently purified by HPLC (Kromasil Eternity XT-5-C18 column, 250 mm × 10 mm i.d., 5 μm, 2 mL min^−1^), with gradient elution from 70% to 80% MeOH in H_2_O with 0.2% AcOH to afford compounds **1** (*t*_R_ = 15.0 min, 3.2 mg) and **5** (*t*_R_ = 27.2 min, 2.5 mg). The fraction eluted with MeOH-H_2_O (60:40) was subjected to Sephadex LH-20 chromatography (CH_2_Cl_2_-MeOH, 1:1) to yield five subfractions (A–E). Fraction C was then isolated by silica gel CC (200−300 mesh) and eluted with a CH_2_Cl_2_-acetone gradient system (50:1, 20:1, 10:1, 6:1, 3:1, 2:1, 1:1) to yield five subfractions. The five fractions were analyzed by HPLC. Fraction 4.1 was further purified by HPLC (ACN/H_2_O, 45%/55%) to obtain compounds **2** (2.4 mg) and **3** (3.6 mg), and fraction 4.3 was subjected to HPLC with 55% MeCN in H_2_O to afford compound **4** (4.5 mg).

Compound **1**: white solid; [α]25D –16.7 (*c* 0.10, MeOH); CD (MeOH) 215 (Δ*ε* +8.87) nm, 235 (Δ*ε* −0.70) nm, 254 (Δ*ε* +0.77) nm, 293 (Δ*ε* −1.32) nm. ^1^H NMR (600 MHz, CD_3_OD) and ^13^C NMR (150 MHz, CD_3_OD) data (Table 1). HR-ESIMS *m*/*z* 478.2606 [M + H]^+^, calcd for C_2__9_H_3__6_NO_5_, 478.2588.

Compound **2**: white solid; [α]25D +25.7 (*c* 0.10, MeOH); CD (MeOH) 224 (Δ*ε* +0.32) nm, 278 (Δ*ε* −0.16) nm, 315 (Δ*ε* +0.07) nm. ^1^H NMR (600 MHz, CD_3_OD) and ^13^C NMR (150 MHz, CD_3_OD) data (Table 1). HR-ESIMS *m*/*z* 492.2395 [M + H]^+^, calcd for C_2__9_H_34_NO_6_, 492.2381.

Compound **3**: white solid; [α]25D +3.8 (*c* 0.10, MeOH); CD (MeOH) 215 (Δ*ε* +1.04) nm, 276 (Δ*ε* +0.19) nm, 318 (Δ*ε* +0.26) nm. ^1^H NMR (600 MHz, CD_3_OD) and ^13^C NMR (150 MHz, CD_3_OD) data (Table 1). HR-ESIMS *m*/*z* 492.2766 [M + H]^+^, calcd for C_30_H_38_NO_5_, 492.2745.

Compound **4**: white solid; [α]25D +11.2 (*c* 0.10, MeOH); CD (MeOH) 222 (Δ*ε* +3.25) nm, 285 (Δ*ε* +1.15) nm, 331 (Δ*ε* +0.80) nm. ^1^H NMR (600 MHz, CD_3_OD) and ^13^C NMR (150 MHz, CD_3_OD) data (Table 1). HR-ESIMS *m*/*z* 460.2486 [M + H]^+^, calcd for C_29_H_34_NO_4_, 460.2482.

Compound **5**: white solid; [α]25D −7.2 (*c* 0.10, MeOH); CD (MeOH) 214 (Δ*ε* +8.72) nm, 237 (Δ*ε* −0.51) nm, 255 (Δ*ε* +0.29) nm, 300 (Δ*ε* −1.18) nm. ^1^H NMR (600 MHz, CD_3_OD) and ^13^C NMR (150 MHz, CD_3_OD) data (Table 1). HR-ESIMS *m*/*z* 462.2443 [M + H]^+^, calcd for C_29_H_36_NO_4_, 462.2439.

### 3.4. Computational of ECD

A conformational search was carried out in the MMFF94 molecular mechanics force field using the MOE (Molecular Operating Environment) software package [27], and all the conformers within an energy window of 10 kcal/mol were regarded as the initial conformations. Monte Carlo protocols were used in the experiment. The geometry optimization and frequency calculations were performed with Gaussian16 RevB.01 [28], using the ωB97XD or B3LYP functional at the 6-311G (d,p) level of theory to verify the stability and obtain the energies at 298.15 K and 1 atm pressure. The Boltzmann distribution was calculated according to their Gibbs free energies. ECD calculations were conducted by using the Cam-B3LYP functional at the TZVP level of theory. The solvation model based on density (SMD) was used as the solvation model [29]. The Boltzmann-averaged ECD spectra were obtained by using SpecDis 1.71 software [30]. Methanol was used for structural optimization.

### 3.5. Cytotoxicity Assays

The cytotoxicities of **1**–**5** against human carcinoma cells of lines U937 and NB4 were tested using the CCK-8 method. The cells were sustained in RPMI-1640 supplemented with 10% (*v*/*v*) fetal bovine serum (FBS) and 0.5% (*v*/*v*) penicillin–streptomycin solution (10,000 units/mL penicillin and 10,000 μg/mL streptomycin, 100×) in a humidified atmosphere containing 5% CO_2_ at 37 °C. The cells were digested by trypsinization and then diluted to a concentration of 1 × 10^4^ cells/mL. The diluted cell suspensions were then placed into 96-well microtiter plates and incubated with the test samples for 72 h. The control contained 2 μL of MeOH. After incubation, CCK-8 solution was added to each well, and the plates were incubated for 4 h. The absorption was measured at a wavelength of 450 nm.

### 3.6. Assay of the Inhibition of NO Production in RAW264.7 Murine Macrophages

The nitrite concentration in the medium was measured as an indicator of NO production according to the Griess reaction. RAW 264.7 macrophages were seeded in three replicates at a density of 1 × 10^5^ cells/well and incubated overnight at 37 °C with 5% CO_2_. The cells were then treated with the range (3.125~50 μg/mL) of compounds **1**–**5** in the presence of 1 μg/mL LPS for 30 min. The culture supernatant was aspirated, and the cells were further incubated at the same LPS concentration for 24 h. After incubation, 100 mL of cell-free supernatant was mixed with 100 μL of Griess reagent containing equal volumes of 2% (*w*/*v*) sulfanilamide in 5% (*w*/*v*) phosphoric acid and 0.2% (*w*/*v*) N-(1-naphthyl) ethylenediamine solution to determine nitrite production. Absorbance was measured in a microplate reader at 540 nm against a calibration curve with NaNO_2_ standards. Data are expressed as the mean ± SD of three independent experiments.

## 4. Conclusions

In summary, we isolated and fully identified five novel tyrosine-decahydrofluorene analogues bearing a rare fused 6/5/6 tricarbocyclic core and a 13-membered *para*-cyclophane ring system from the endophytic fungus *X. sinensis* ML-31. Compound **1** had a 6/5/6/6/5 pentacarbocyclic skeleton with a [5,6]-spiro ring and a *para*-cyclophane ring system. Compounds **1**, **3** and **5** showed significant cytotoxic activities against the NB4 and U937 cell lines (IC_50_ < 20 μM). Compounds **3** and **5** also exhibited potent inhibitory activities against the production of NO (IC_50_, 12.8 and 6.7 μM, respectively). Our findings expand the knowledge of tyrosine-decahydrofluorene derivatives and can further facilitate biosynthesis investigation.

## Figures and Tables

**Table 1:** ^1^H NMR (600 MHz) and ^13^C NMR data of compounds **1**–**5** in CD_3_OD.

	1		2		3 *^a^*		4 *^a^*		5 *^b^*	
No.	*δ*_C_	*δ*_H_ (*J* in Hz)	*δ*_C_	*δ*_H_ (*J* in Hz)	*δ*_C_	*δ*_H_ (*J* in Hz)	*δ*_C_	*δ*_H_ (*J* in Hz)	*δ*_C_	*δ*_H_ (*J* in Hz)
1	49.9	H_α_: 1.38, t (12.2) H_β_: 2.10, m	119.0	4.90, d (17.0) 4.97, d (16.8)	119.2	4.91, d (17.1) 5.09, d (10.2)	117.6	4.88, m 4.98, dd (10.2, 1.3)	116.7	5.07, d (10.6) 5.10, d (17.5)
2	64.6	5.16, dd (12.2, 6.0)	134.8	5.30, dd (16.8, 9.0)	139.0	5.77, ddd (17.1, 10.4, 5.7)	140.7	5.71, dd (17.0, 9.6)	137.9	5.80, ddd (17.5, 10.4, 7.1)
3	48.9	2.86, m	48.7	2.56, m	43.8	3.33, m	46.8	2.42, m	43.1	2.90, m
4	125.8	5.60, dd (9.1, 3.3)	68.8	3.84, dd (4.2, 1.2)	127.7	5.25, dd (9.6, 4.6)	130.5	5.65, dd (9.0, 4.2)	126.9	5.61, dd (9.0, 3.1)
5	132.8	6.02, dd (9.1, 3.0)	118.0	5.56, d (1.2)	130.6	6.01, d (9.6)	133.4	5.95, d (9.0)	133.0	6.24, dd (9.0, 2.9)
6	46.5	1.45, m	148.7		41.8	2.97, m	44.0	2.19, m	43.1	1.64, m
7	46.3	1.51, m	46.3	2.63, m	42.7	2.24, m	48.1	1.91, m	48.5	1.53, m
8	39.4	H_α_: 2.01, m H_β_: 0.67, ddd (11.7, 11.7, 5.7)	39.5	H_α_: 2.09, m H_β_: 0.89, m	38.6	H_α_: 1.97, m H_β_: 0.77, m	41.1	H_α_: 2.10, m H_β_: 0.81, m	38.7	H_α_: 2.01, m H_β_: 0.62, m
9	33.9	1.52, m	34.6	1.63, m	35.5	1.61, m	34.1	1.57, m	32.4	1.55, m
10	46.7	H_α_: 1.80, m H_β_: 0.72, ddd (11.7, 11.7, 5.7)	45.9	H_α_: 1.76, m H_β_: 0.73, m	46.5	H_α_: 1.76, m H_β_: 0.66, m	45.4	H_α_: 1.76, m H_β_: 0.74, m	45.1	H_α_: 1.78, m H_β_: 0.69, m
11	33.1	1.89, m	32.9	1.78, m	33.8	1.74, m	32.4	1.78, m	31.5	1.87, m
12	58.3	1.20, m	57.0	1.10, m	61.7	1.01, m	57.5	1.03, m	57.7	1.05, m
13	86.6	4.75, dd (8.3, 6.5)	80.4	4.98, m	82.4	5.04, m	83.2	4.65, t (3.4)	88.7	4.47, dd (7.7, 4.9)
14	51.7	2.13, m	46.8	2.64, m	47.5	1.79, m	49.8	2.70, m	54.3	1.95, m
15	45.8	3.57, dd (7.9, 6.8)	46.4	2.45, dd (11.4, 3.6)	50.4	2.68, dd (10.2, 5.4)	51.8	2.45, dd (10.8, 6.6)	49.5	3.80, t (8.0)
16	202.4		201.9		206.1		203.2		201.6	
17	60.5	-	60.8	-	81.1	-	134.8	-	56.5	3.12, dd (12.2, 4.2)
18	177.0		169.3		175.9		170.6		172.3	
19	22.9	0.96, d (6.6)	22.7	0.98, d (6.6)	22.9	0.97, d (6.6)	22.8	0.99, d (6.6)	22.6	0.94, d (6.5)
20	20.7	1.11, d (6.3)	20.0	1.07, d (6.6)	20.1	1.05, d (6.6)	20.4	1.06, d (6.6)	20.1	1.09, d (6.2)
1’	42.8	H_α_: 2.97, d (14.0) H_β_: 1.52, d (14.0)	65.0	3.60, s	39.4	H_α_: 2.17 d (16.2) H_β_: 1.99, d (16.2)	153.7	6.44, s	34.6	H_α_: 2.85, dd (15.0, 4.2) H_β_: 1.96, dd (15.0, 4.2)
2’	88.9		84.9		92.5		89.3		88.2	
3’	46.7	H_α_: 2.99, d (13.4) H_β_ 2.72, d (13.4)	46.1	H_α_: 3.32, d (13.2) H_β_: 3.10, d (13.2)	45.1	H_α_: 3.15, d (13.5) H_β_: 2.81, d (13.5)	45.8	H_α_: 3.26, d (12.6) H_β_: 3.19, d (12.6)	46.9	H_α_: 2.95, d (13.1) H_β_: 2.89, d (13.1)
4’	129.4		129.7		132.5		129.7		128.4	
5’	133.7	6.95, dd (8.4, 1.8)	130.4	7.08, dd (8.4, 1.8)	131.0	6.97, dd (8.4, 2.4)	129.7	6.94, dd (8.4, 1.8)	133.4	6.98, dd (8.4, 2.0)
6’	121.0	6.70, dd (8.4, 2.4)	120.0	6.91, dd (8.4, 2.4)	123.5	6.84, dd (8.4, 2.4)	119.0	6.90, dd (8.4, 2.4)	124.3	6.74, dd (7.8,1.8)
7’	159.1		160.6		159.7		162.0		157.9	
8’	125.2	6.94, dd (8.4, 2.4)	13.8	7.03, dd (8.4, 2.4)	123.3	7.02, dd (8.4, 2.4)	123.1	6.84, dd (8.4, 2.4)	120.2	7.02, dd (8.4, 1.8)
9’	134.0	6.96, dd (8.4, 1.8)	134.1	7.29, dd (8.4, 2.4)	133.4	7.16, dd (8.4, 2.4)	133.0	7.28, dd (8.4, 2.4)	131.9	7.07, dd (7.8, 2.0)
10’					49.7	3.24, s	-	-	-	-

*^a^* Data were recorded in CD_3_OD; *^b^* Data were recorded in CDCl_3_.

**Table 2:** Inhibitory effects of compounds **1**–**5** on NO production in LPS-induced RAW264.7 cells *^a^*.

	1	2	3	4	5	Resveratrol *^a^*
IC_50_± SD (μM)	45.8 ± 0.5	37.5 ± 0.4	12.8 ± 0.3	-- *^b^*	6.7 ± 0.3	36.0 ± 0.4

*^a^* Resveratrol and DMSO were used as the positive and negative control, respectively. *^b^* “--” denotes IC_50_ value ˃ 50 μM.

**Table 3:** Cytotoxicities of compounds **1**–**5** (IC_50_ values: μM) *^a^*.

Compound	NB4	U937
**1**	12.6 ± 0.2	13.9 ± 0.3
**2**	-- ^*b*^	-- *^b^*
**3**	18.4 ± 0.4	13.5 ± 0.1
**4**	11.9 ± 0.2	8.5 ± 0.2
**5**	6.4 ± 0.1	10.6 ± 0.2
Adriamycin	0.4 ± 0.1	0.2 ± 0.1

*^a^* Adriamysin and DMSO were used as the positive and negative control, respectively. *^b^* “--” denotes IC_50_ value ˃ 20 μM.

## Data Availability

Not applicable.

## Supplementary Materials

The following are available online at www.mdpi.com/article/10.3390/md20060375/s1, Figures S1–S44: The HRESIMS, 1 D, 2D NMR and ECD spectra of compounds **1**–**5.**
