# Supplementary material for: Xenoacremones D–H, Bioactive Tyrosine-decahydrofluorene Analogues from the Plant-Derived Fungus Xenoacremonium sinensis
Source: Mar Drugs. 2022 May 31;20(6):375. doi: 10.3390/md20060375 (PMC9227518; doi:10.3390/md20060375)
Supplement: Supplementary file 1 [file marinedrugs-20-00375-s001.zip › marinedrugs-1718987-supplementary.pdf]

Supporting Information

Xenoacremones D-H, bioactive tyrosine-decahydrofluorene analogues from the plant-derived fungus *Xenoacremonium sinensis*

Zhiguo Liu,[1,†] Li Liu,[1,†] Anqi Wang,[1] Li Li,[2] Sinan Zhao,[1] Yanan Wang,[2] Yi Sun[1,*]

[1] Institute of Chinese Materia Medica, China Academy of Chinese Medical Sciences, Beijing 100700, PR China

[2] Institute of Materia Medica, Chinese Academy of Medical Sciences & Peking Union Medical College, Beijing 100050, PR China

* Correspondence: ysun@icmm.ac.cn (Y. S.); Tel.: +86-10-64032656

† These authors contributed equally to this work.

Table of contents

Figure S1 The strain identification of *Xenoacremonium sinensis* 4

Figure S2 NOESY spectrum of compounds **4-5** in CD_3OD 5

Figure S3 ECD spectrum of compounds **4-5** in MeOH..5

Figure S4 ^{1}H NMR spectrum of xenoacremone D (**1**) in CD_3OD..6

Figure S5 ^{13}C NMR spectrum of xenoacremone D (**1**) in CD_3OD..6

Figure S6 HSQC spectrum of xenoacremone D (**1**) in CD_3OD 7-8

Figure S7 HMBC spectrum of xenoacremone D (**1**) in CD_3OD..8-9

Figure S8 ^{1}H-^{1}H COSY spectrum of xenoacremone D (**1**) in CD_3OD..............................10-11

Figure S9 TCOSY spectrum of xenoacremone D (**1**) in CD_3OD..11

Figure S10 NOESY spectrum of xenoacremone D (**1**) in CD_3OD 12-13

Figure S11 ECD spectrum of xenoacremone D (**1**) (in MeOH) 13

Figure S12 HRESI-MS spectrum of xenoacremone D (**1**)..14

Figure S13 ^{1}H NMR spectrum of xenoacremone E (**2**) in CD_3OD 15

Figure S14 ^{13}C NMR spectrum of xenoacremone E (**2**) in CD_3OD..15

Figure S15 HSQC spectrum of xenoacremone E (**2**) in CD_3OD 16-17

Figure S16 HMBC spectrum of xenoacremone E (**2**) in CD_3OD 17-18

Figure S17 ^{1}H-^{1}H COSY spectrum of xenoacremone E (**2**) in CD_3OD 19-20

Figure S18 NOESY spectrum of xenoacremone E (**2**) in CD_3OD......................................20-21

Figure S19 ECD spectrum of xenoacremone E (**2**) (in MeOH) 22

Figure S20 HRESI-MS spectrum of xenoacremone E (**2**) 22

Figure S21 ^{1}H NMR spectrum of xenoacremone F (**3**) in CD_3OD 23

Figure S22 ^{13}C NMR spectrum of xenoacremone F (**3**) in CD_3OD..23

Figure S23 HSQC spectrum of xenoacremone F (**3**) in CD_3OD 24-25

Figure S24 HMBC spectrum of xenoacremone F (**3**) in CD_3OD 25-26

Figure S25 ^{1}H-^{1}H COSY spectrum of xenoacremone F (**3**) in CD_3OD.............................27-28

Figure S26 NOESY spectrum of xenoacremone F (**3**) in CD_3OD 28-29

Figure S27 ECD spectrum of xenoacremone F (**3**) (in MeOH) 30

Figure S28 HRESI-MS spectrum of xenoacremone F (**3**)..30

Figure S29 ^{1}H NMR spectrum of xenoacremone G (**4**) in CD_3OD..31

Figure S30 ^{13}C NMR spectrum of xenoacremone G (**4**) in CD_3OD.......................................31

Figure S31 HSQC spectrum of xenoacremone G (**4**) in CD_3OD.......................................32-33

Figure S32 HMBC spectrum of xenoacremone G (**4**) in CD_3OD......................................33-34

Figure S33 ^{1}H-^{1}H COSY spectrum of xenoacremone G (**4**) in CD_3OD............................35-36

Figure S34 NOESY spectrum of xenoacremone G (**4**) in CD_3OD 36-37

Figure S35 ECD spectrum of xenoacremone G (**4**) (in MeOH) 38

Figure S36 HRESI-MS spectrum of xenoacremone G (**4**)..38

Figure S37 ^{1}H NMR spectrum of xenoacremone H (**5**) in CD_3OD 39

Figure S38 ^{13}C NMR spectrum of xenoacremone H (**5**) in CD_3OD.......................................39

Figure S39 HSQC spectrum of xenoacremone H (**5**) in CD_3OD 40-41

Figure S40 HMBC spectrum of xenoacremone H (**5**) in CD_3OD 41-42

Figure S41 ^{1}H-^{1}H COSY spectrum of xenoacremone H (**5**) in CD_3OD............................43-44

Figure S42 NOESY spectrum of xenoacremone H (**5**) in CD_3OD....................................44-45

Figure S43 ECD spectrum of xenoacremone H (**5**) (in MeOH) 46

Figure S44 HRESI-MS spectrum of xenoacremone H (**5**) 46

Conformation calculations of ECD spectra of **1-3**..47-63

Figure S1 The strain of *Xenoacremonium sinensis* and the identification

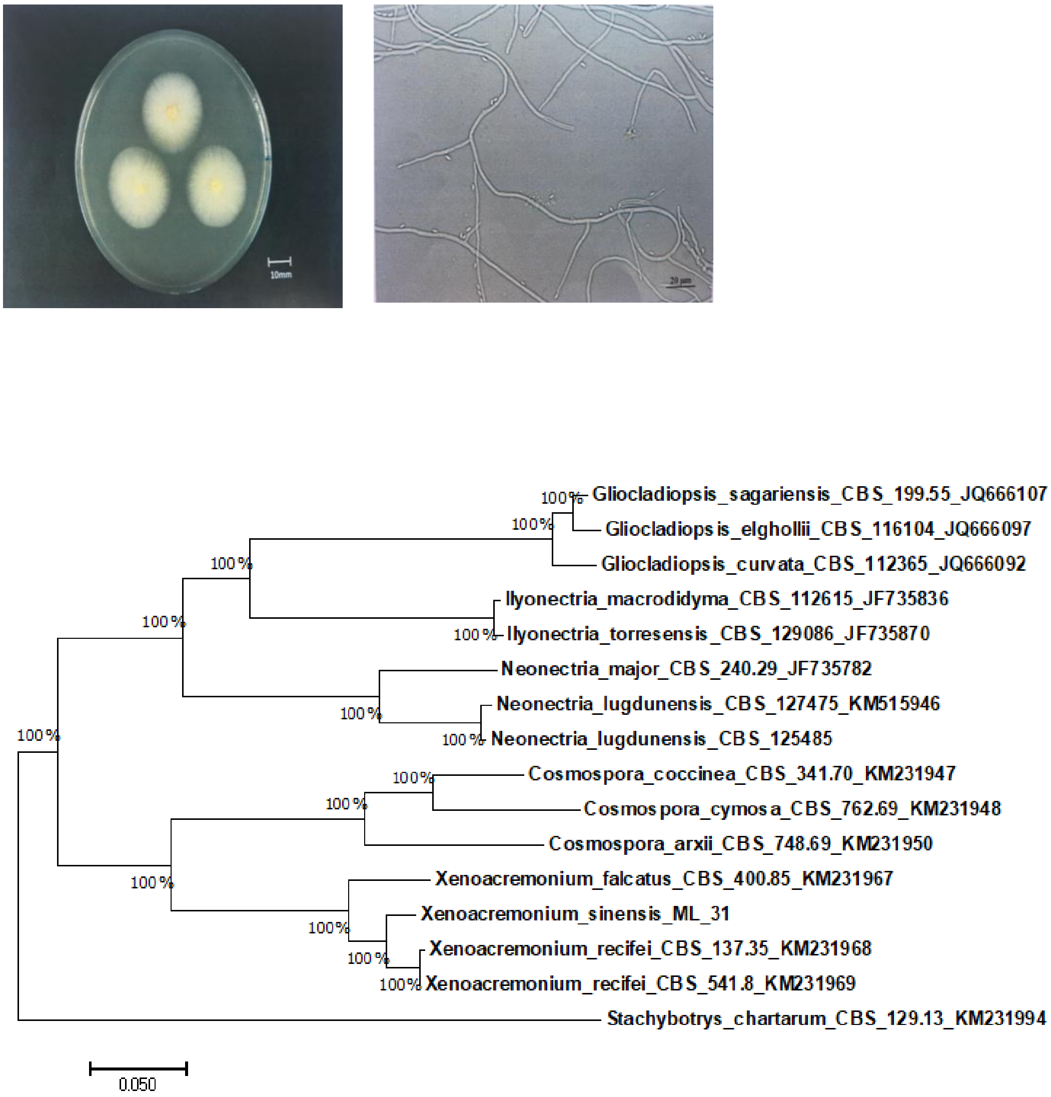

Figure S2 Key NOE correlations of compounds **4-5**.

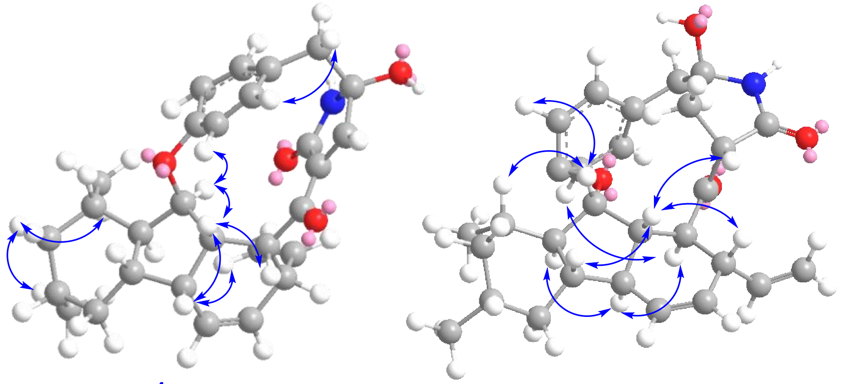

4 **5**

Figure S3 Experimental and calculated ECD of compounds **4-5** in MeOH.

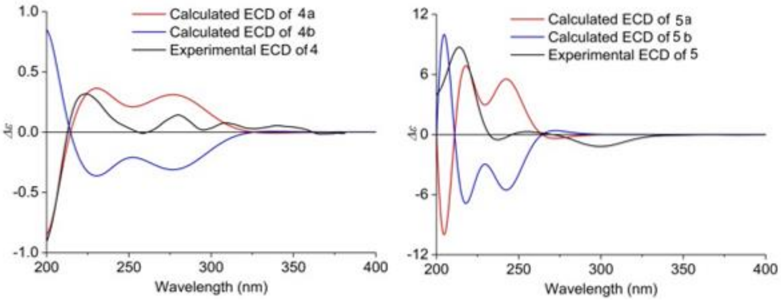

Figure S4 ^{1}H NMR spectrum of xenoacremone D (**1**) in CD_3OD

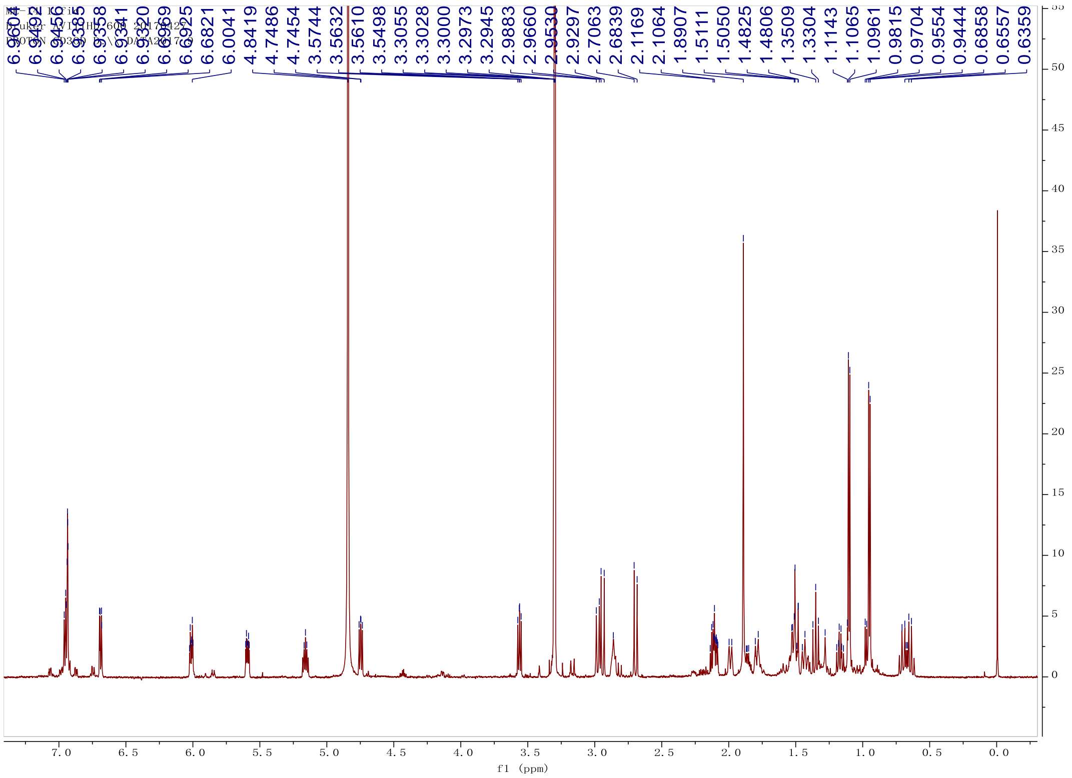

Figure S5 ^{13}C NMR spectrum of xenoacremone D (**1**) in CD_3OD

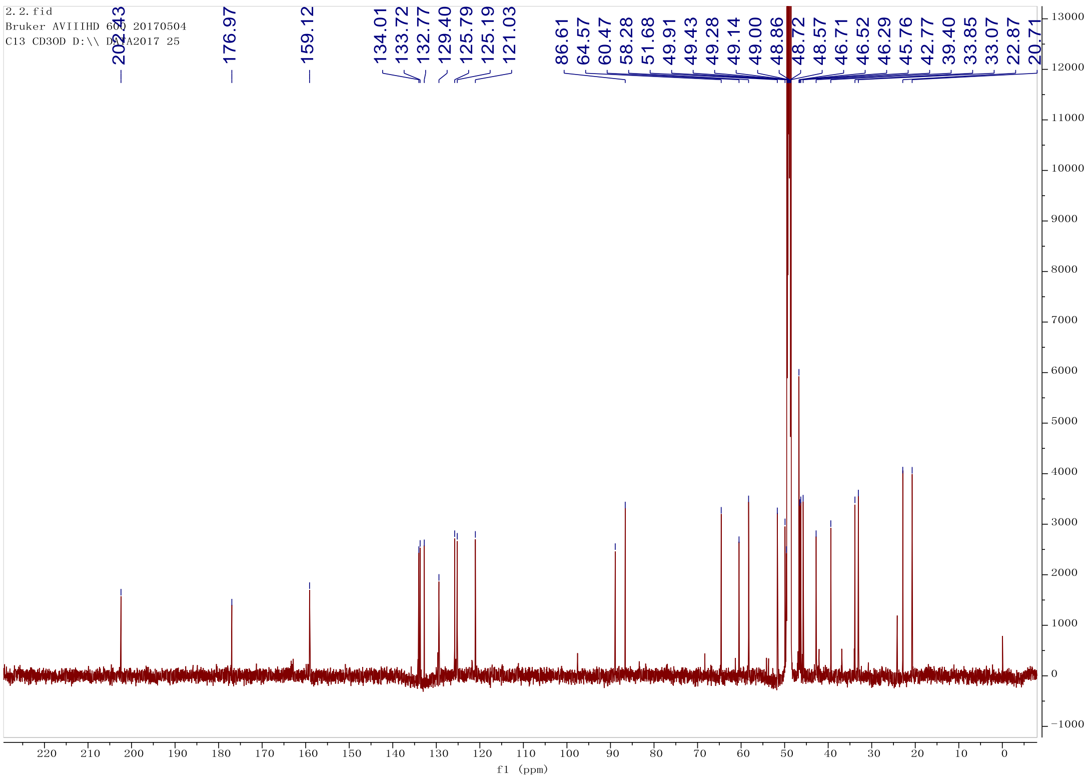

Figure S6 HSQC spectrum of xenoacremone D (**1**) in CD_3OD

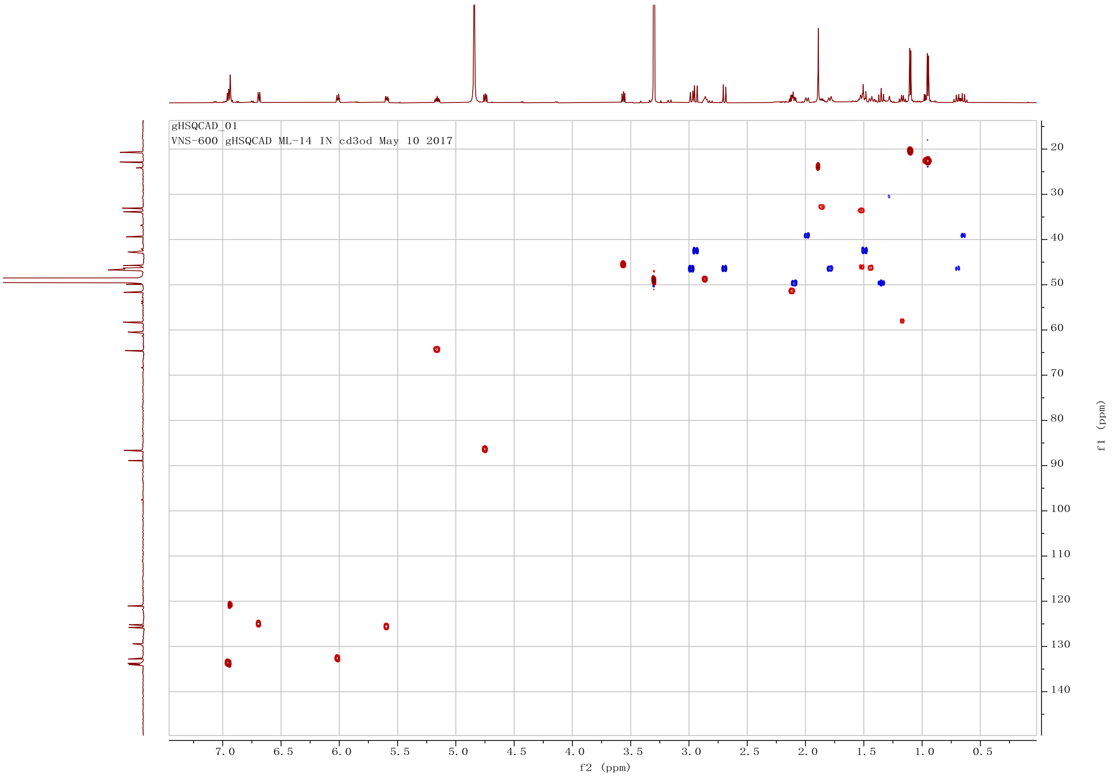

enlarged figure

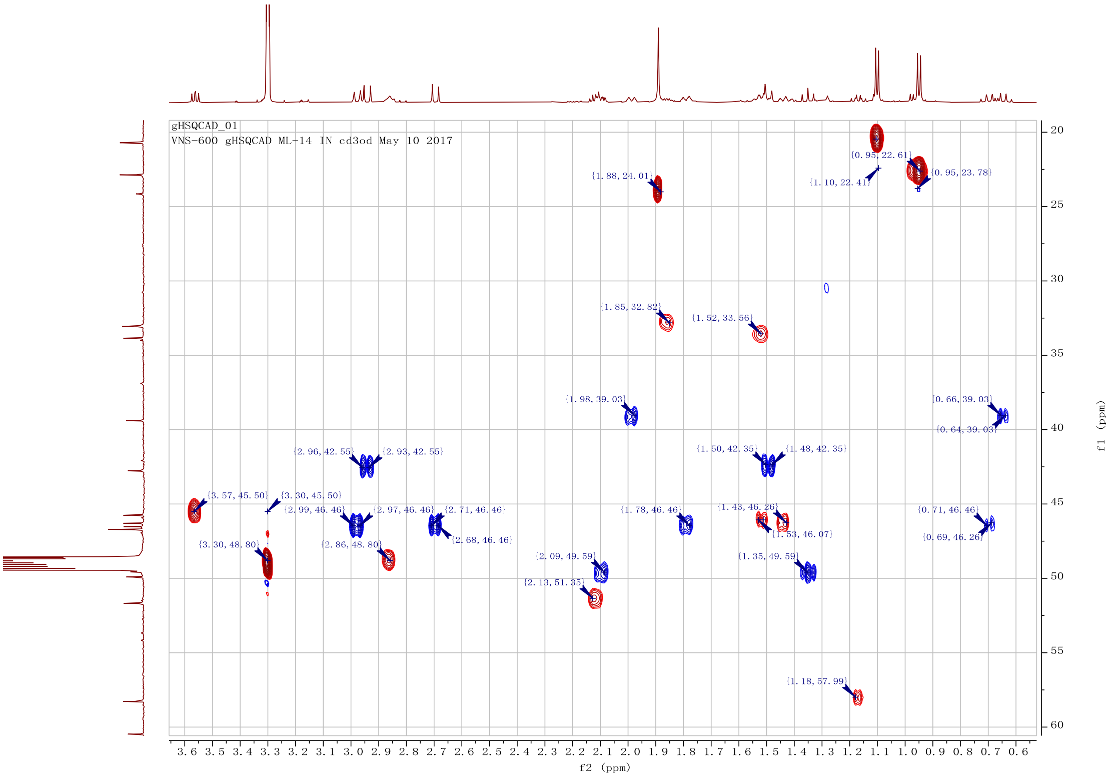

enlarged figure

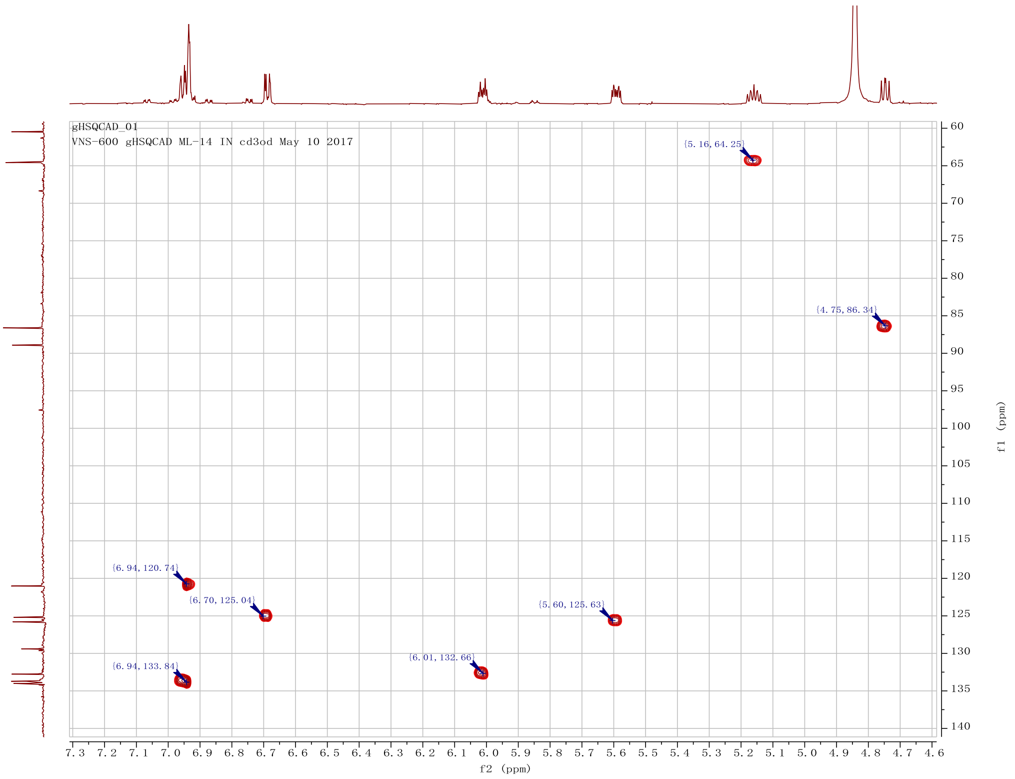

Figure S7 HMBC spectrum of xenoacremone D (**1**) in CD_3OD

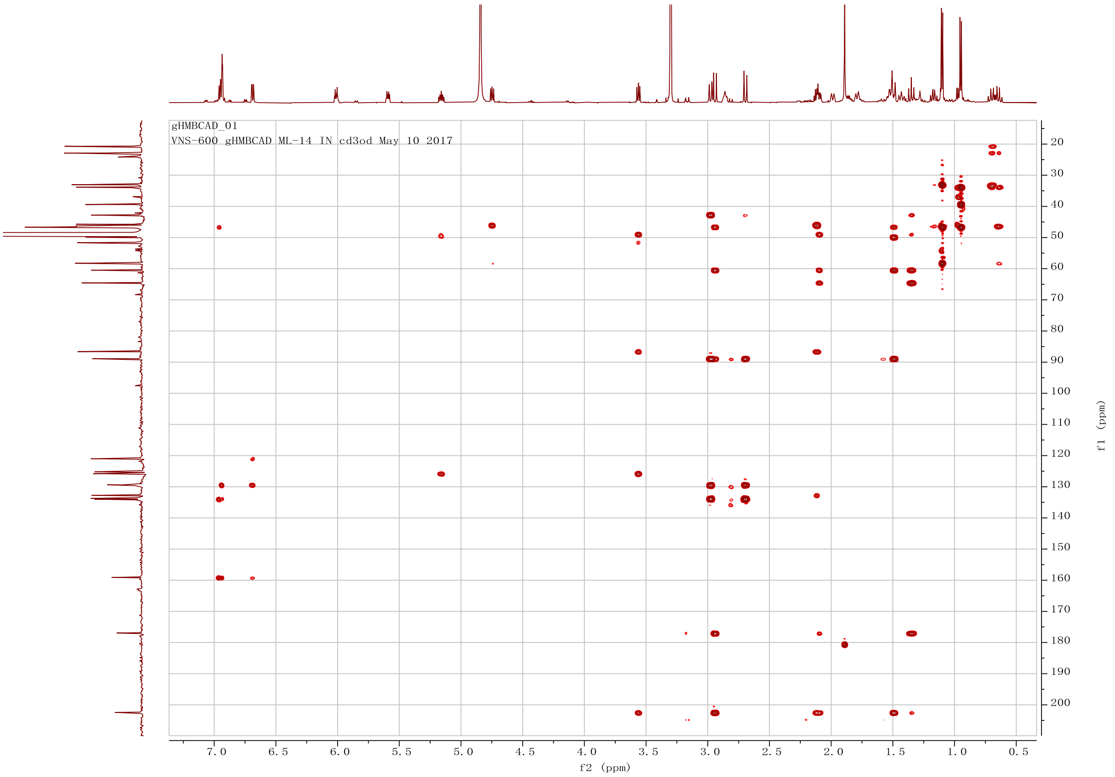

enlarged figure

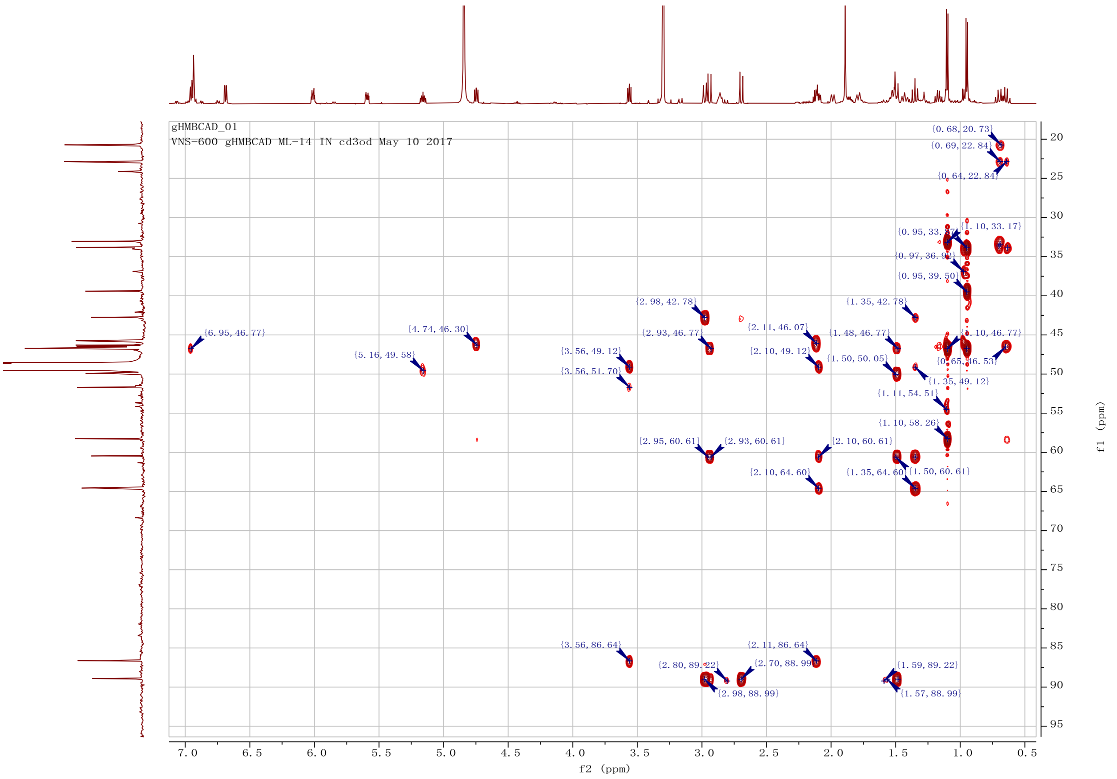

enlarged figure

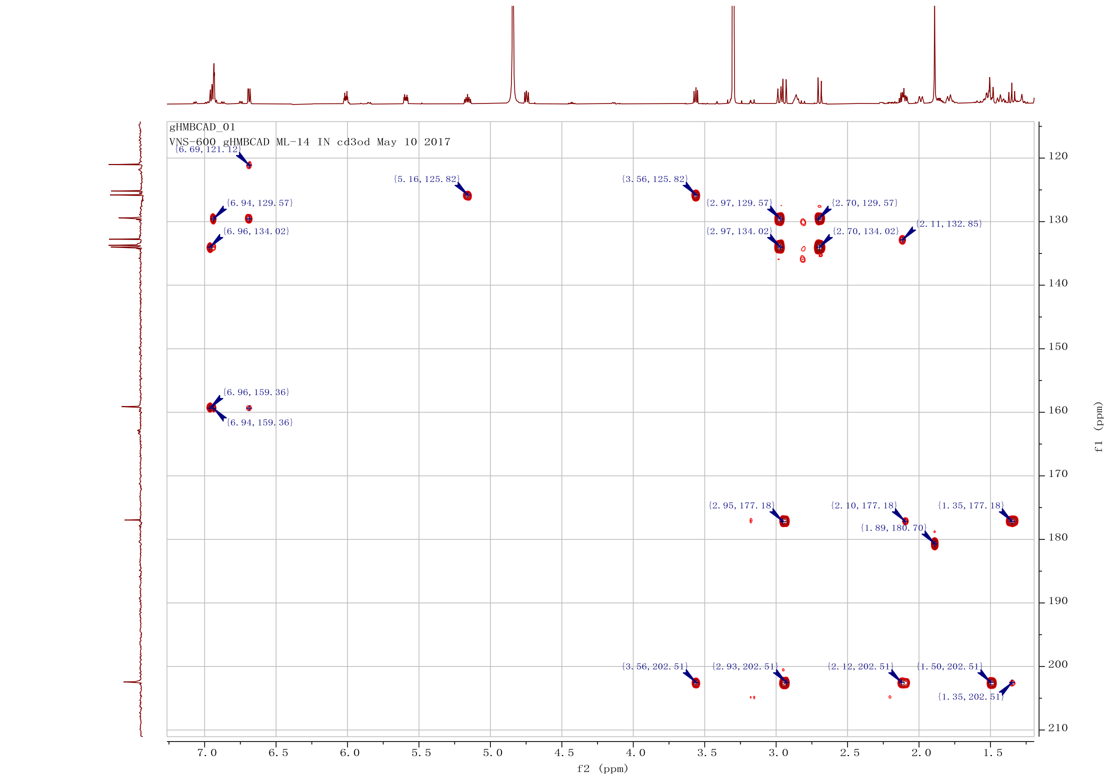

Figure S8 ^{1}H-^{1}H COSY spectrum of xenoacremone D (**1**) in CD_3OD

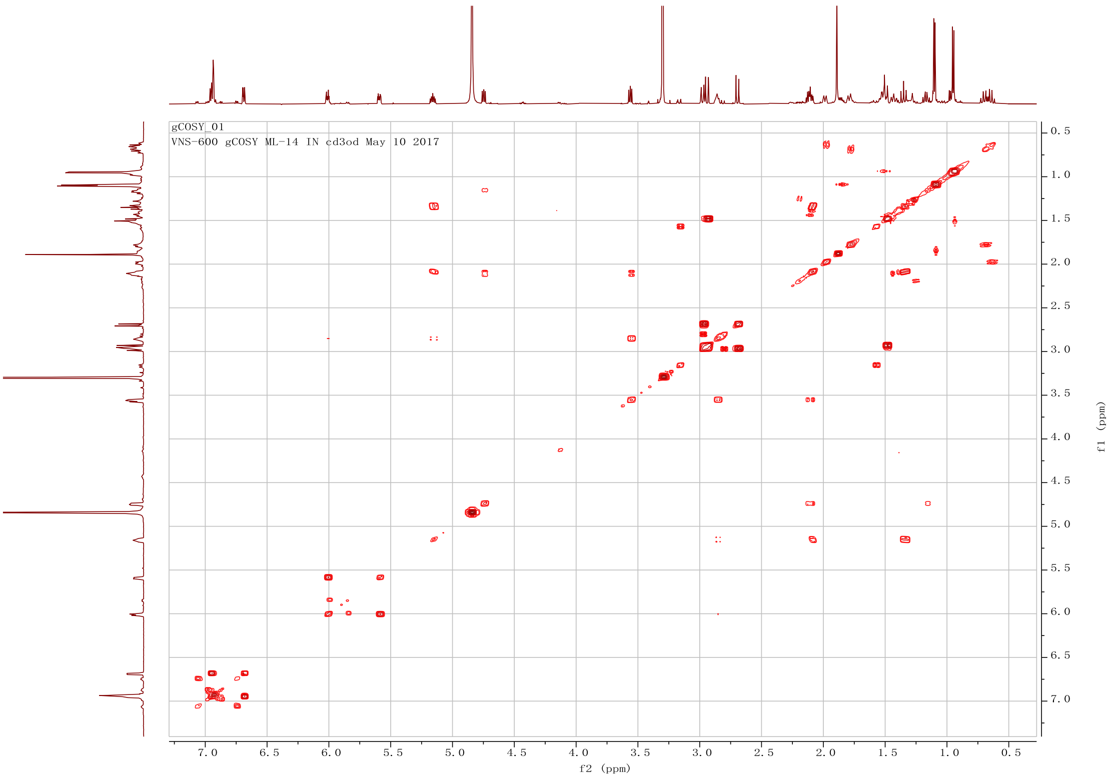

enlarged figure

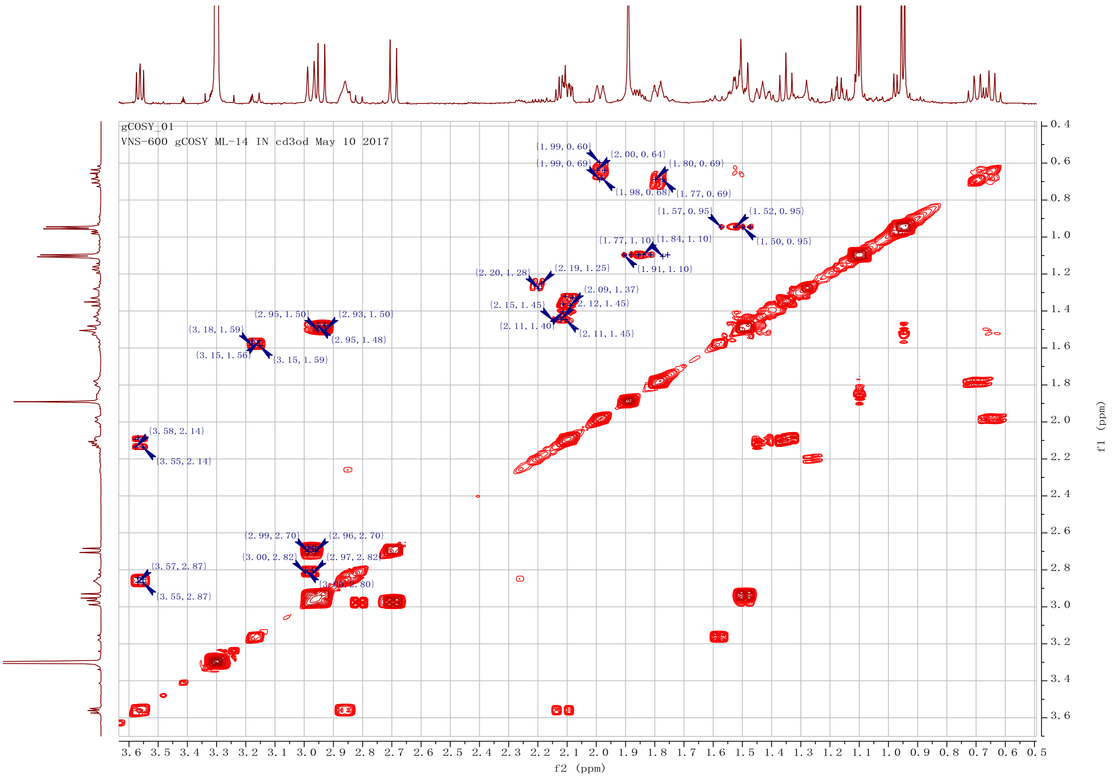

Enlarged figure

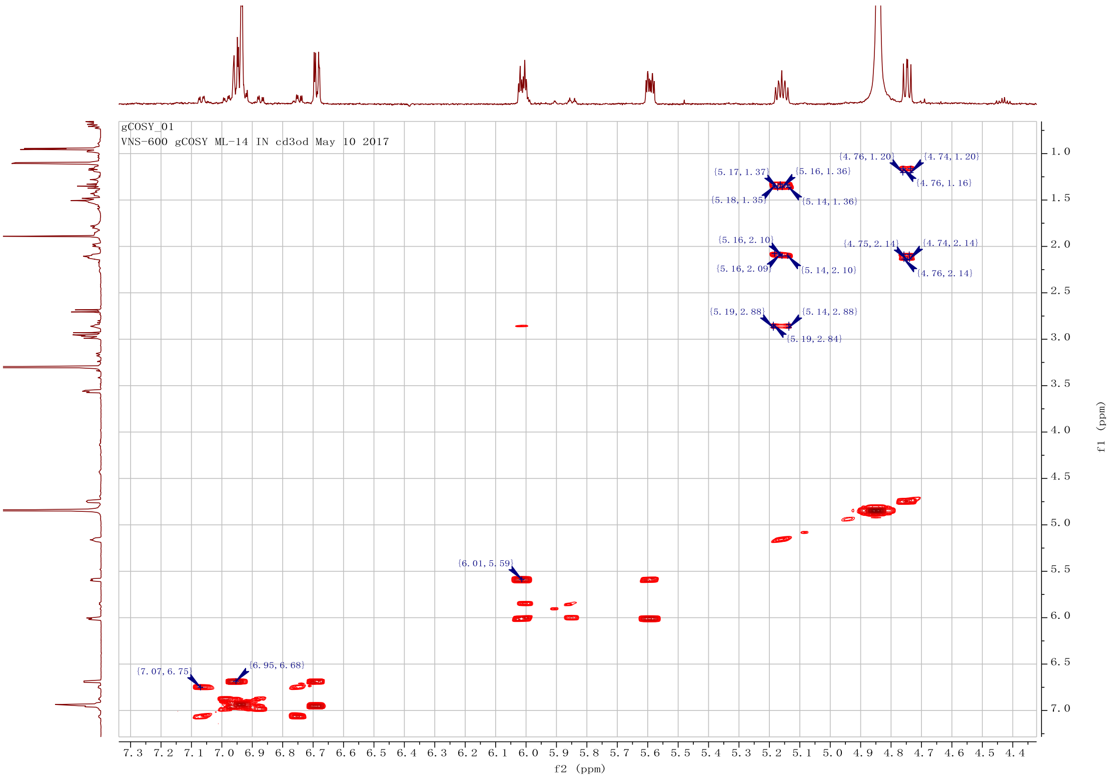

Figure S9 TOCSY spectrum of xenoacremone D (**1**) in CD_3OD

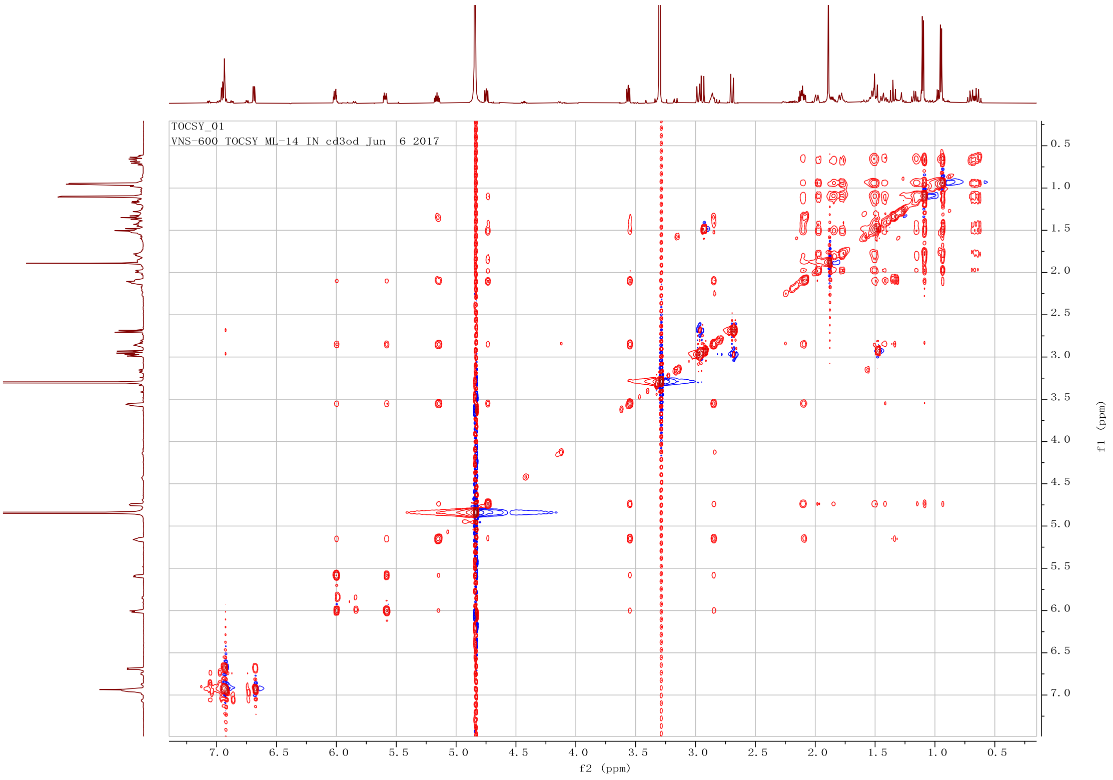

Figure S10 NOESY spectrum of xenoacremone D (**1**) in CD_3OD

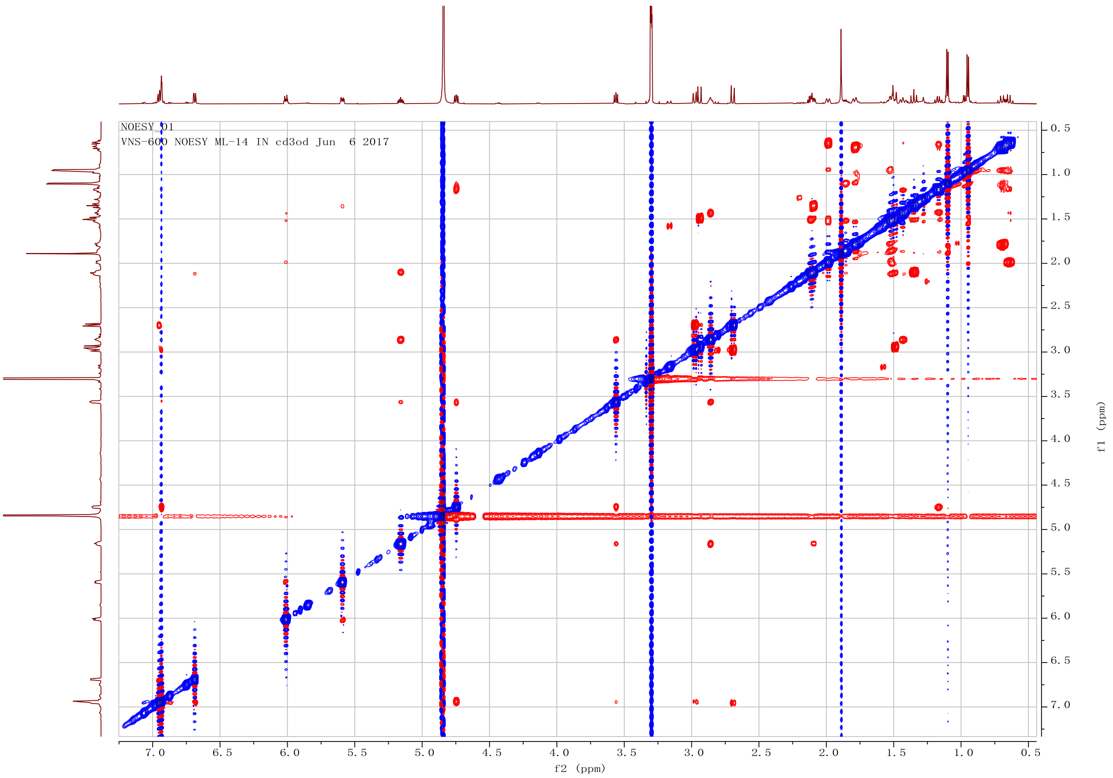

Enlarged figure

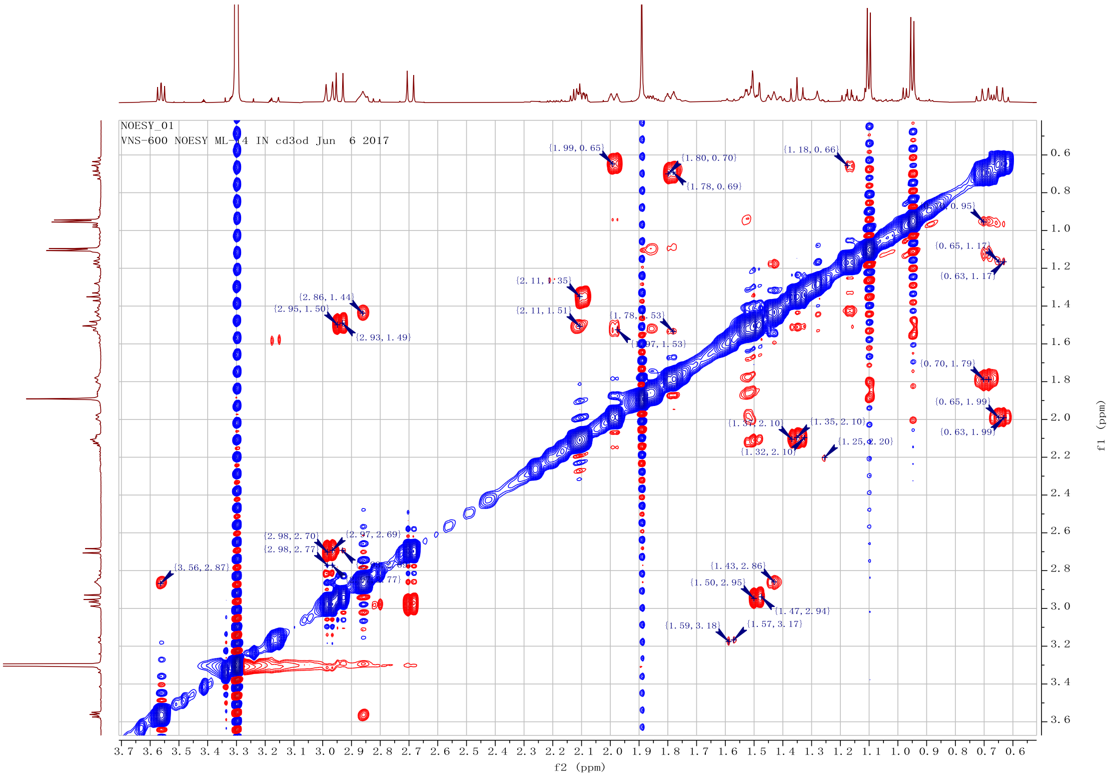

Enlarged figure

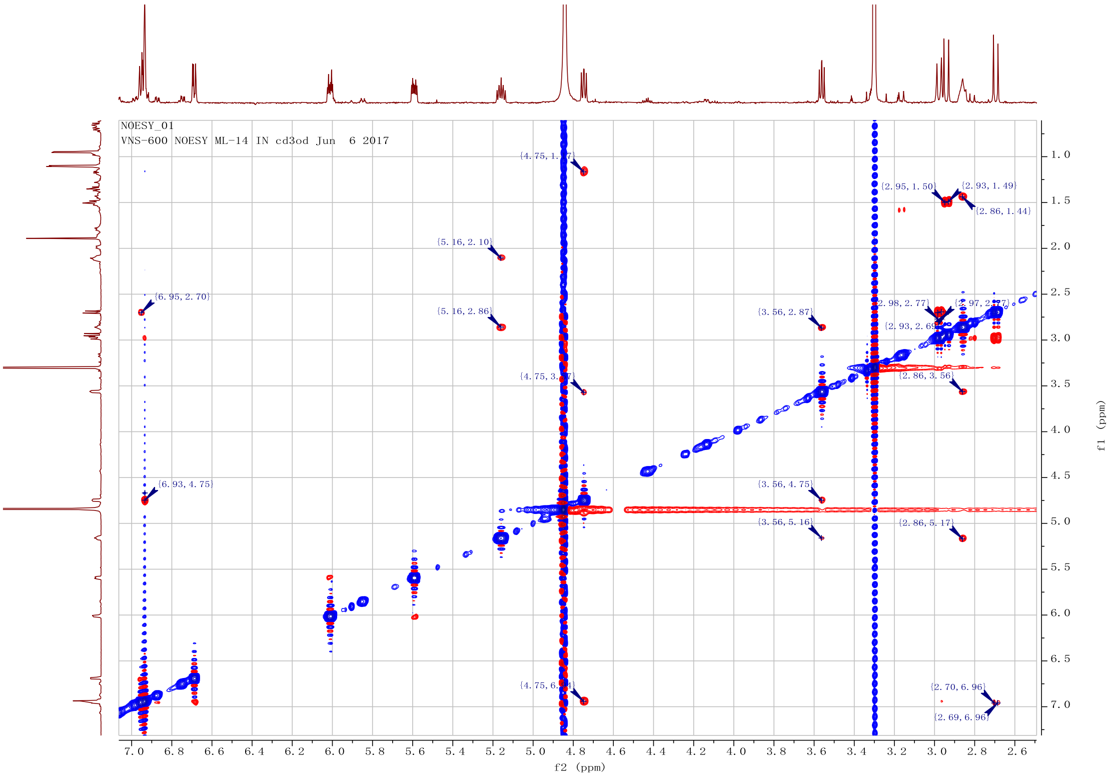

Figure S11 ECD spectrum of xenoacremone D (**1**) (in MeOH)

TDDFT theory, *ω*B3LYP functional and 6-311G(d,p) level of theory, and methanol was solvent for structural optimization. Compound **1** have 2 conformations. Cam-B3LYP functional and TZVP level calculate 70 excited states. The calculated result of **1** is consistent with the experimental result, and the absolute configuration of **1** is confirmed as shown in the figure below (σ=0.30eV).

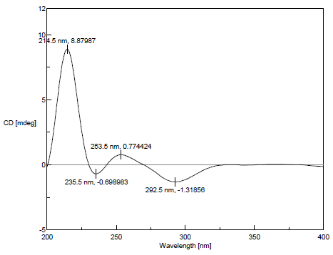

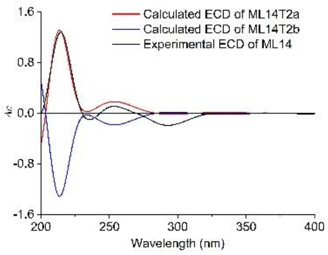

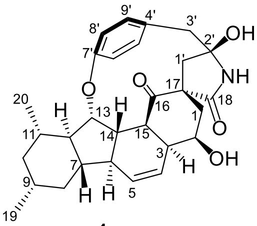

1

Figure S12 HR ESIMS data of xenoacremone D (**1**)

Composition	i-FIT Confidence (%)	m/z RMS (PPM)	Intensity RMS (%)	Predicted m/z	m/z error (PPM)	m/z error (mDa)	DBE
C29H35NO5	1.055278	3.733254	14.295821	478.258800	3.877003	1.850313	13.000000

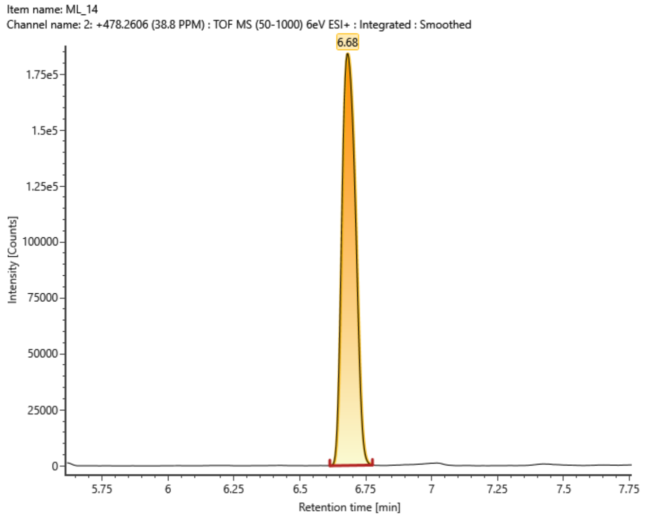

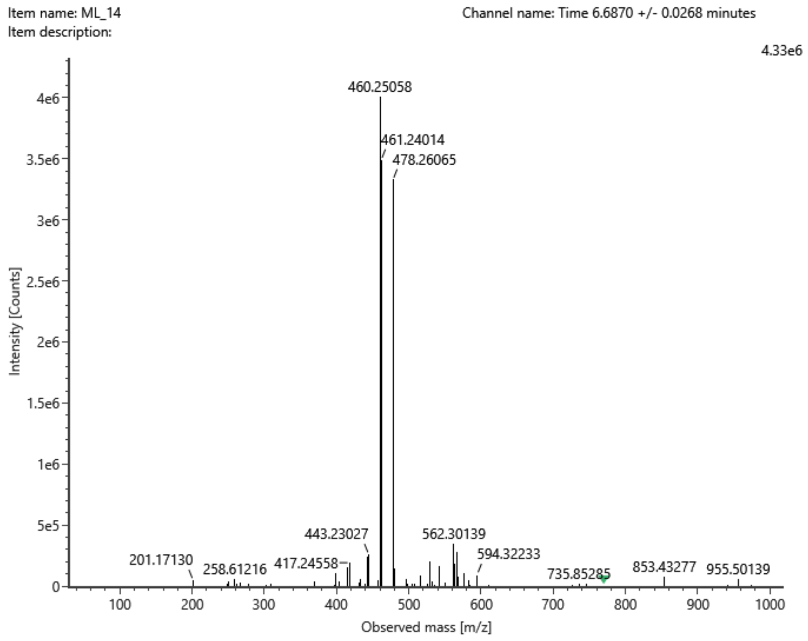

Figure S13 ^{1}H NMR spectrum of xenoacremone E (**2**) in CD_3OD

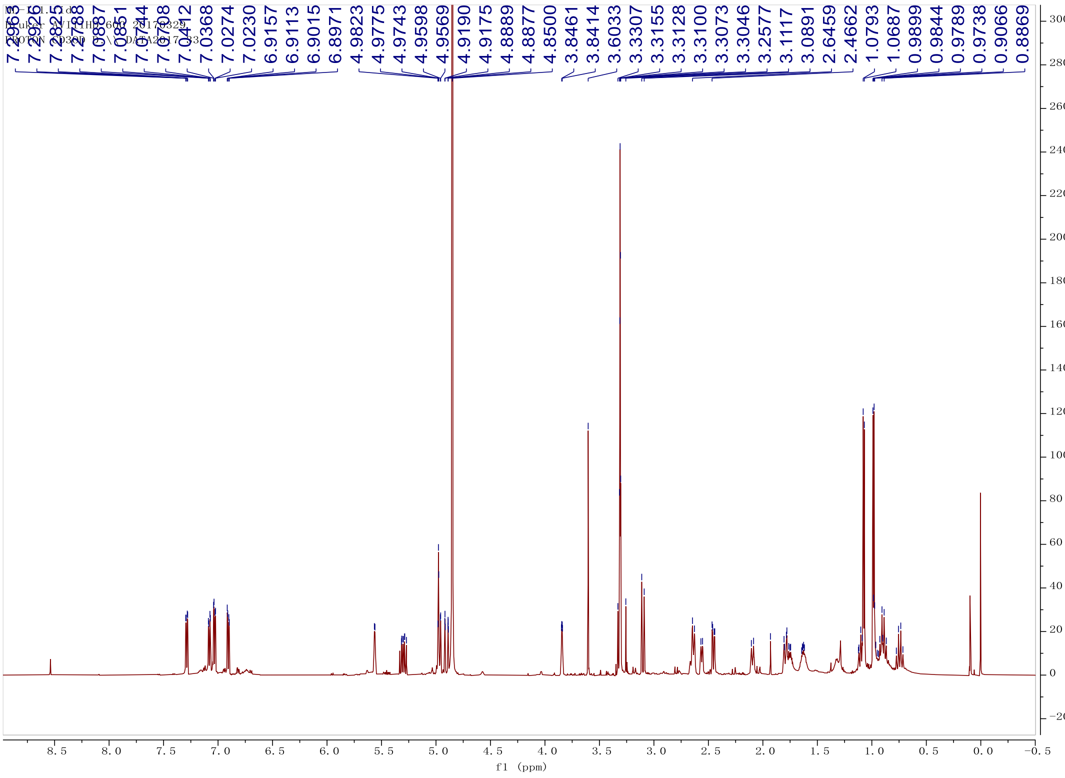

Figure S14 ^{13}C NMR spectrum of xenoacremone E (**2**) in CD_3OD

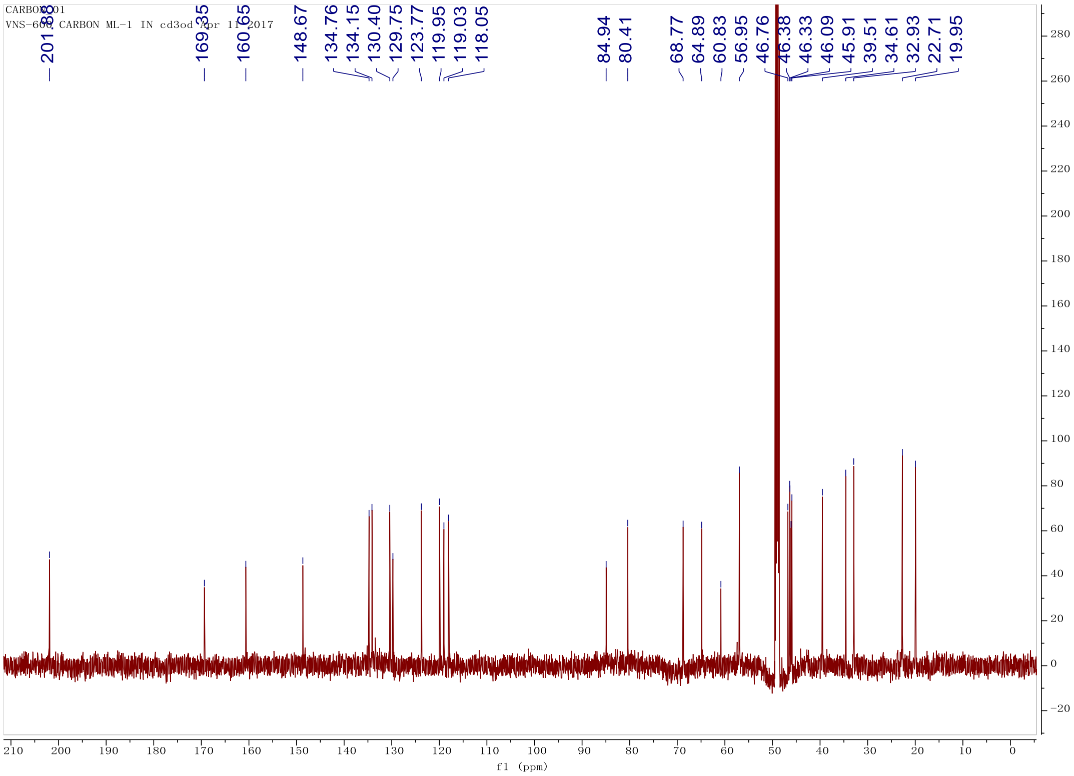

Figure S15 HSQC spectrum of xenoacremone E (**2**) in CD_3OD

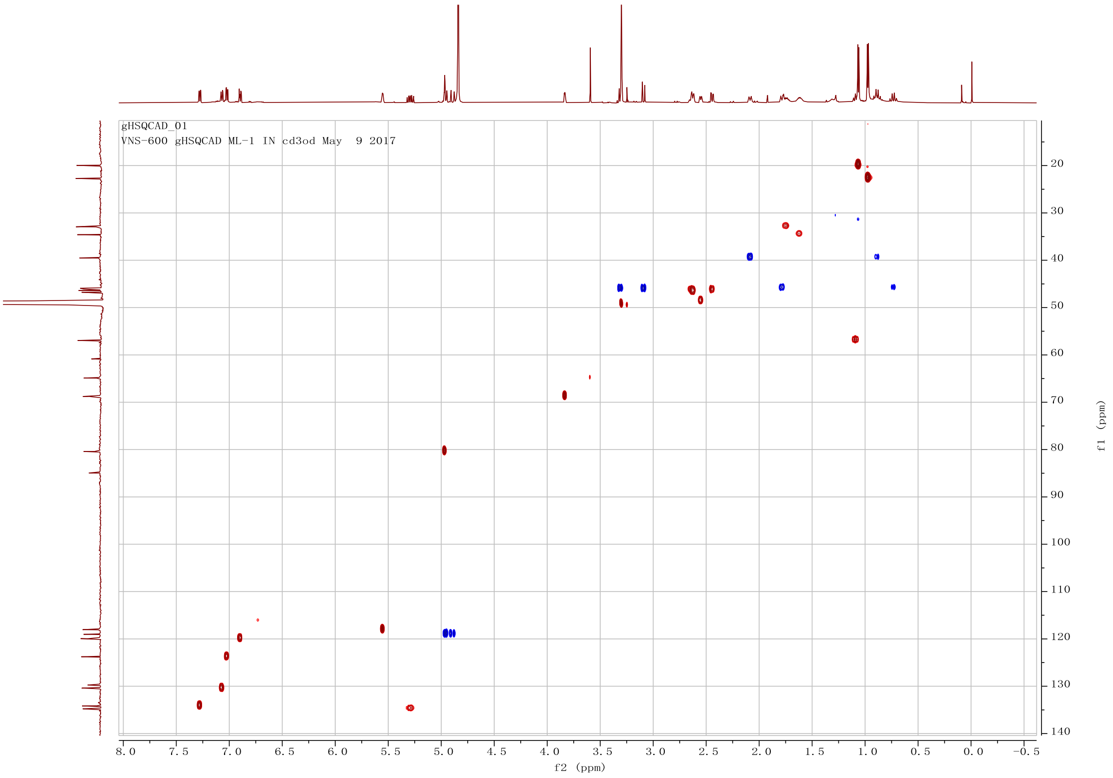

Enlarged figure

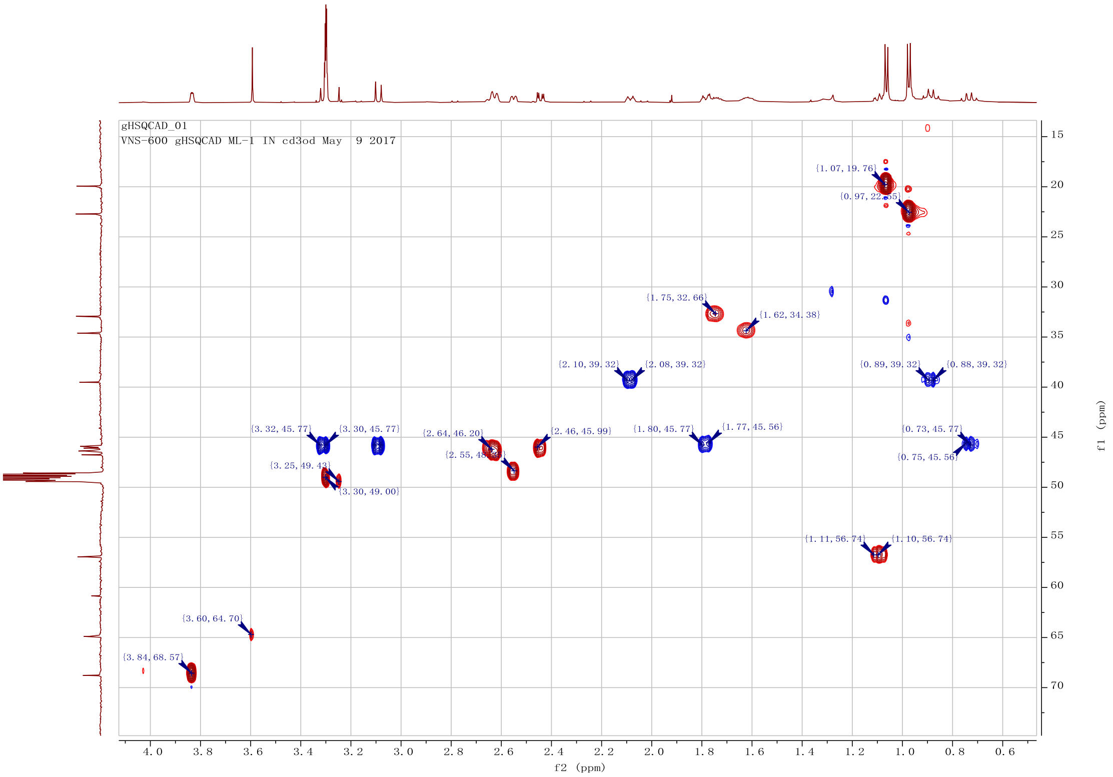

Enlarged figure

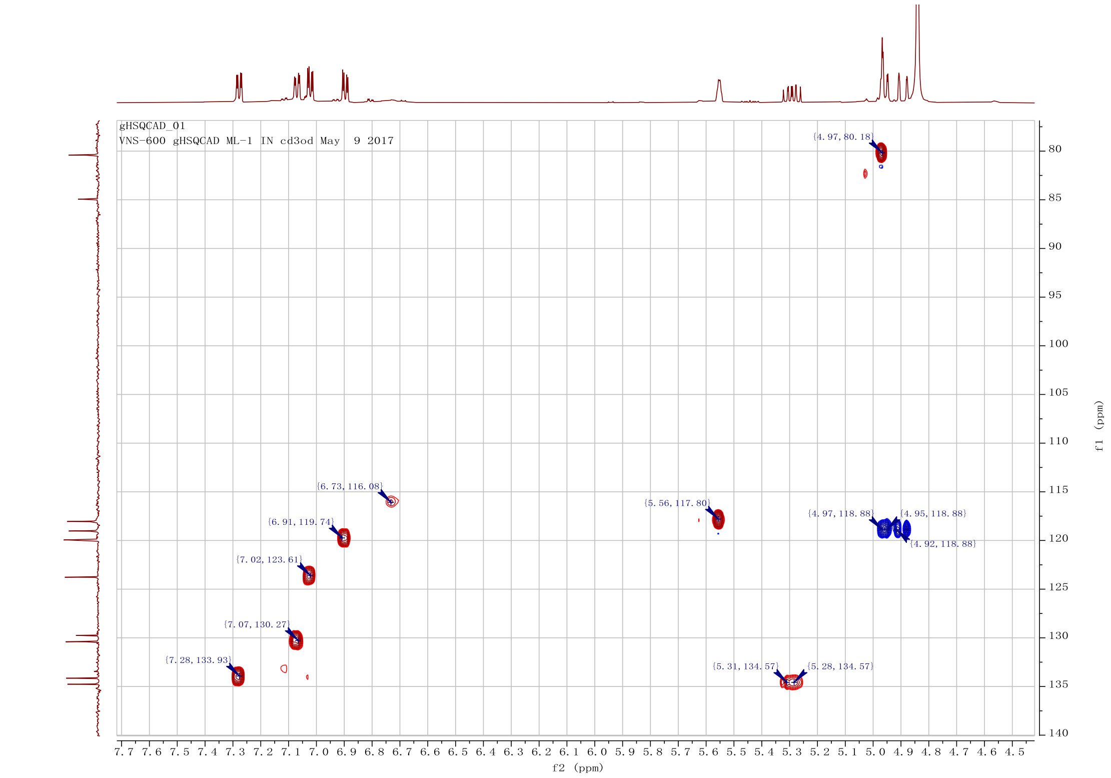

Figure S16 HMBC spectrum of xenoacremone E (**2**) in CD_3OD

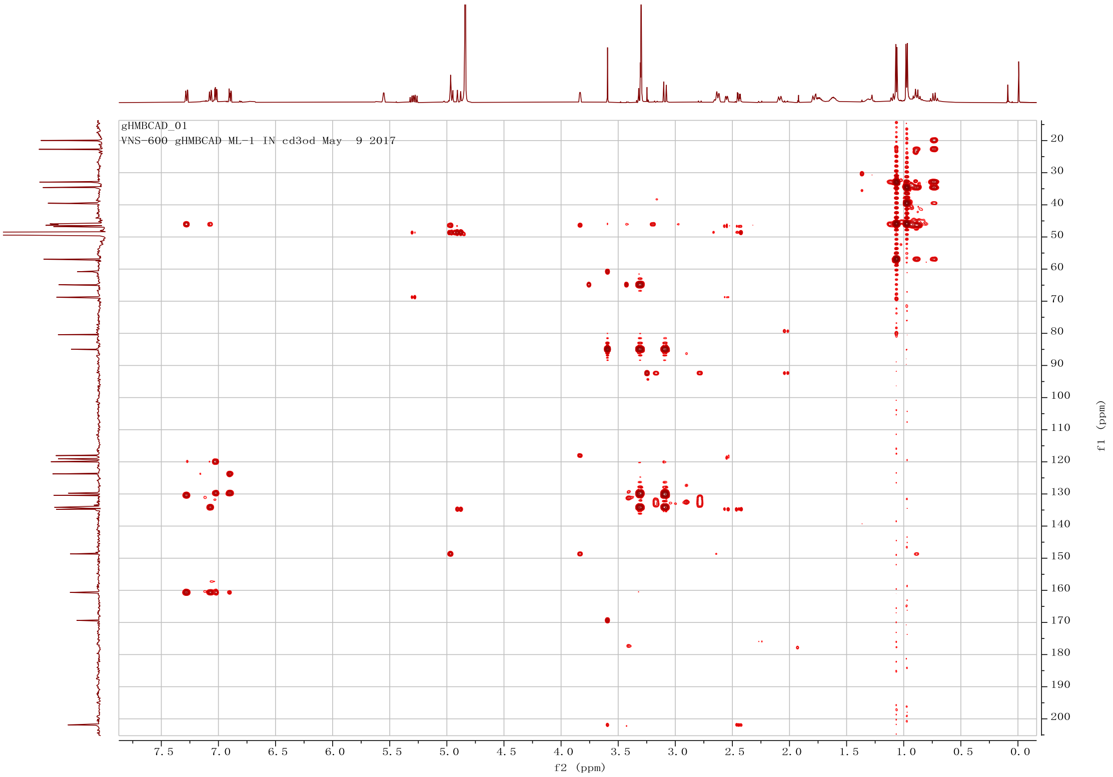

Enlarged figure

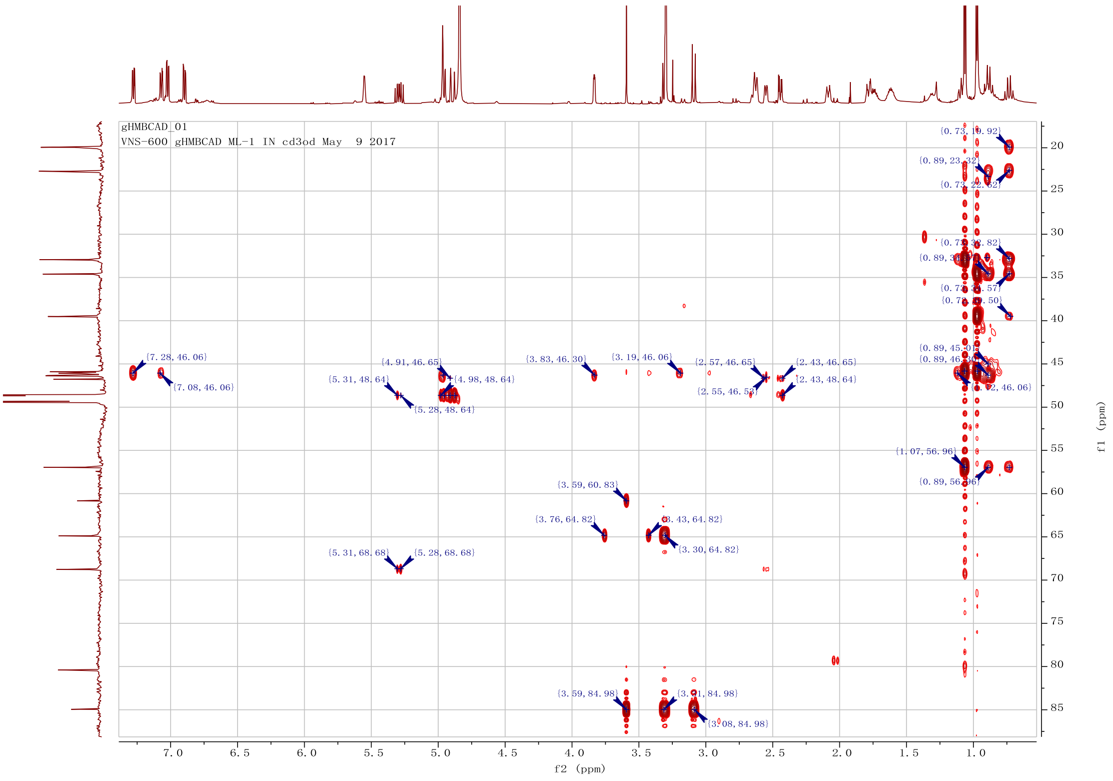

Enlarged figure

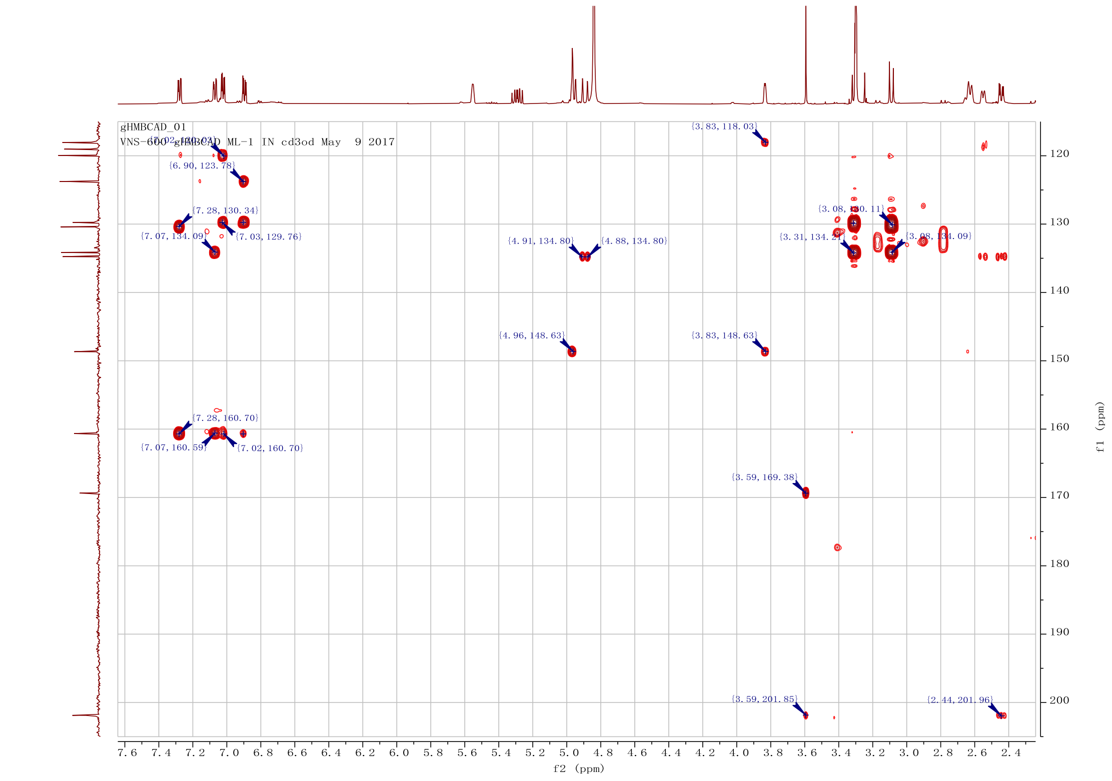

Figure S17 ^1H-^1H COSY spectrum of xenoacremone E (**2**) in CD_3OD

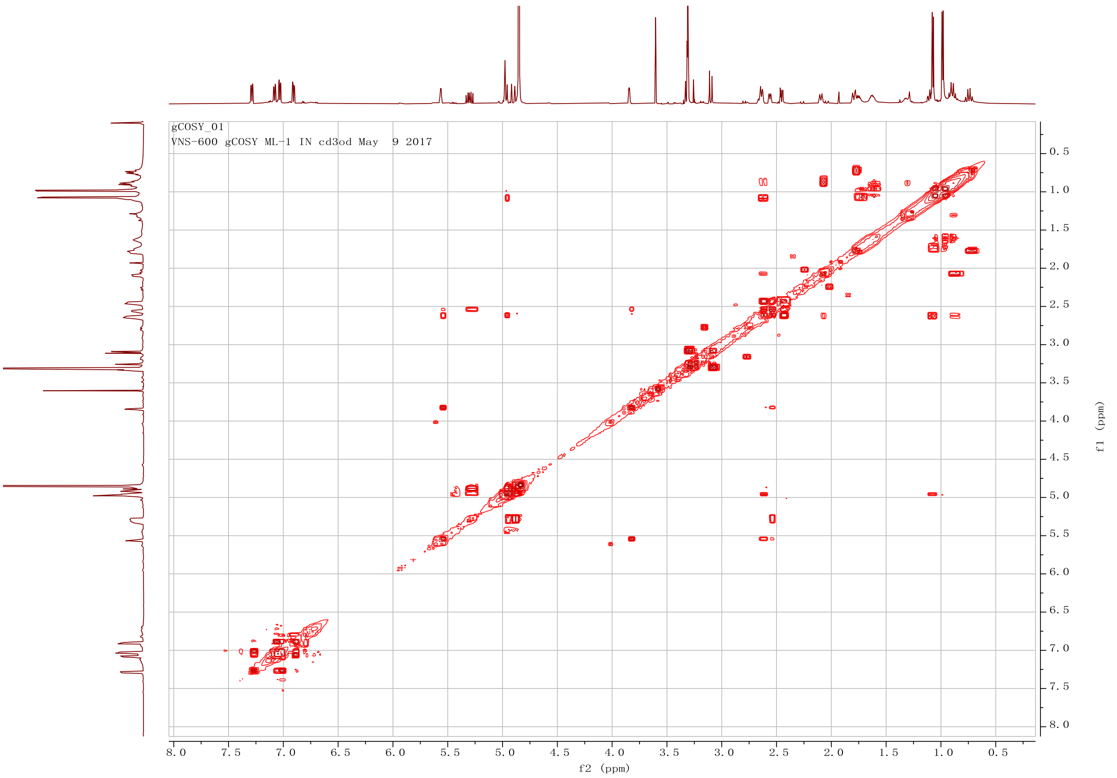

Enlarged figure

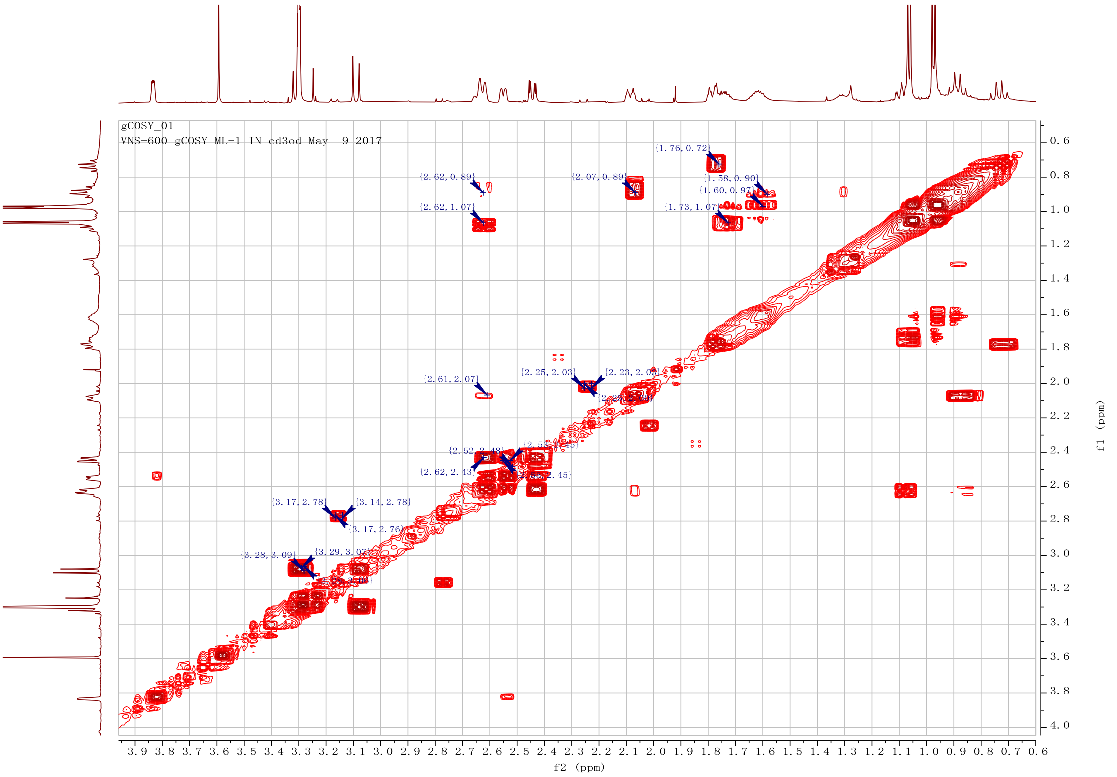

Enlarged figure

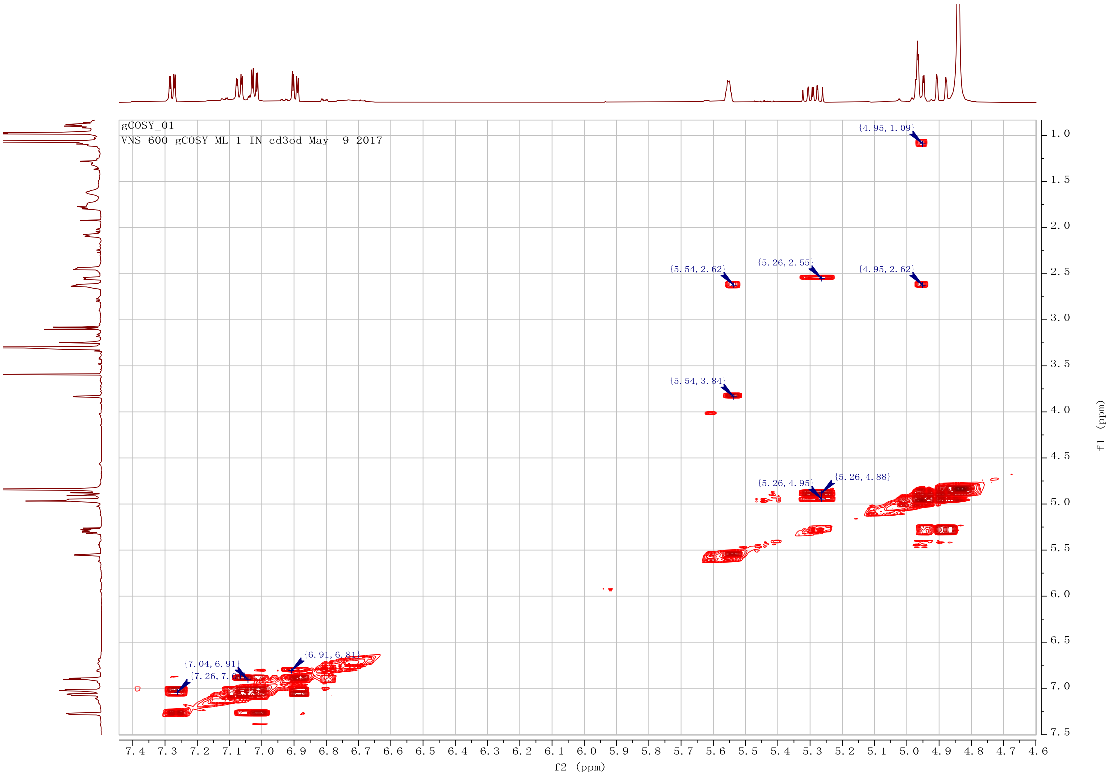

Figure S18 NOESY spectrum of xenoacremone E (**2**) in CD_3OD

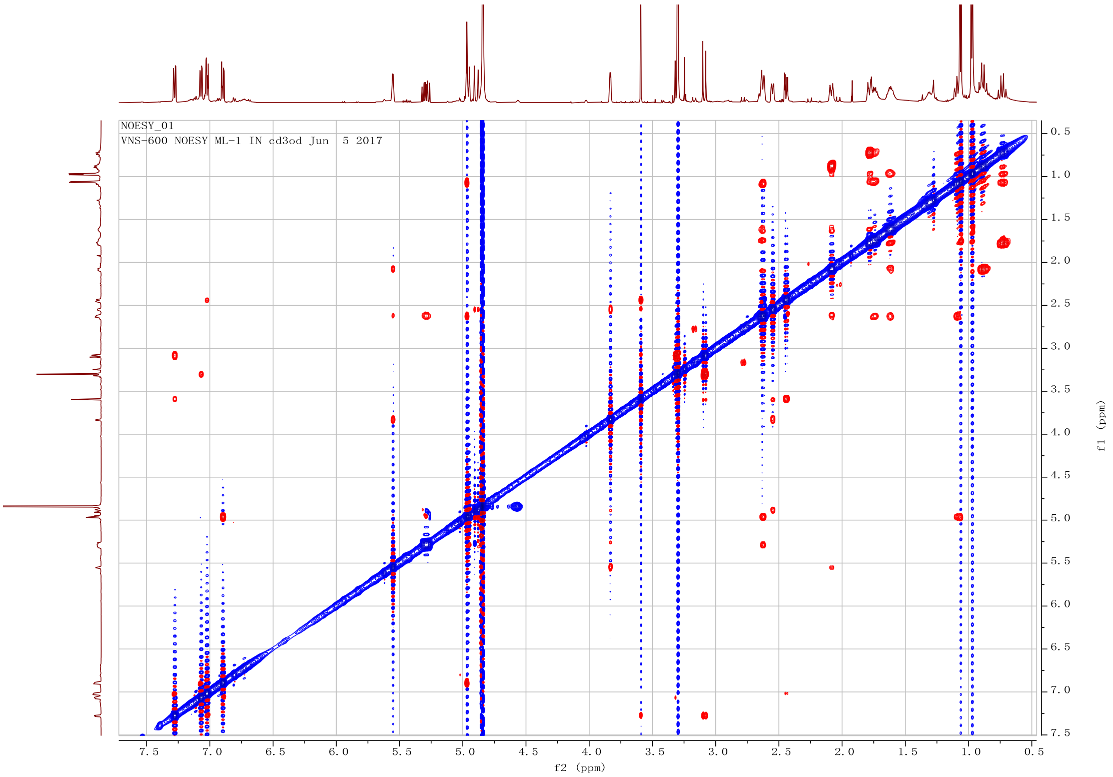

Enlarged figure

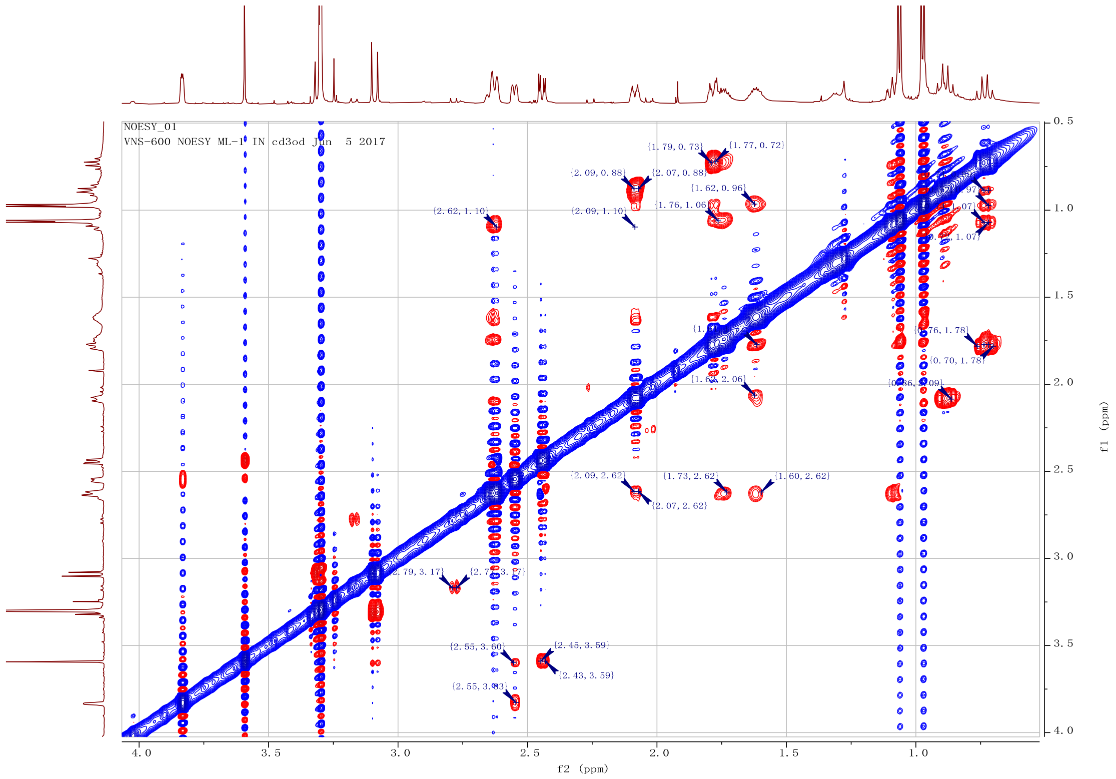

Enlarged figure

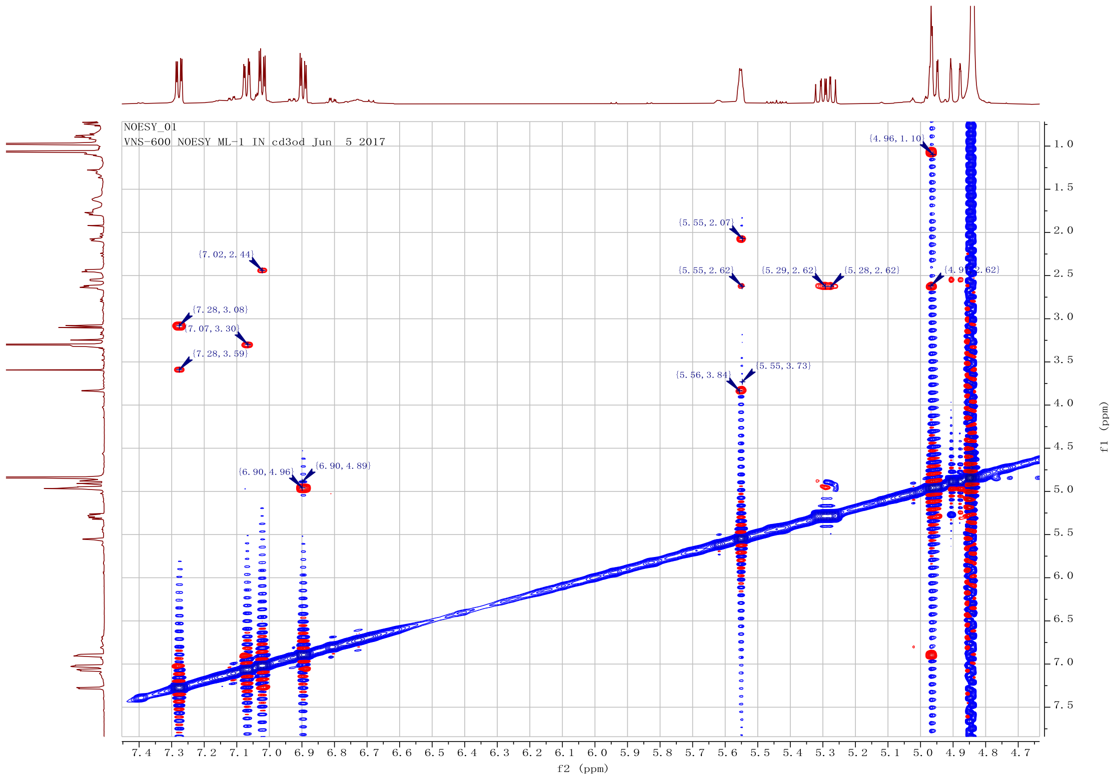

Figure S19 ECD spectrum of xenoacremone E (**2**) in MeOH

TDDFT theory, ωB97XD functional and TZVP level of theory, **2** has 2 conformations. Cam-B3LYP functional and TZVP level calculate 70 excited states, the solvent is methanol, σ=0.30eV, the result is shown in the figure.

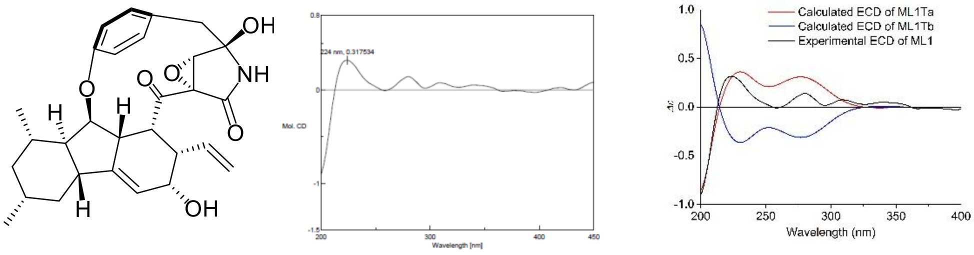

Figure S20 HR ESIMS spectrum of xenoacremone E (**2**)

Composition	i-FIT Confidence (%)	m/z RMS (PPM)	Intensity RMS (%)	Predicted m/z	m/z error (PPM)	m/z error (mDa)	DBE
C29H33NO6	82.992332	3.097868	7.701164	492.238064	2.983831	1.465754	14.000000

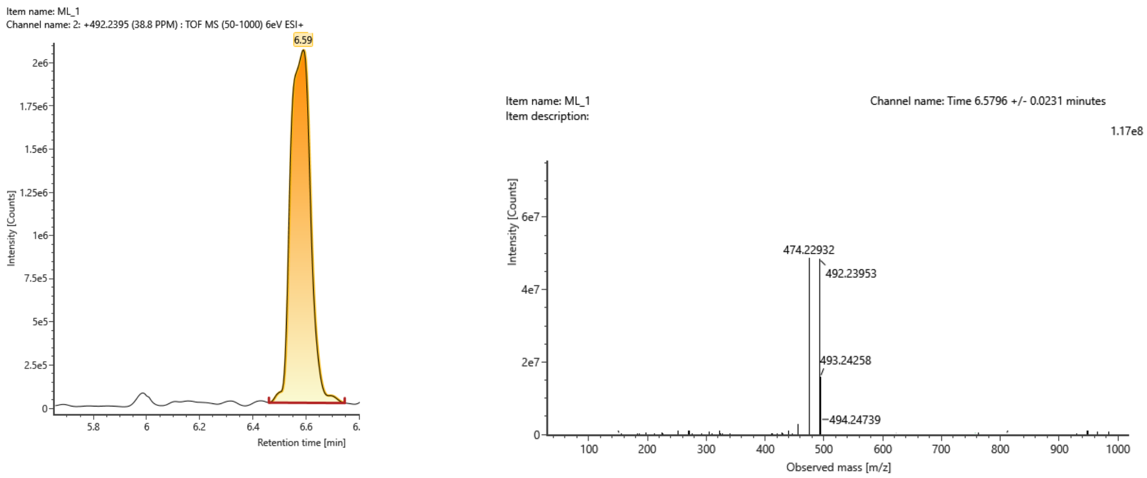

Figure S21 ^{1}H NMR spectrum of xenoacremone F (**3**) in CD_3OD

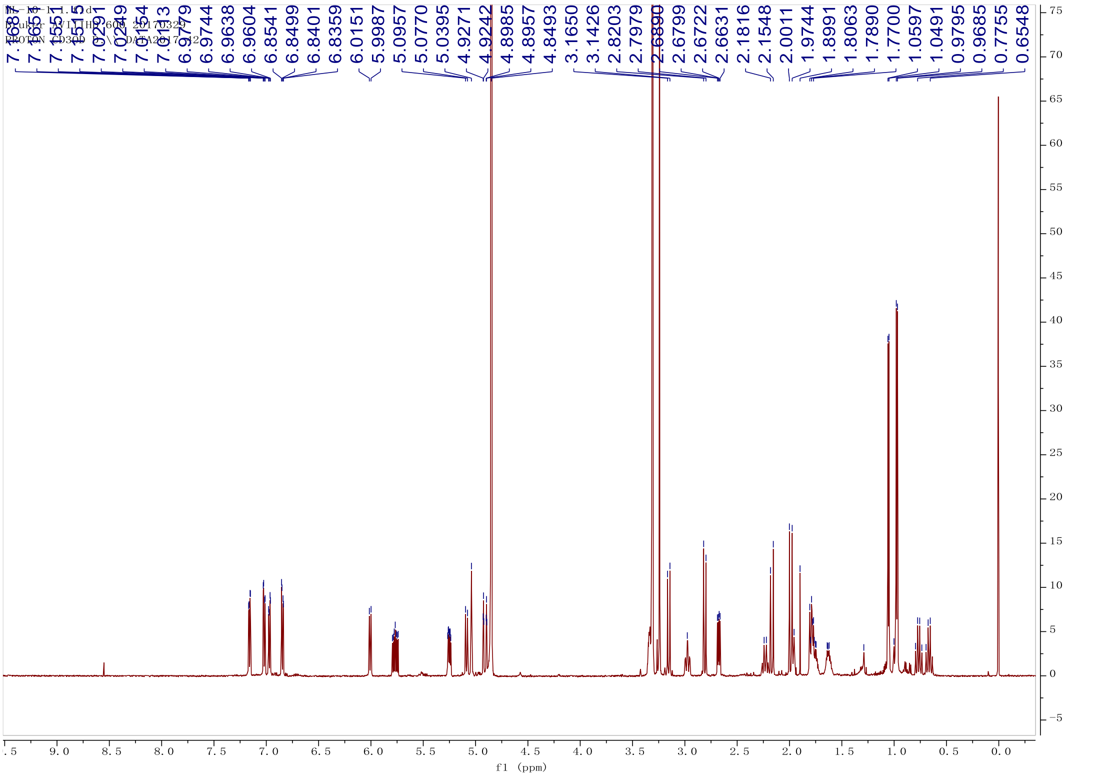

Figure S22 ^{13}C NMR spectrum of xenoacremone F (**3**) in CD_3OD

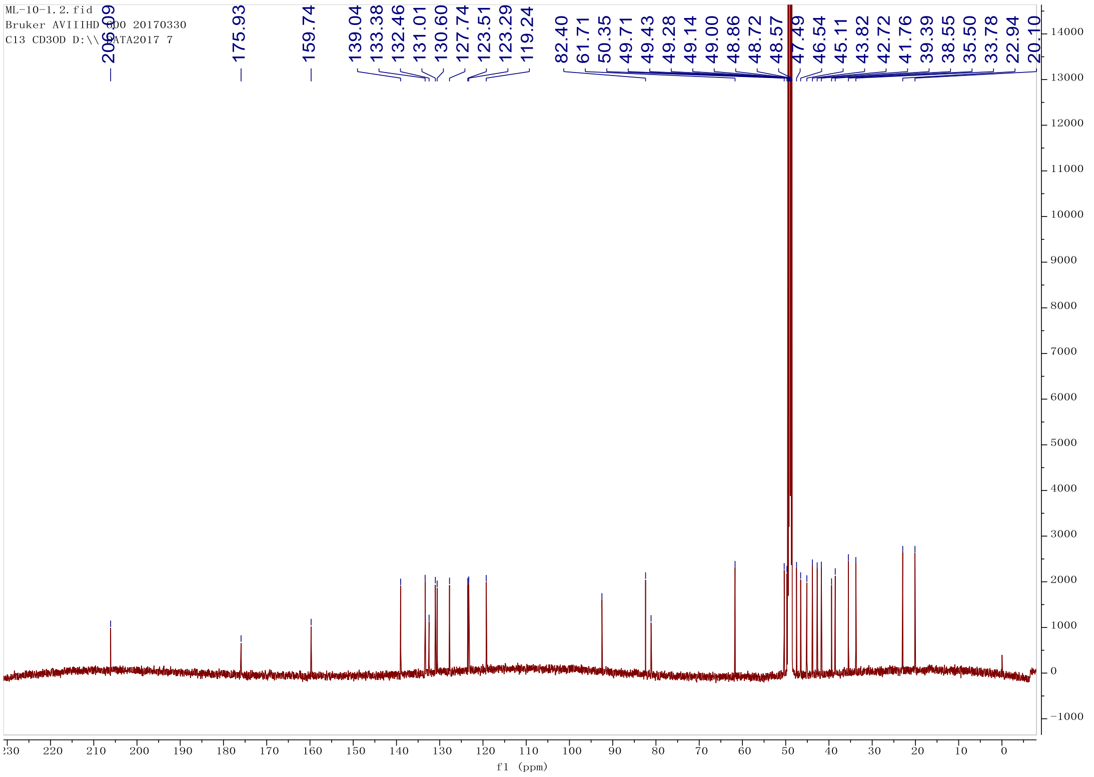

Figure S23 HSQC spectrum of xenoacremone F (**3**) in CD_3OD

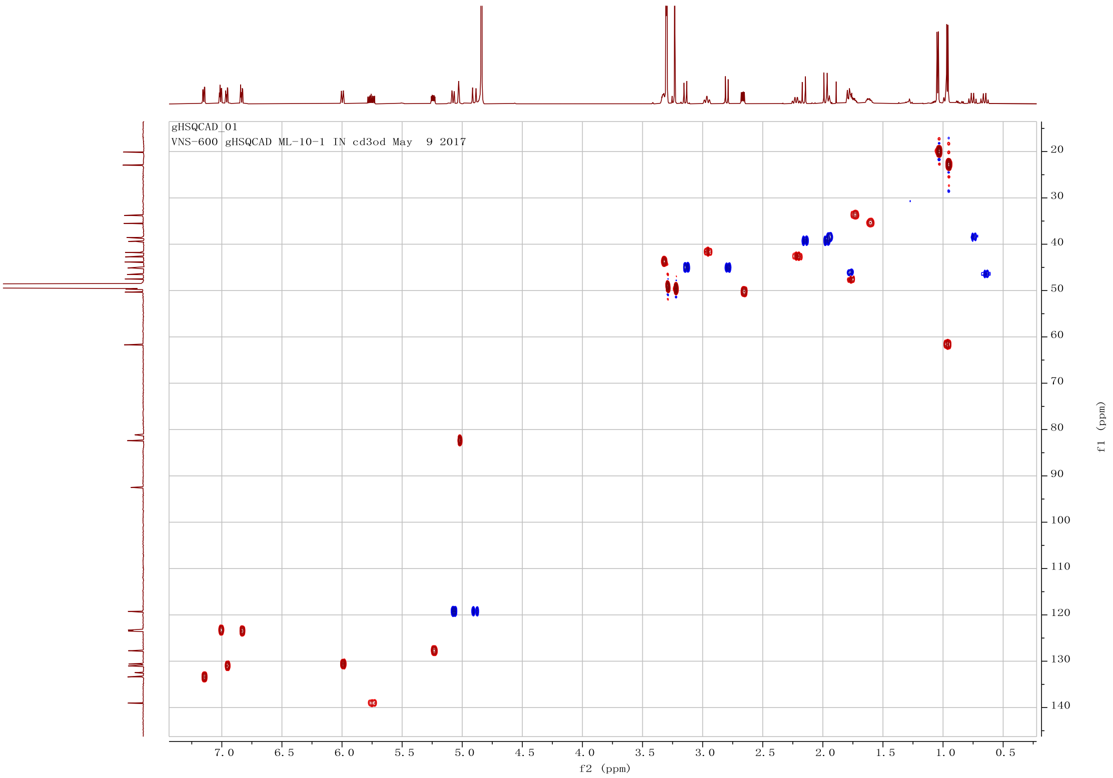

Enlarged figure

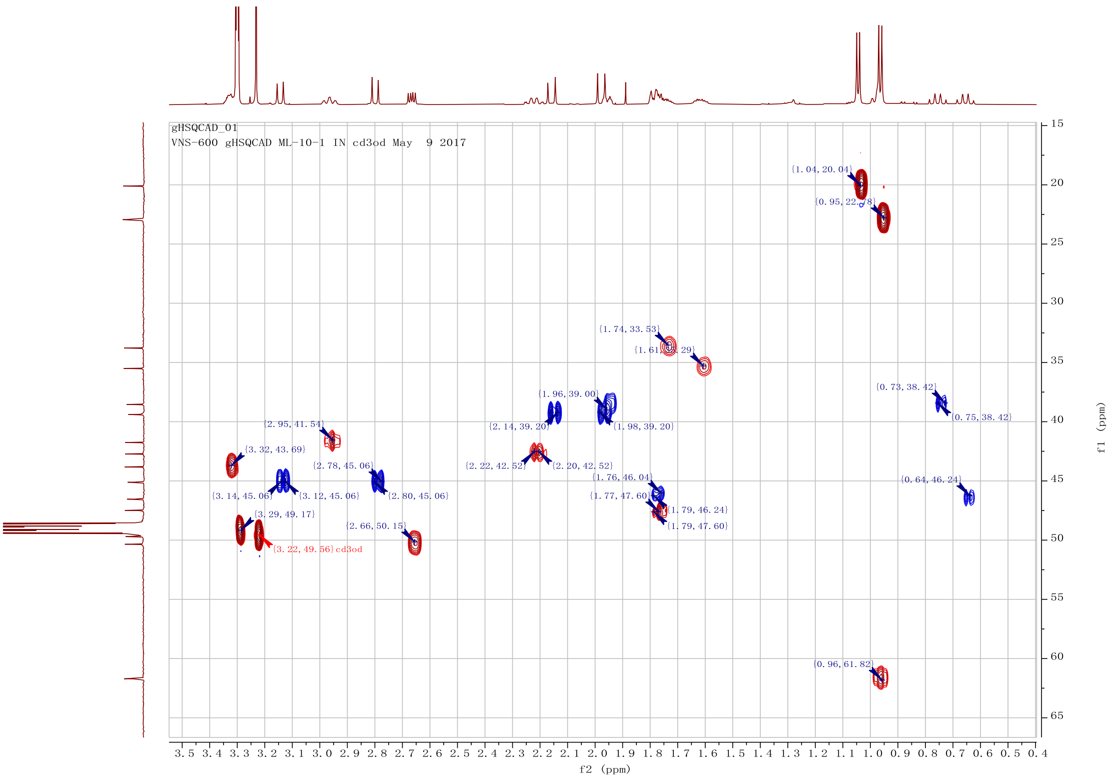

Enlarged figure

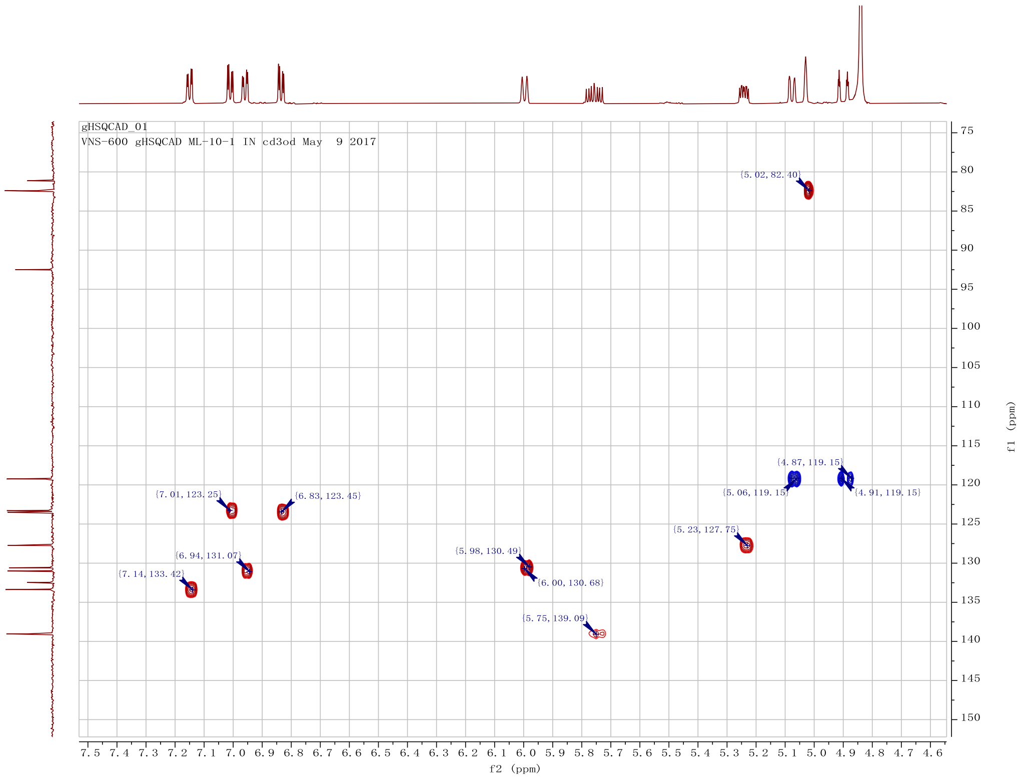

Figure S24 HMBC spectrum of xenoacremone F (**3**) in CD_3OD

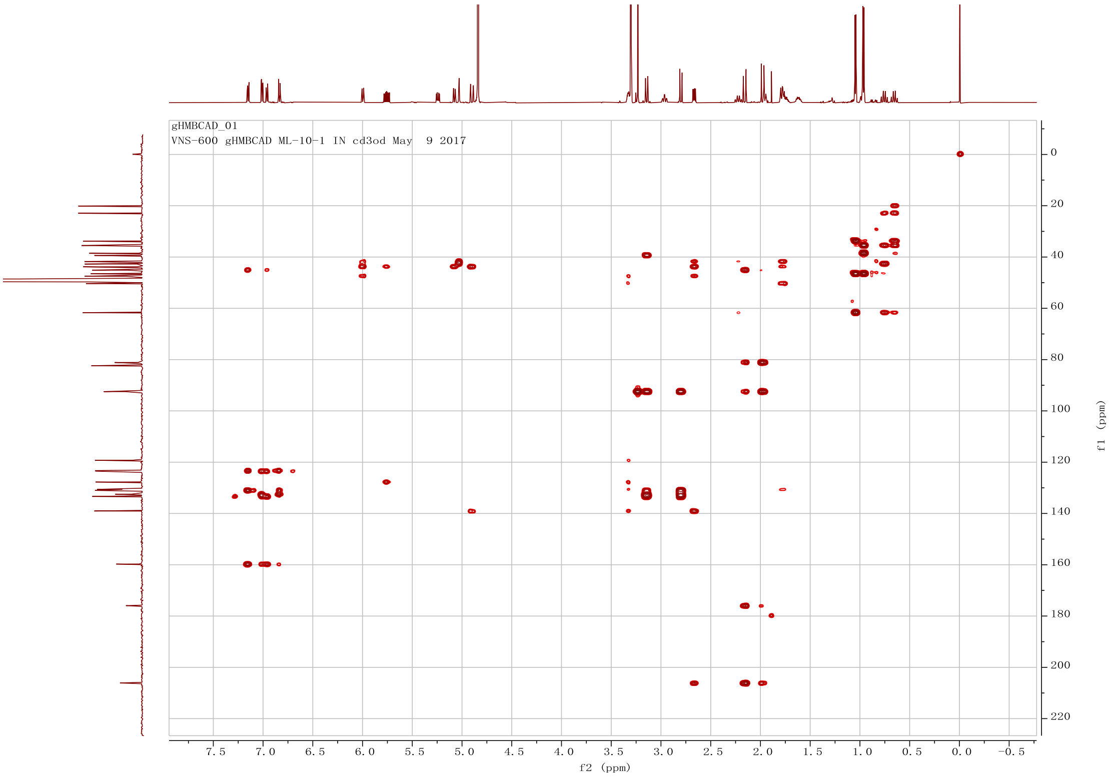

Enlarged figure

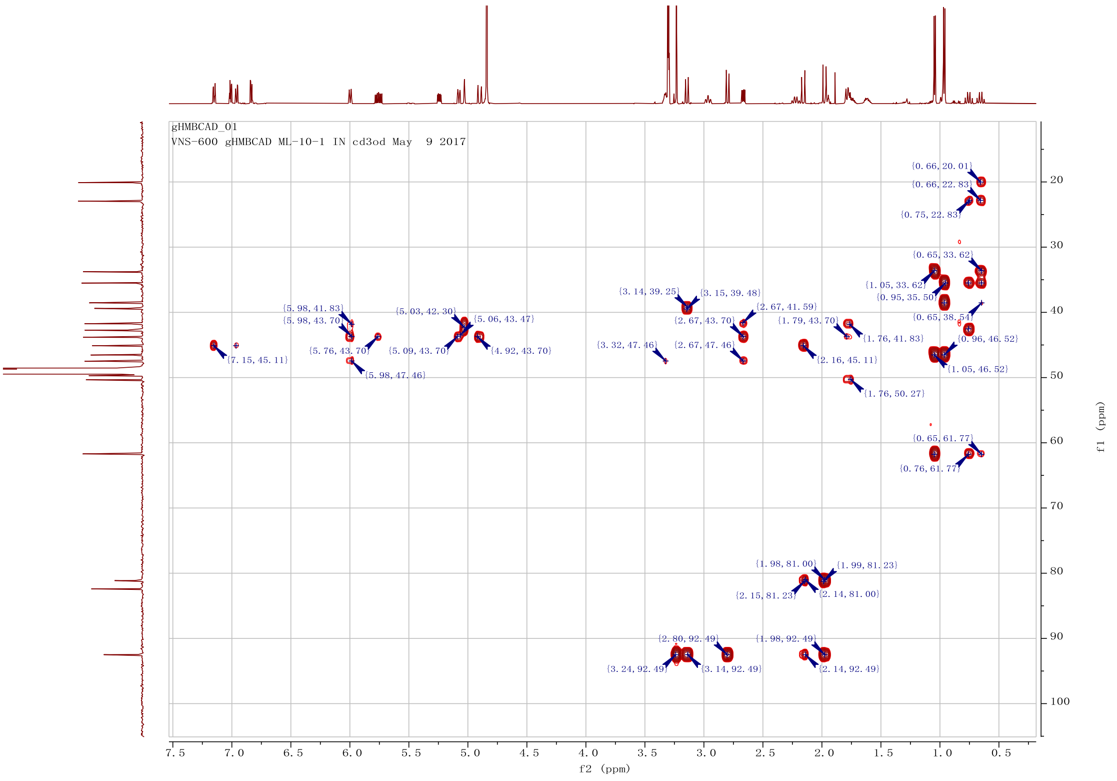

Enlarged figure

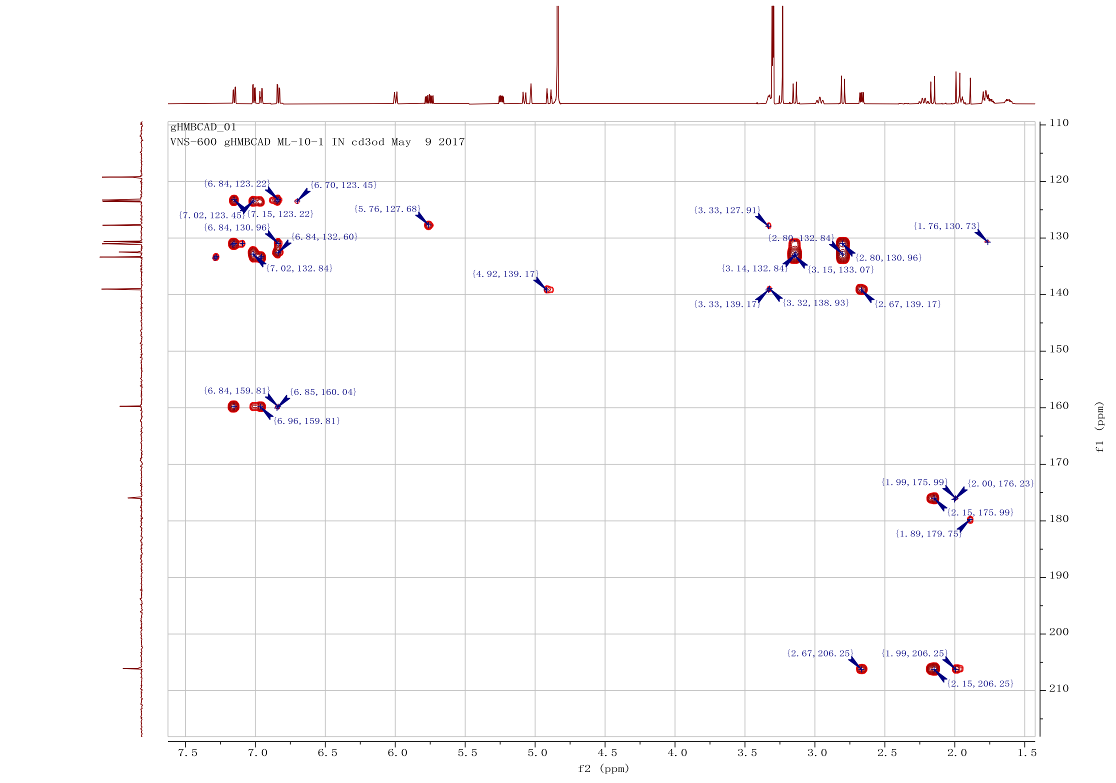

Figure S25 ^1H-^1H COSY spectrum of xenoacremone F (**3**) in CD_3OD

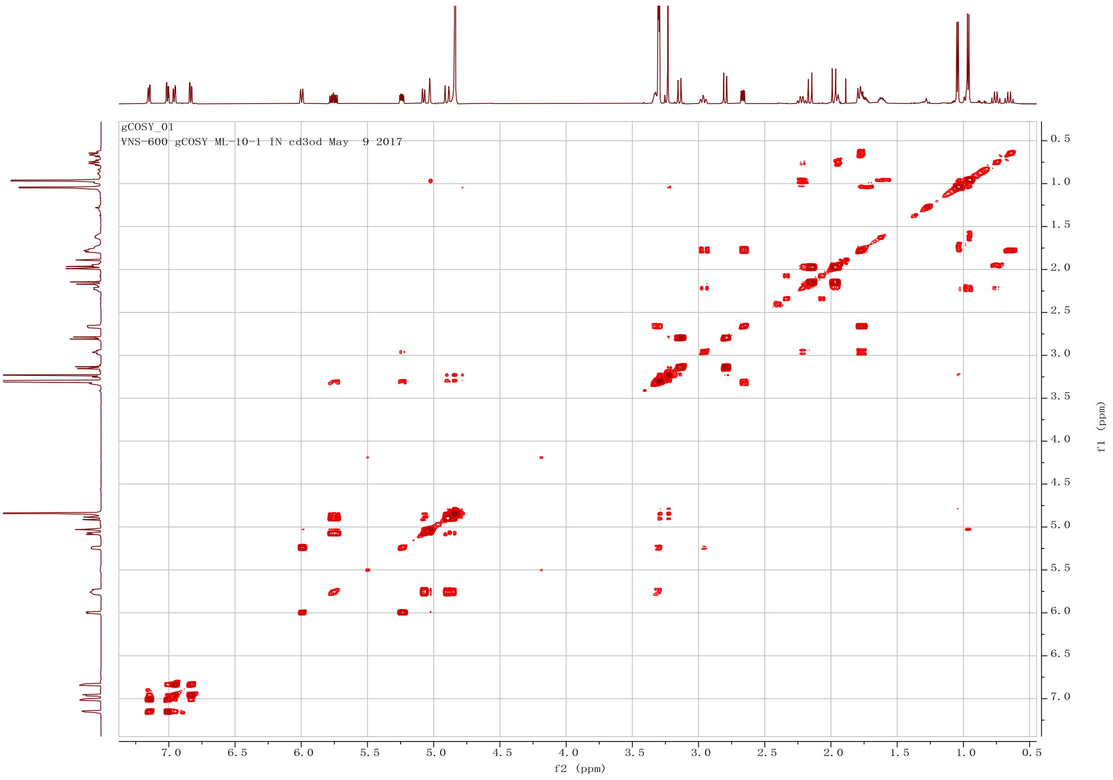

Enlarged figure

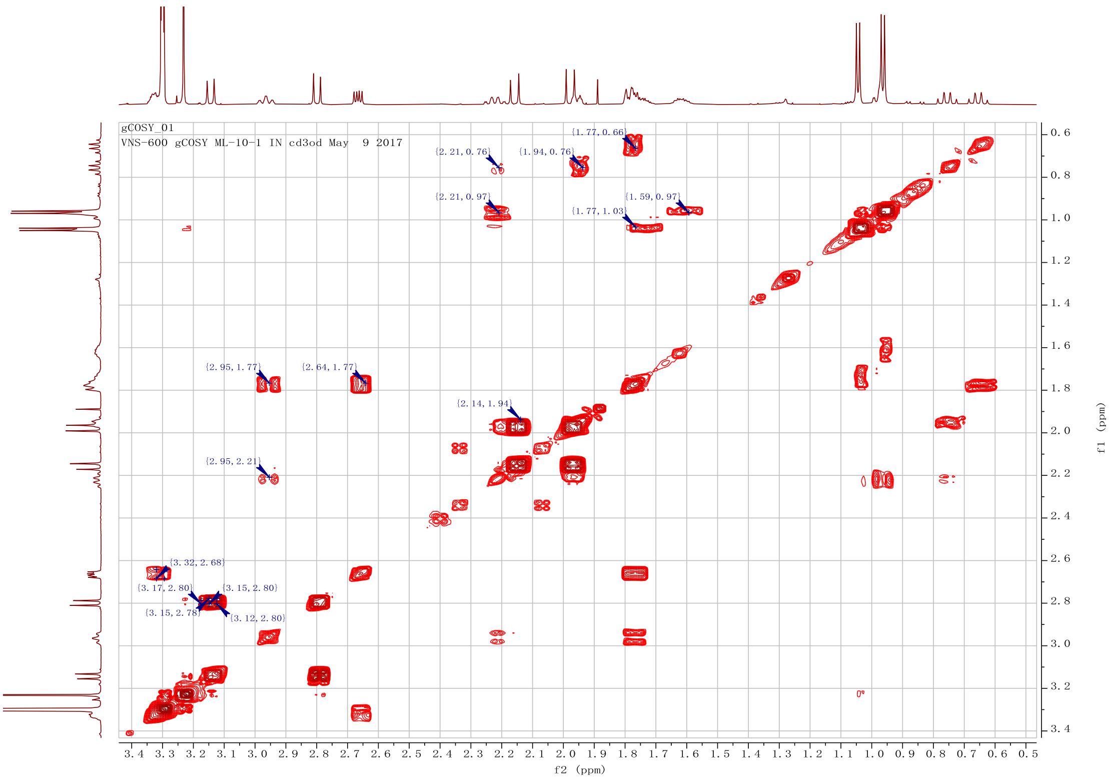

Enlarged figure

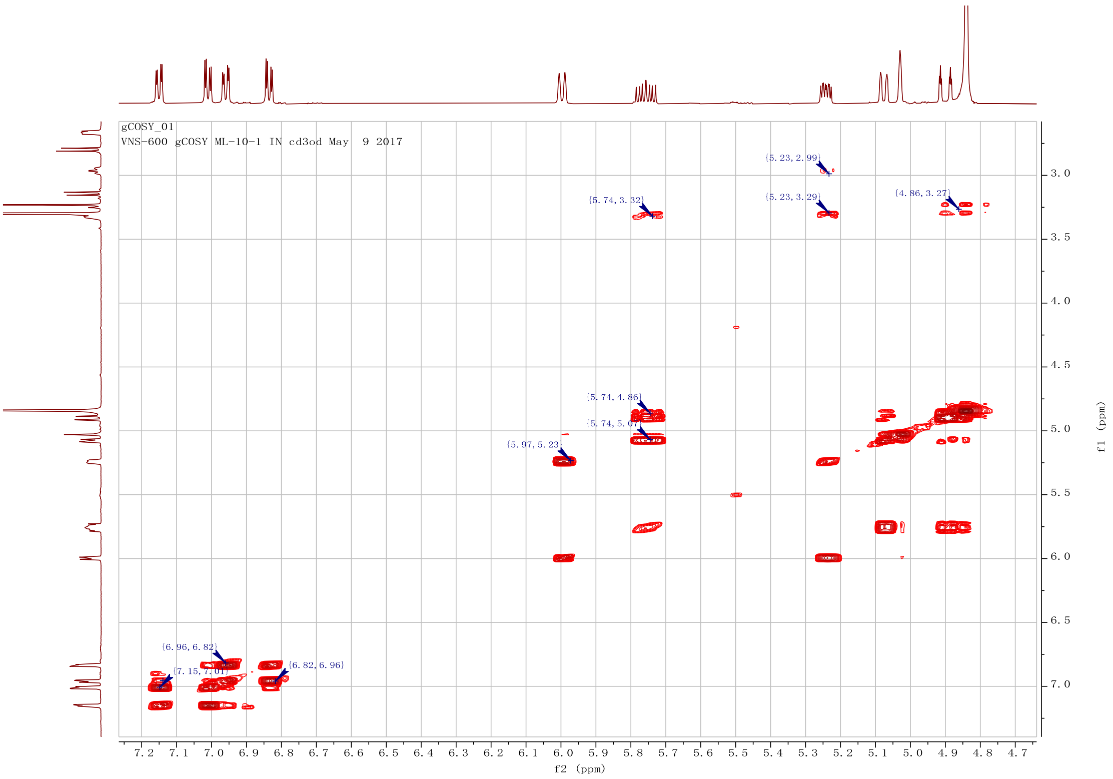

Figure S26 ROESY spectrum of xenoacremone F (**3**) in CD3OD

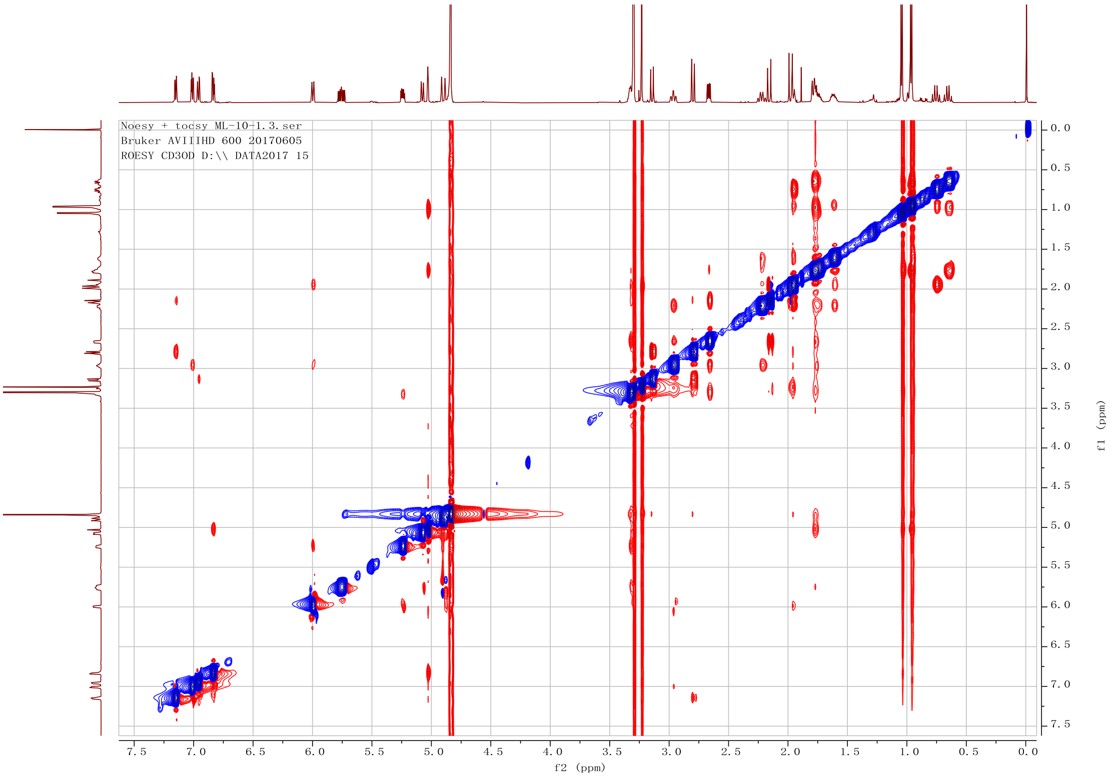

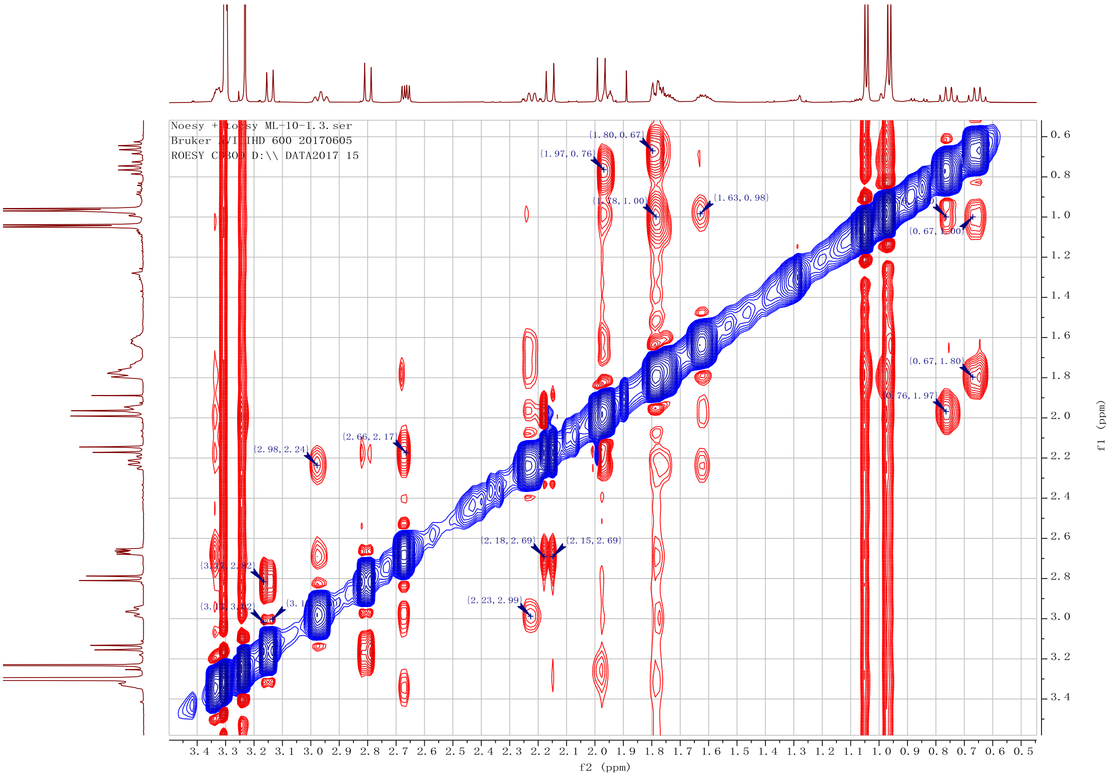
f2 (ppm)
f1 (ppm)

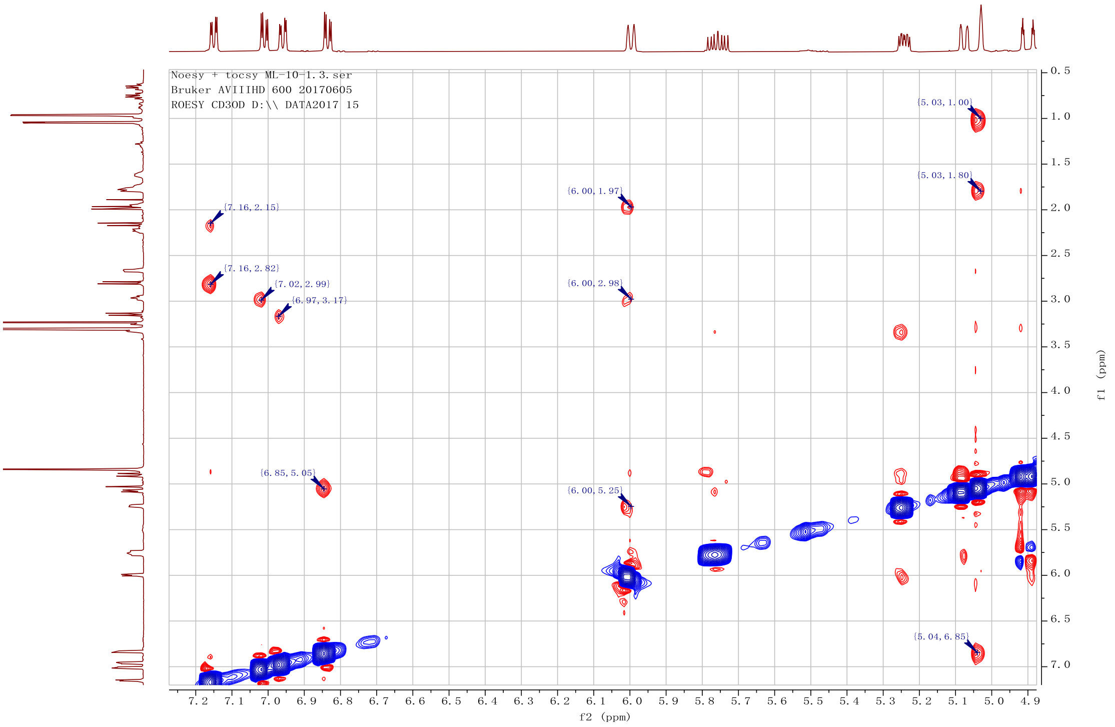
Noesy + tocsy ML-10-1.3.ser
Bruker AVIIIHD 600 20170605
ROESY CD3OD D:\\ DATA2017 15
f2 (ppm)
f1 (ppm)

Figure S27 ECD spectrum of xenoacremone F (**3**) in MeOH

TDDFT theory, ωB97XD functional and TZVP level of theory, **3** has 3 conformations. Cam-B3LYP functional and TZVP level calculate 70 excited states, the solvent is methanol, σ=0.40eV, the result is shown in the figure.

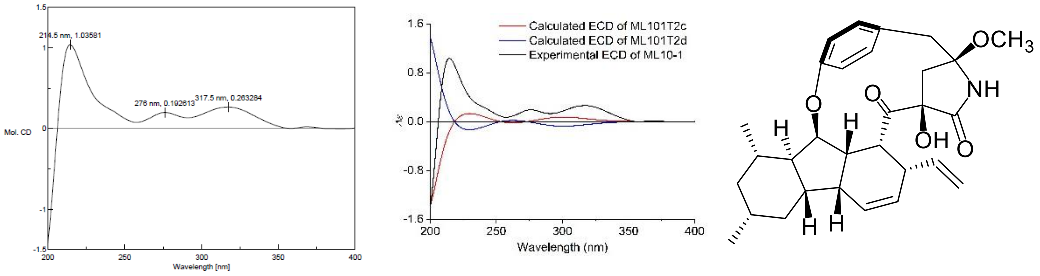

Figure S28 HR ESIMS spectrum of xenoacremone F (**3**)

Composition	i-FIT Confidence (%)	m/z RMS (PPM)	Intensity RMS (%)	Predicted m/z	m/z error (PPM)	m/z error (mDa)	DBE
C30H37NO5	82.786885	4.442089	3.735090	492.274450	4.397280	2.160249	13.000000

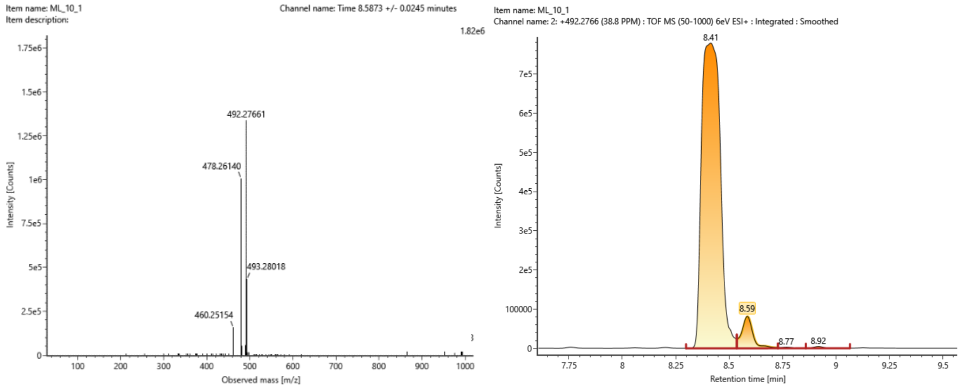

Figure S29 ^{1}H NMR spectrum of xenoacremone G (**4**) in CD_3OD

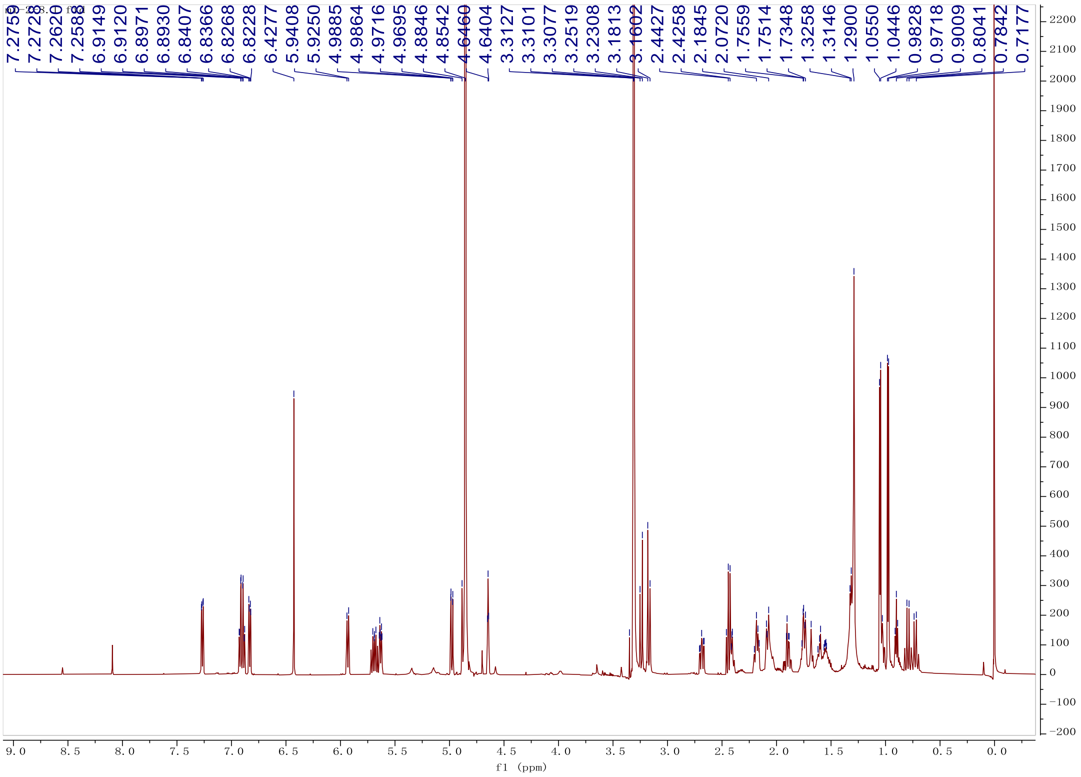

Figure S30 ^{13}C NMR spectrum of xenoacremone G (**4**) in CD_3OD

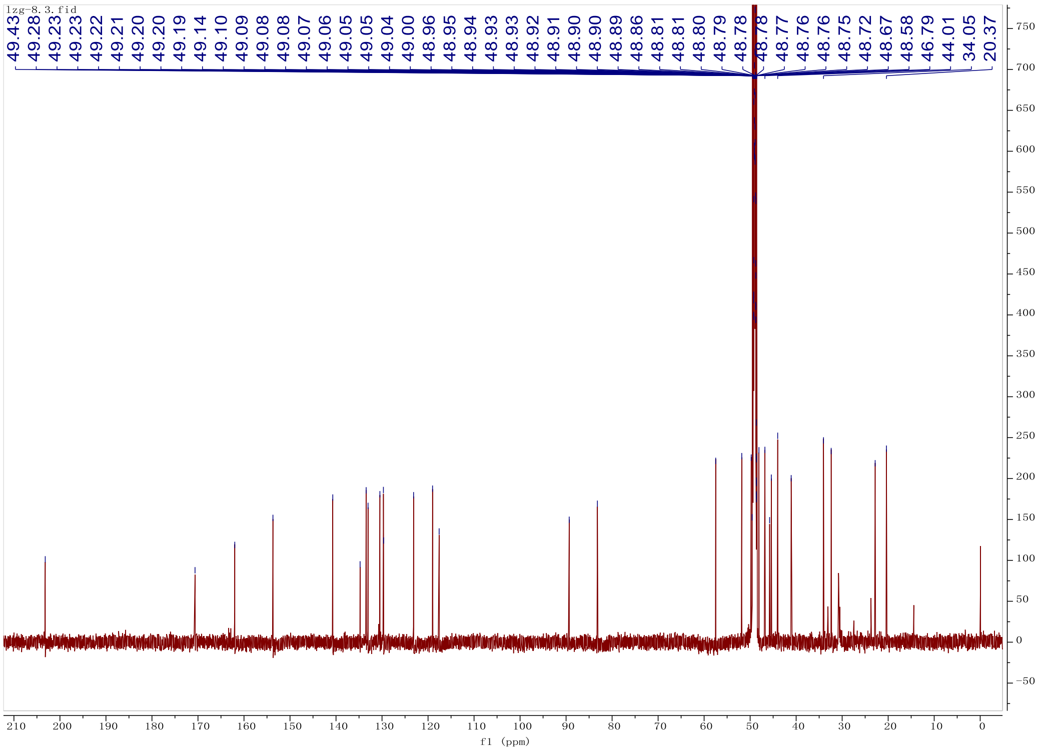

Figure S31 HSQC spectrum of xenoacremone G (**4**) in CD_3OD

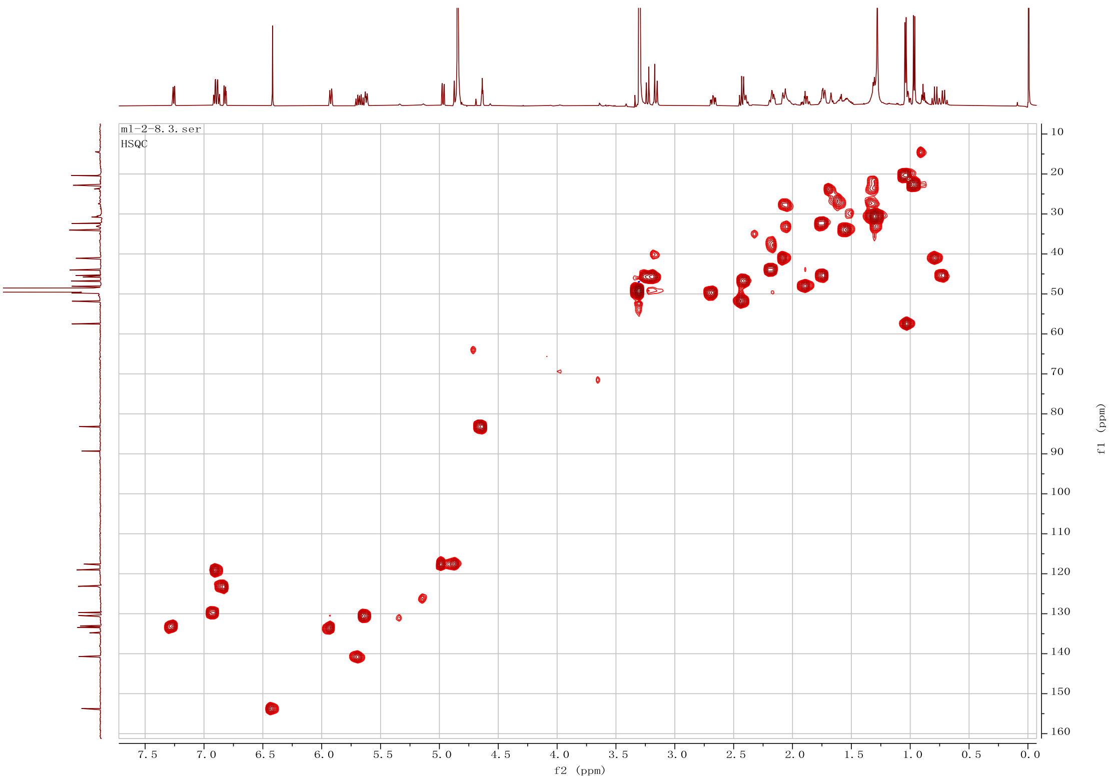

Enlarged figure

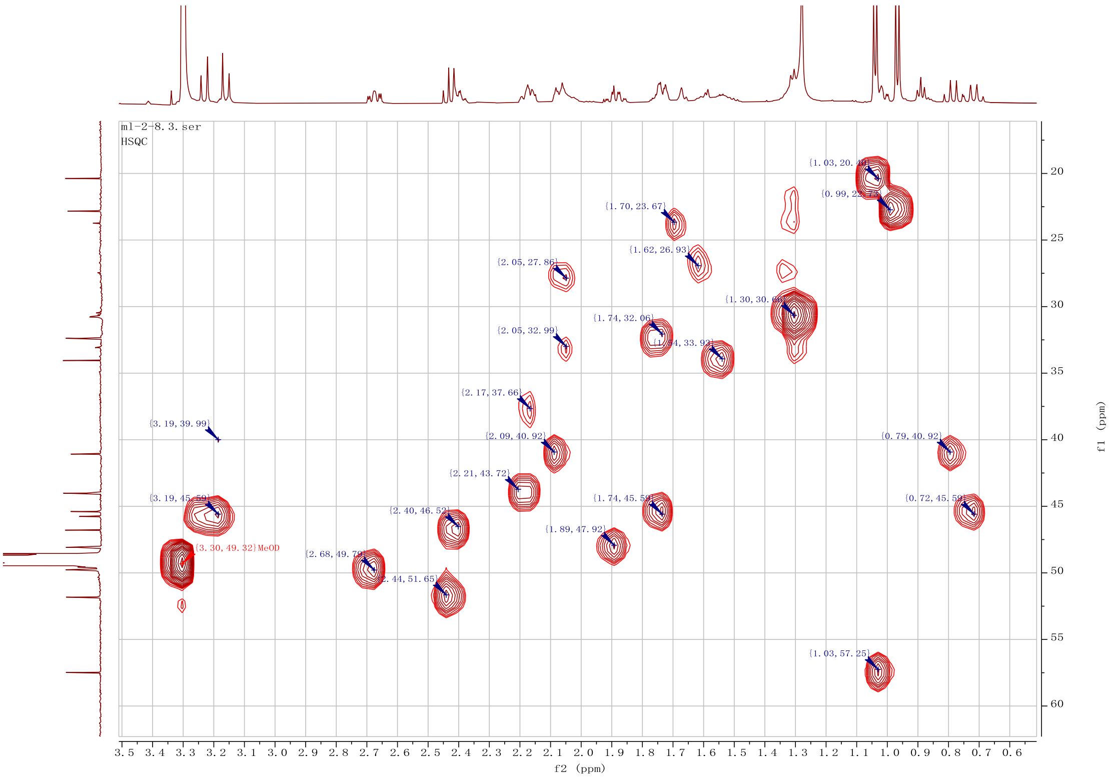

Enlarged figure

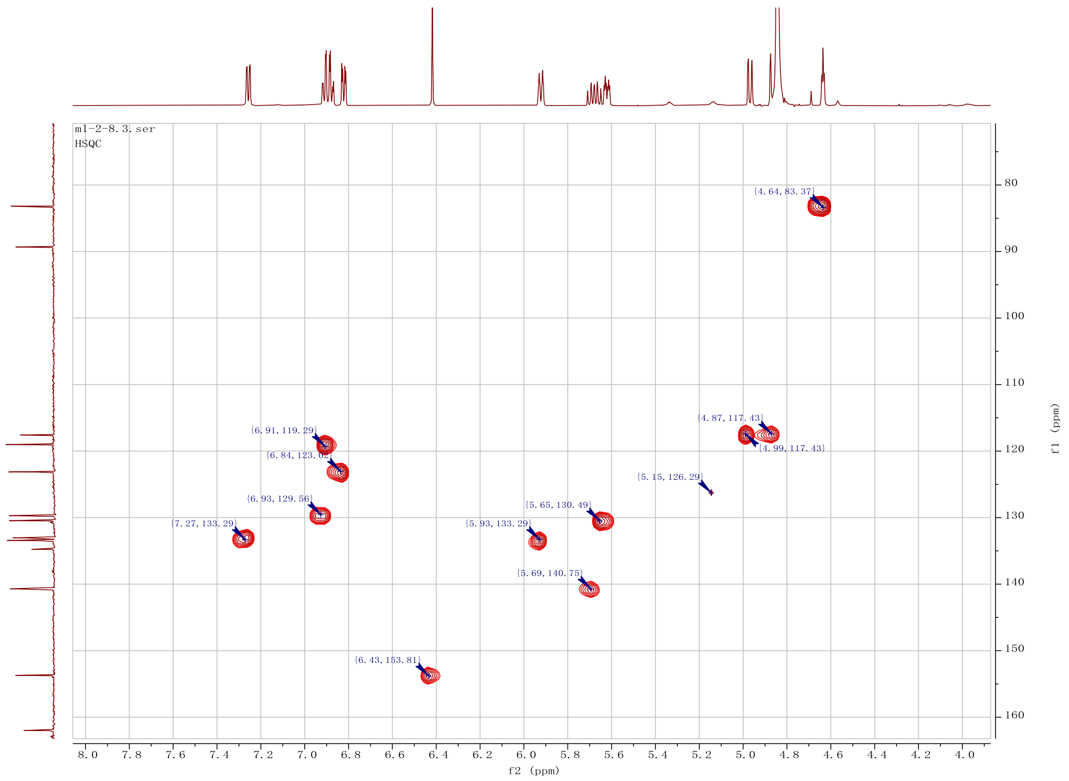

Figure S32 HMBC spectrum of xenoacremone G (**4**) in CD_3OD

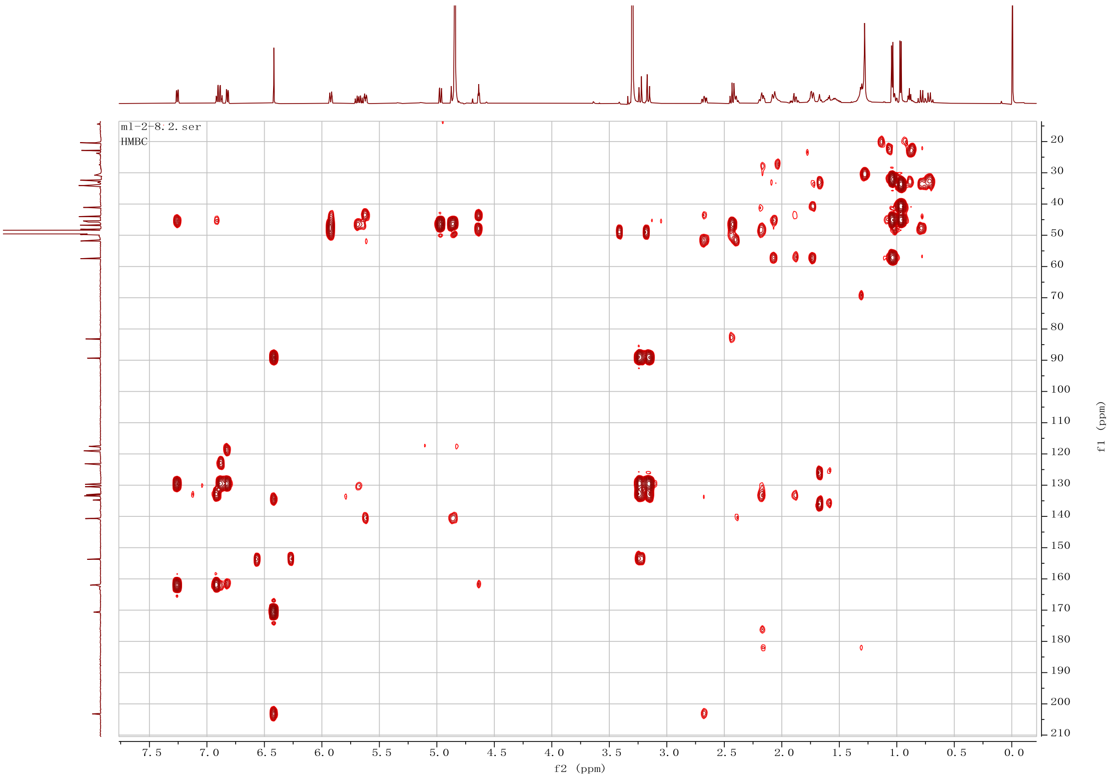

Enlarged figure

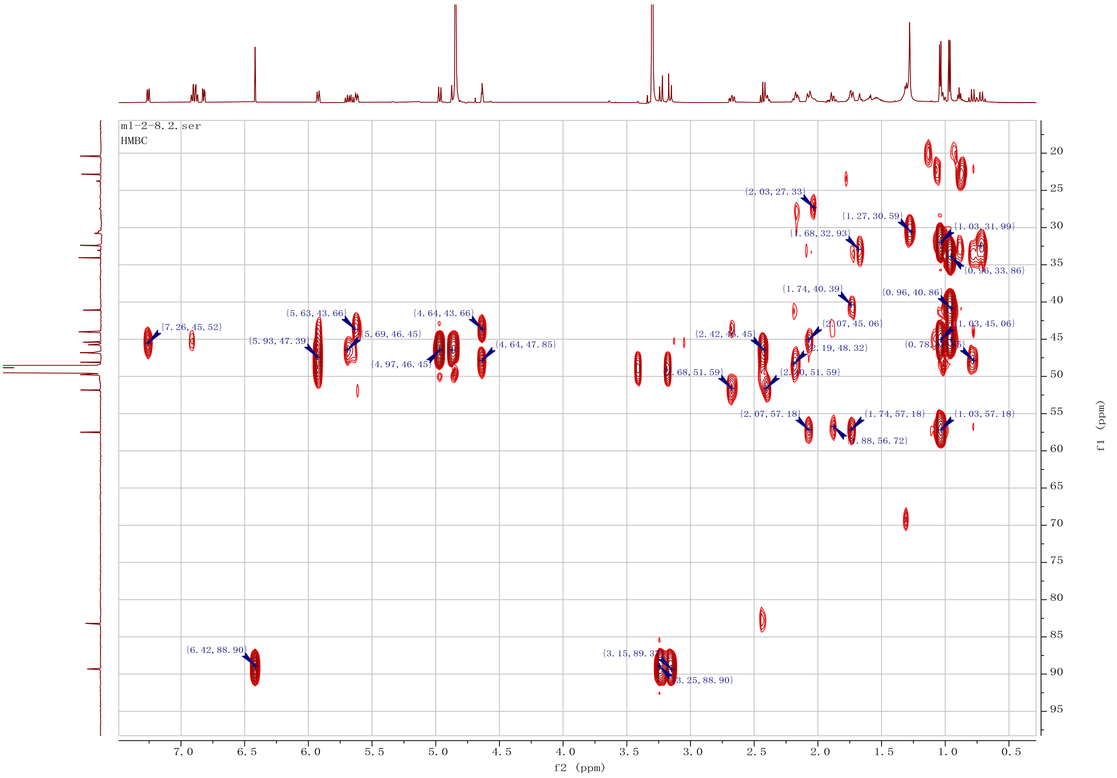

Enlarged figure

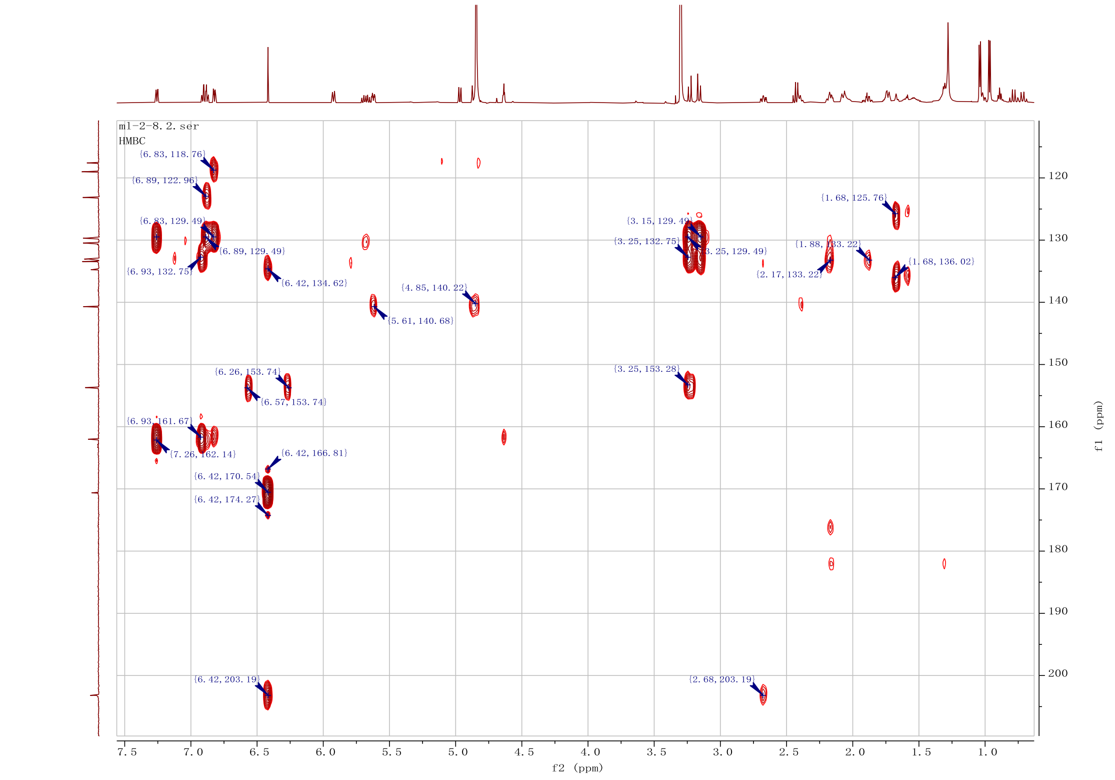

Figure S33 ^1H-^1H COSY spectrum of xenoacremone G (**4**) in CD_3OD

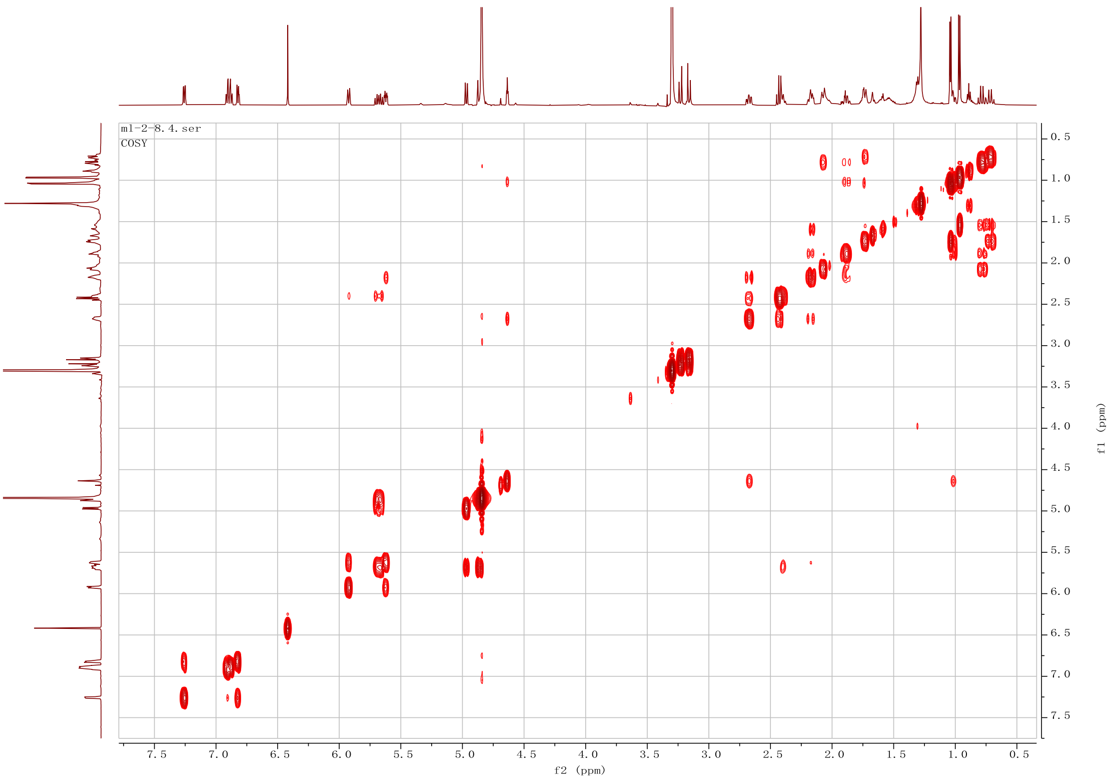

Enlarged figure

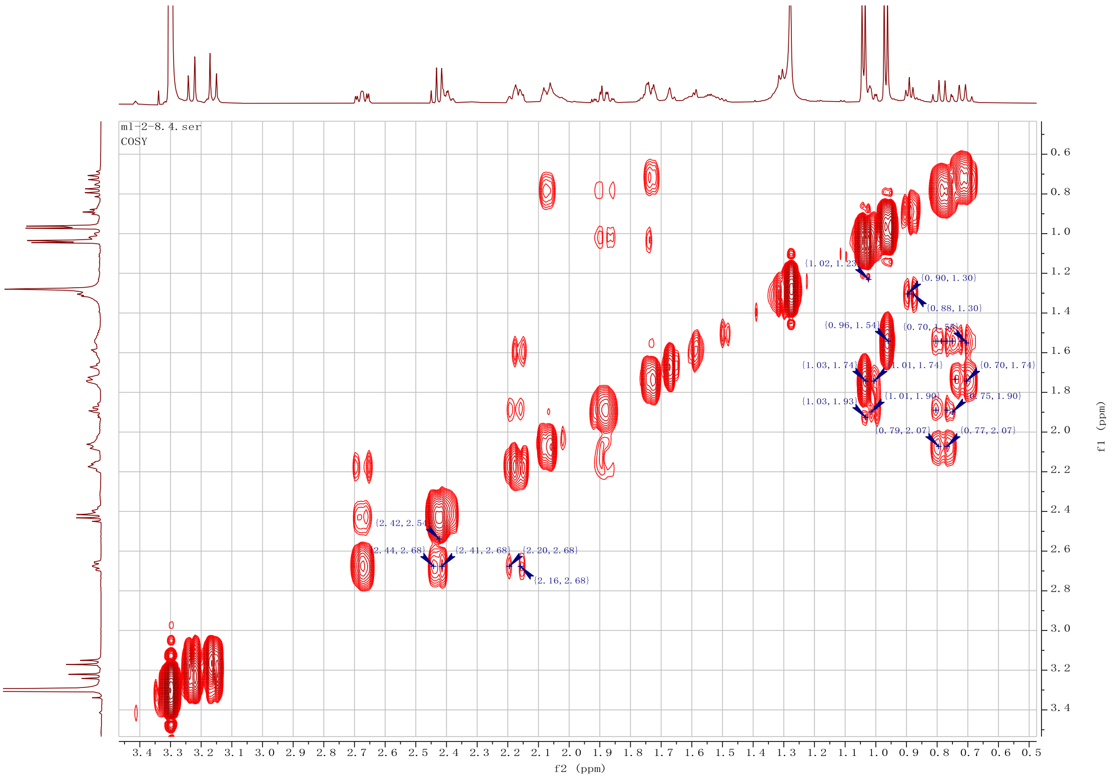

Enlarged figure

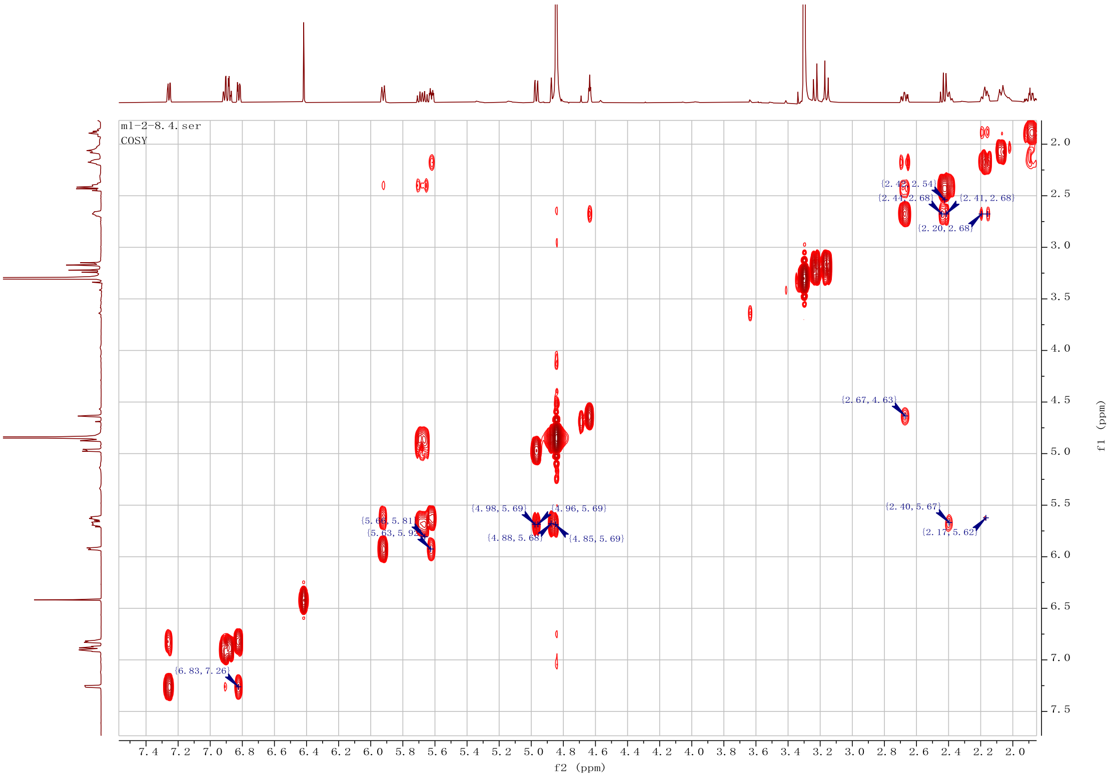

Figure S34 NOESY spectrum of xenoacremone G (**4**) in CD_3OD

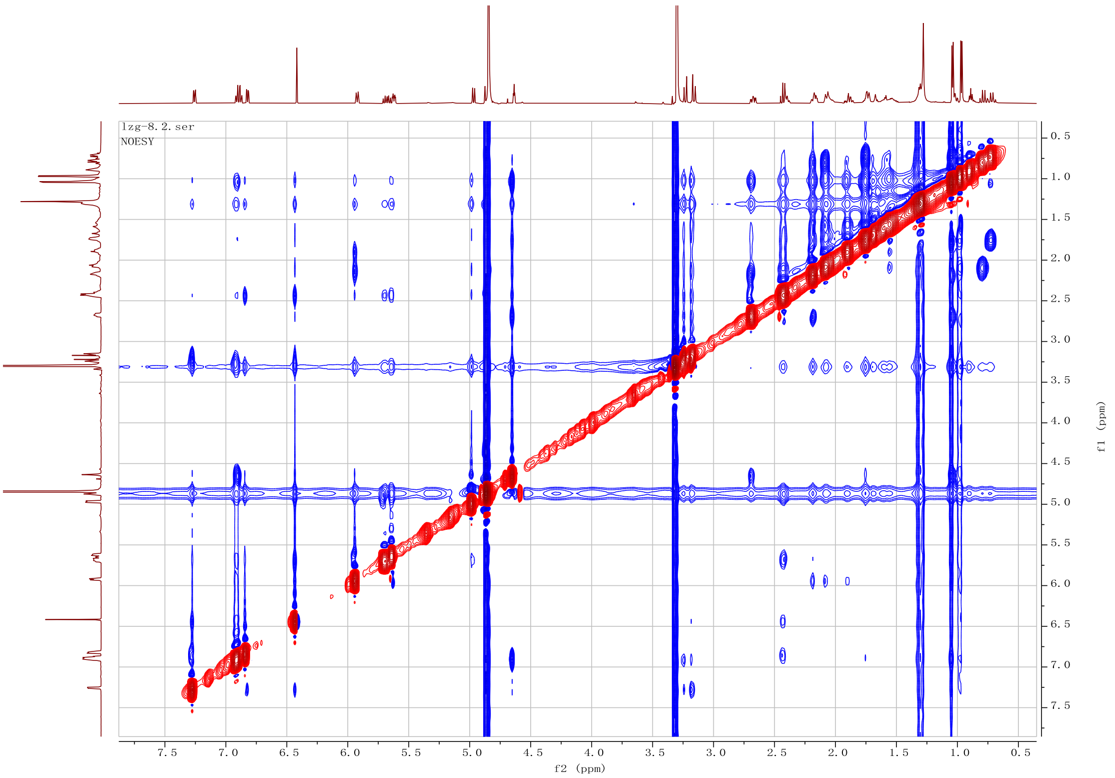

Enlarged figure

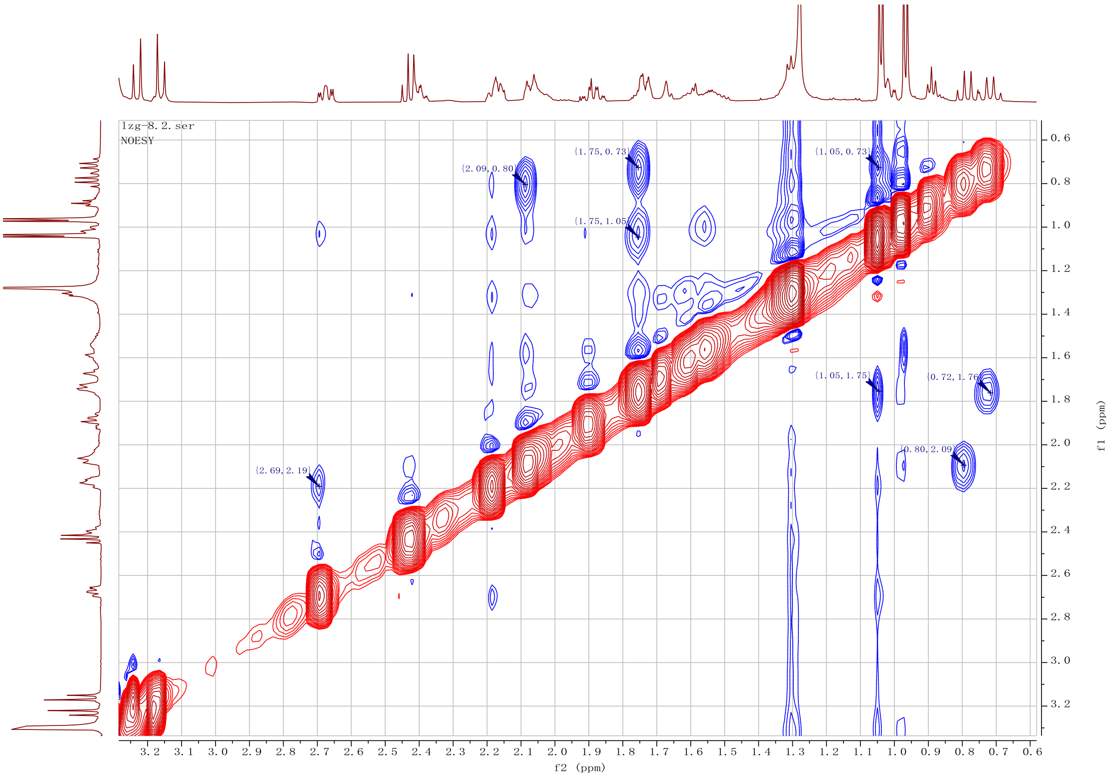

Enlarged figure

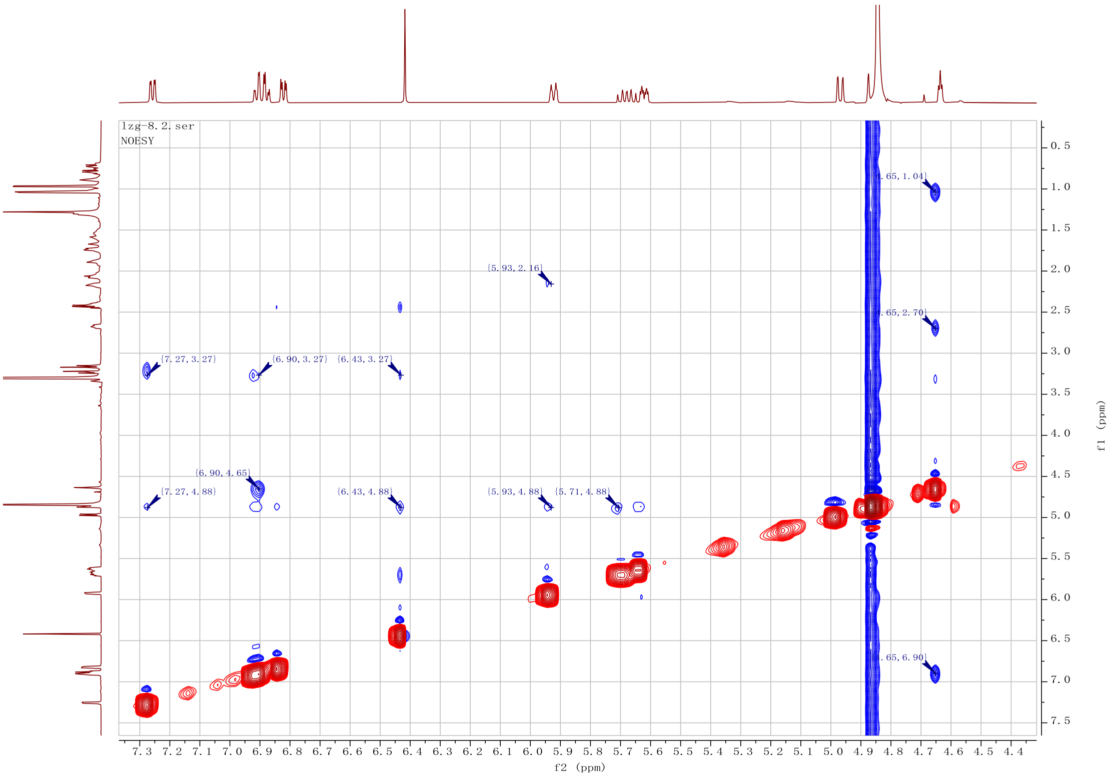

Figure S35 ECD spectrum of xenoacremone G (**4**) (in MeOH)

TDDFT theory, ωB97XD functional and TZVP level of theory, **4** has 4 conformations. Cam-B3LYP functional and TZVP level calculate 70 excited states, the solvent is methanol, σ=0.40eV, the result is shown in the figure.

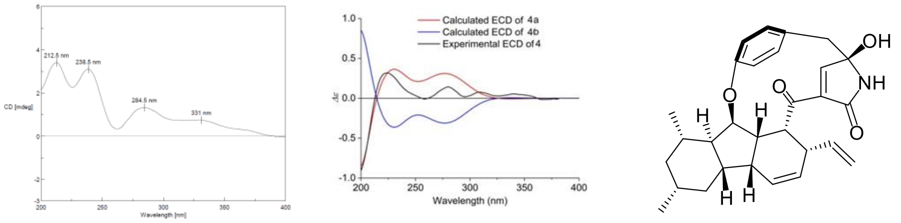

Figure S36 HRESI-MS spectrum of xenoacremone G (**4**)

Composition	i-FIT Confidence (%)	m/z RMS (PPM)	Intensity RMS (%)	Predicted m/z	m/z error (PPM)	m/z error (mDa)	DBE
C29H33NO4	99.991502	0.743252	3.187766	460.248235	0.838336	0.384999	14.000000

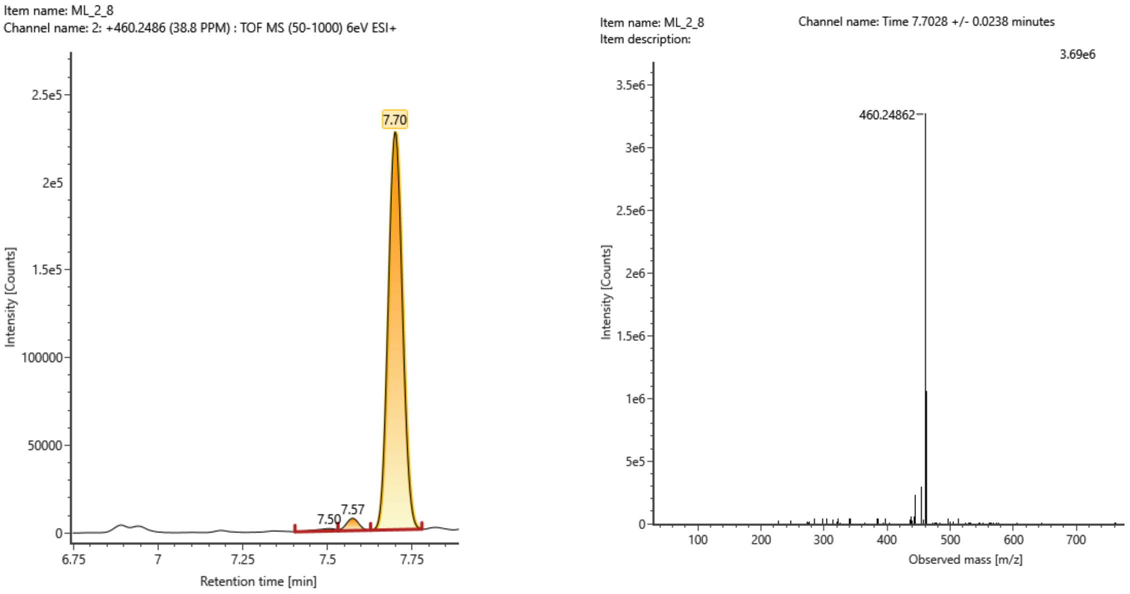

Figure S37 ^{1}H NMR spectrum of xenoacremone H (**5**) in $CDCl_3$

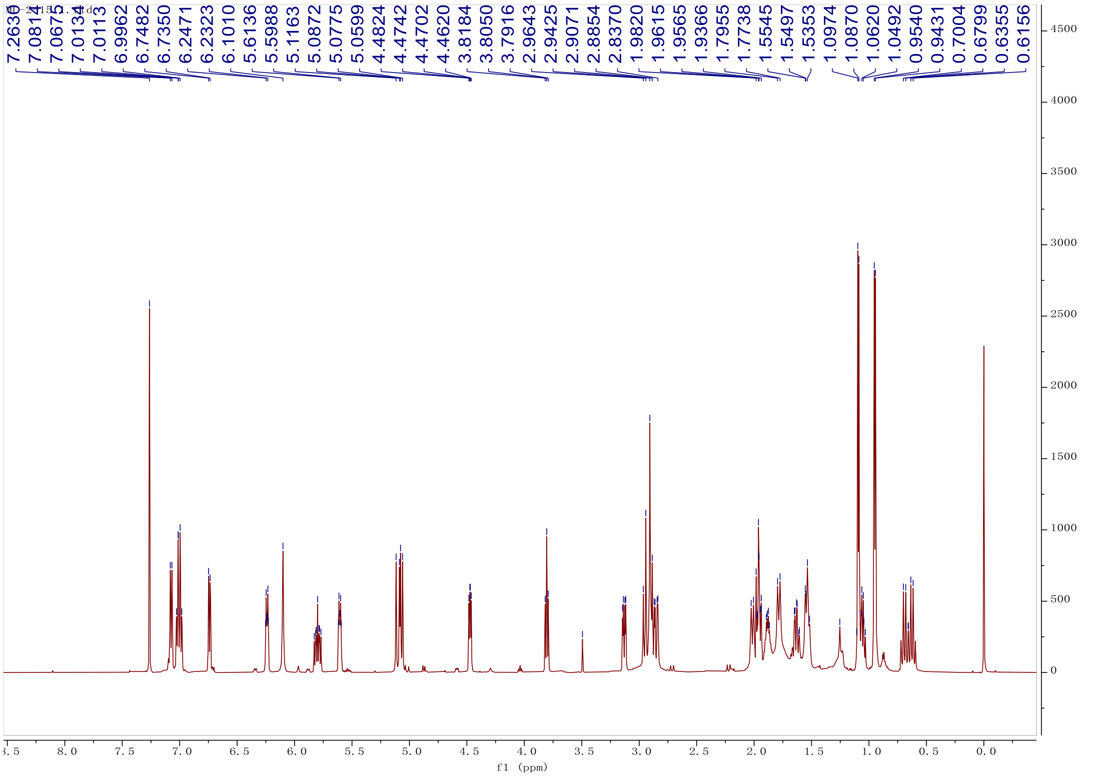

Figure S38 ^{13}C NMR spectrum of xenoacremone H (**5**) in $CDCl_3$

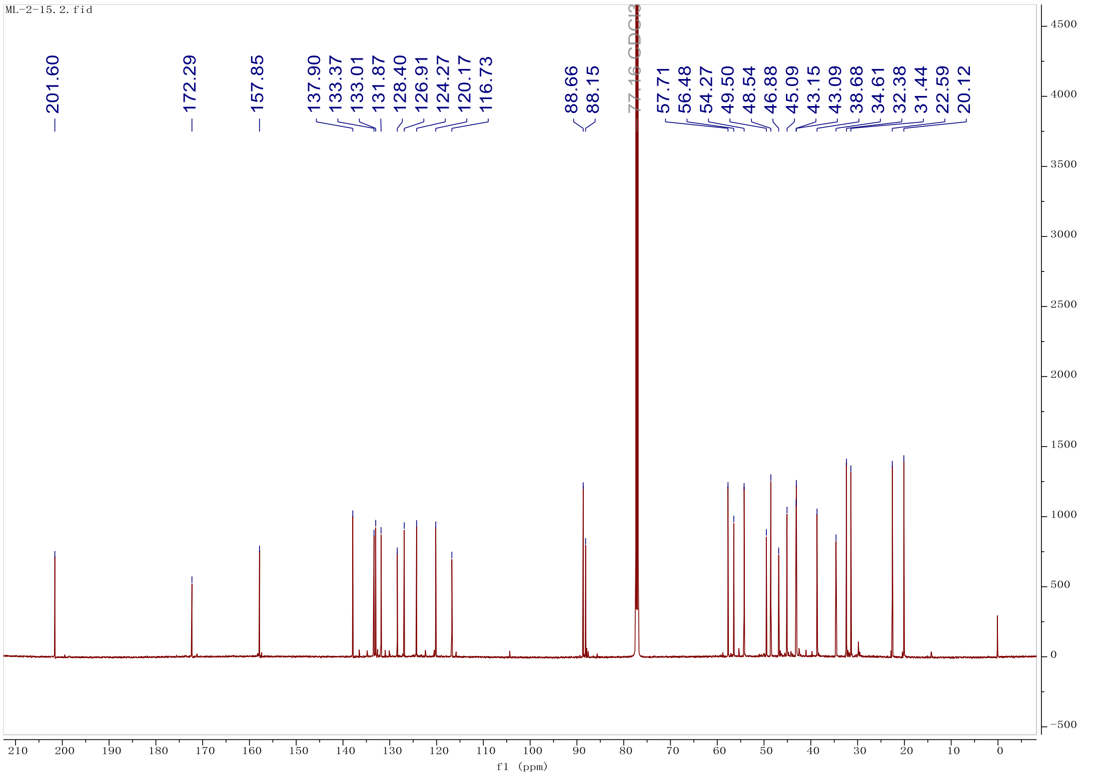

Figure S39 HSQC spectrum of xenoacremone H (**5**) in $CDCl_3$

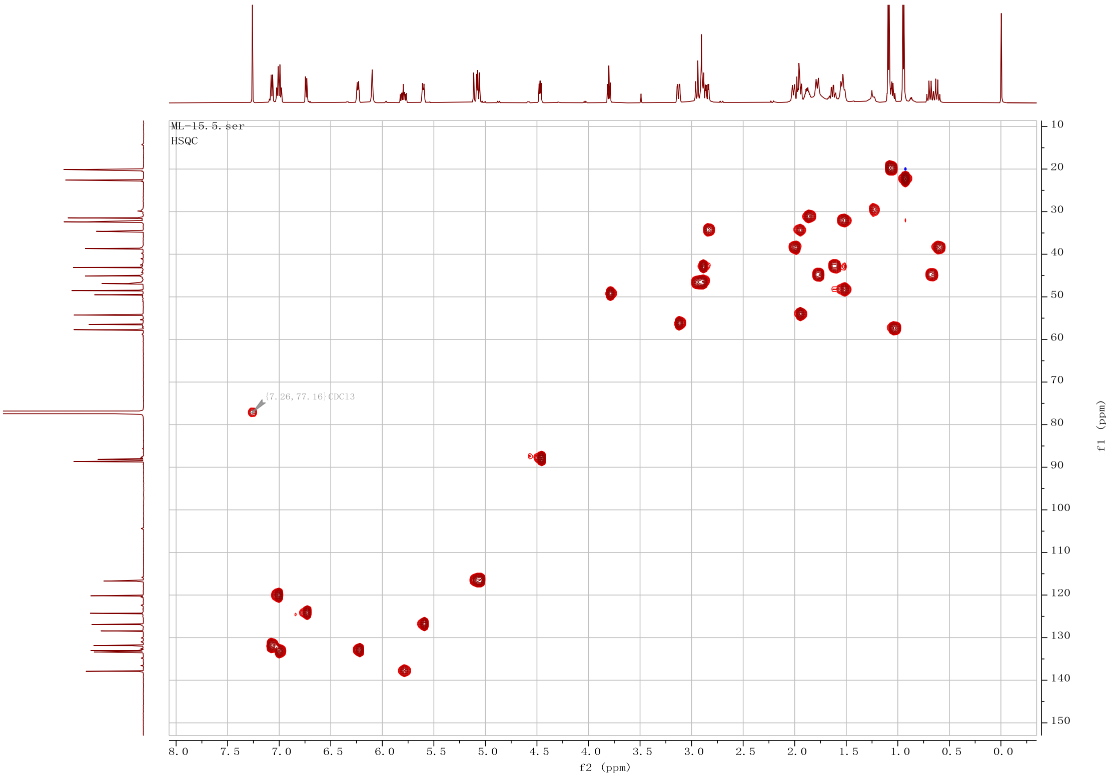

Enlarged figure

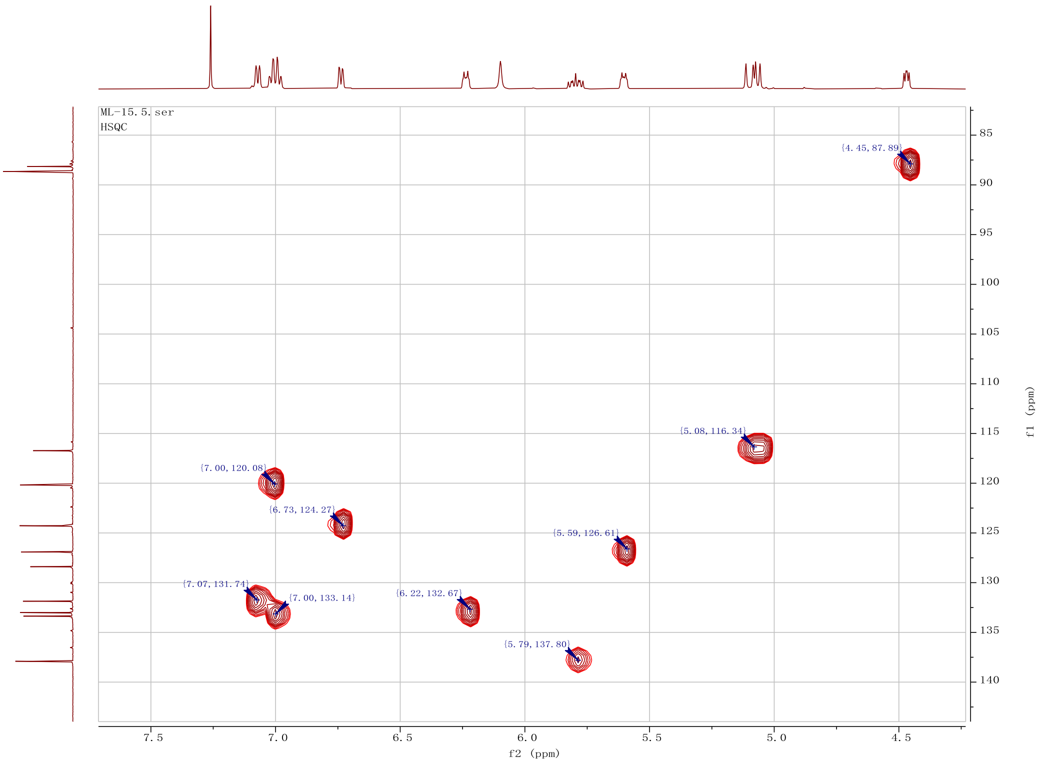

Enlarged figure

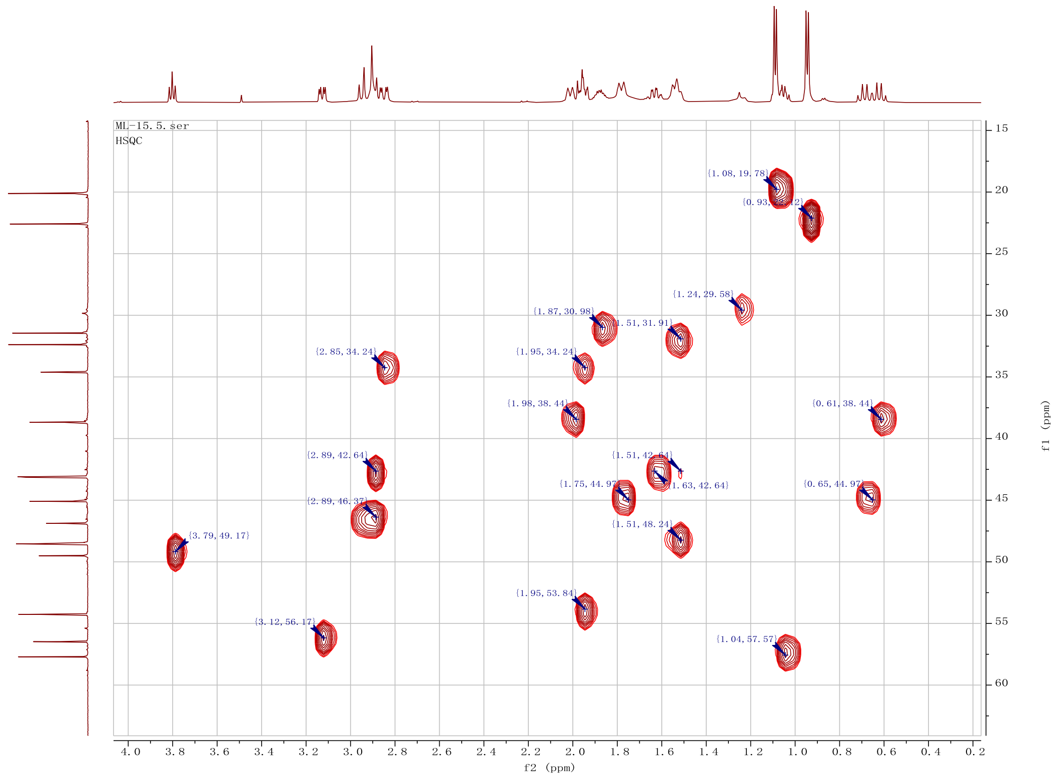

Figure S40 HMBC spectrum of xenoacremone H (**5**) in $CDCl_3$

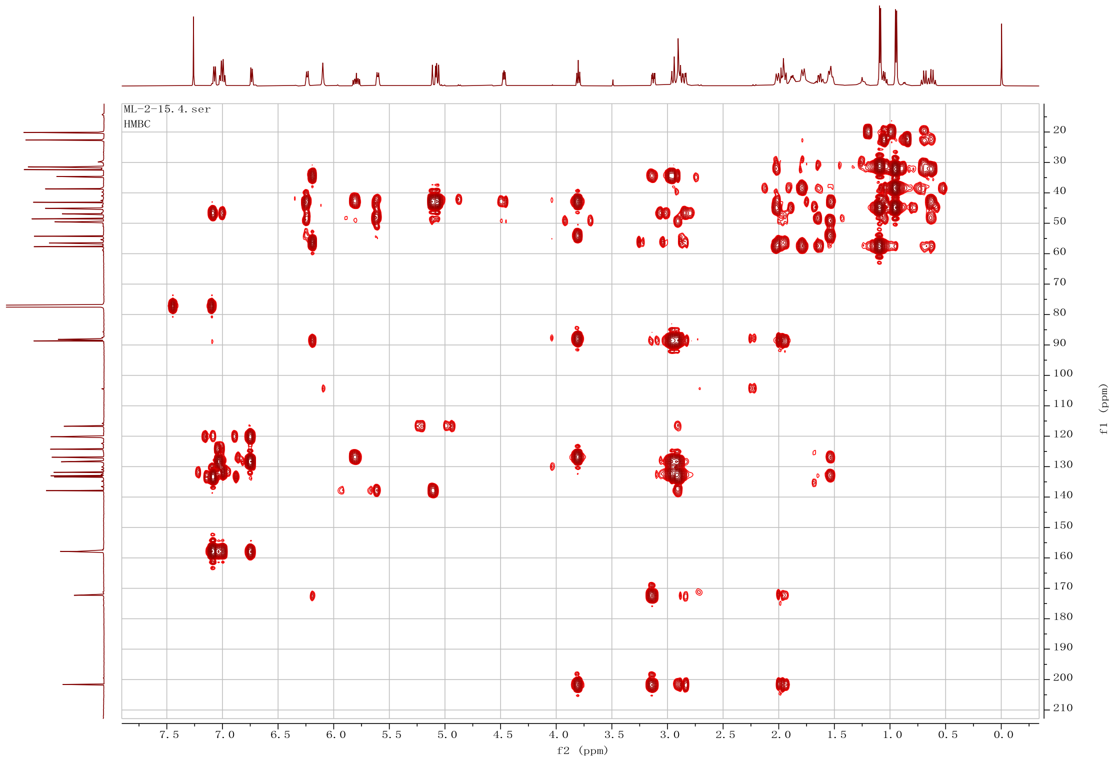

Enlarged figure

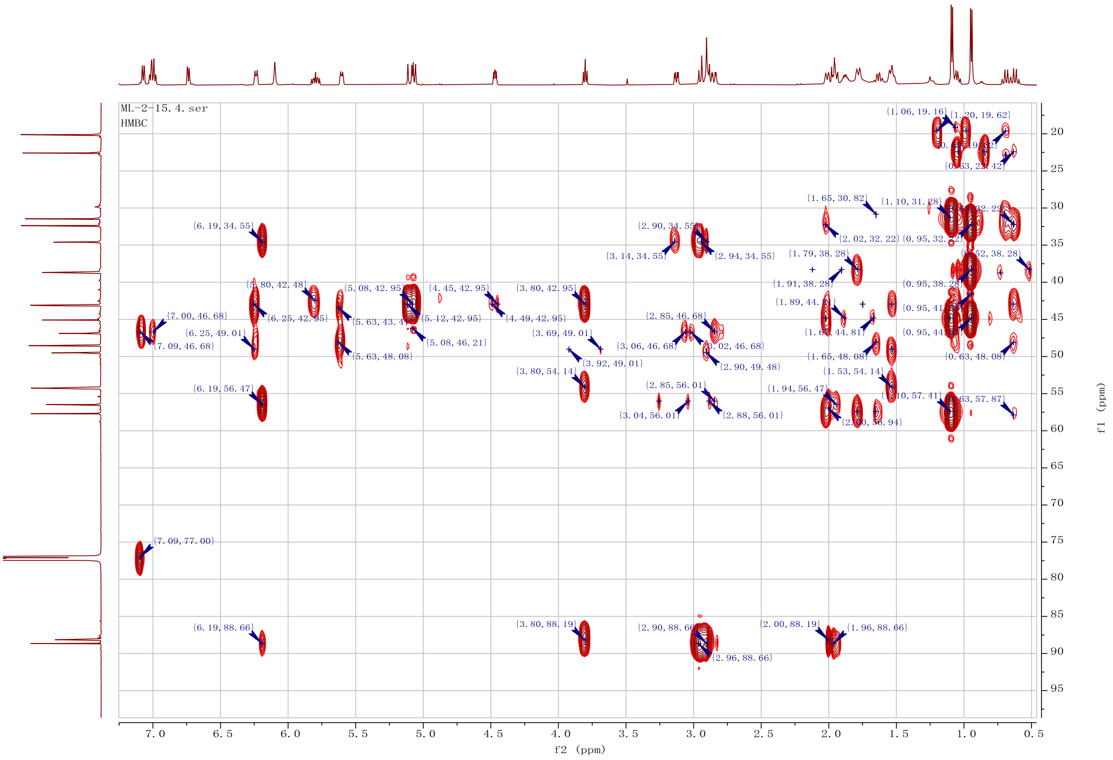

Enlarged figure

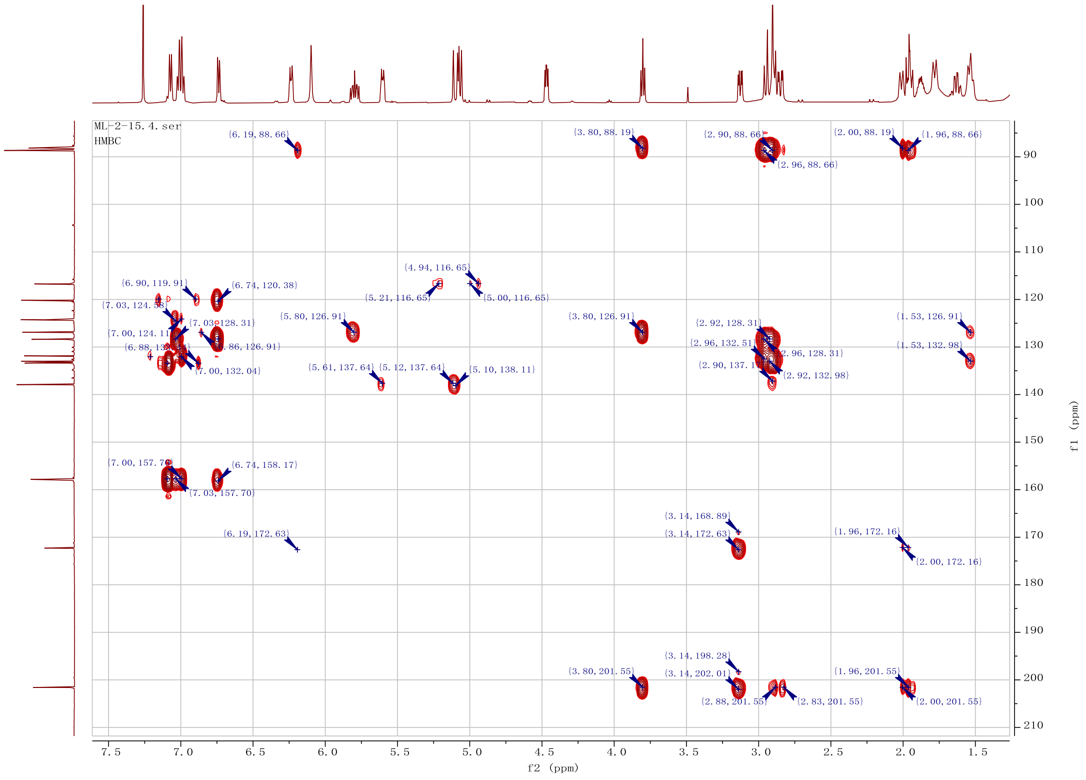

Figure S41 ^1H-^1H COSY spectrum of xenoacremone H (**5**) in $CDCl_3$

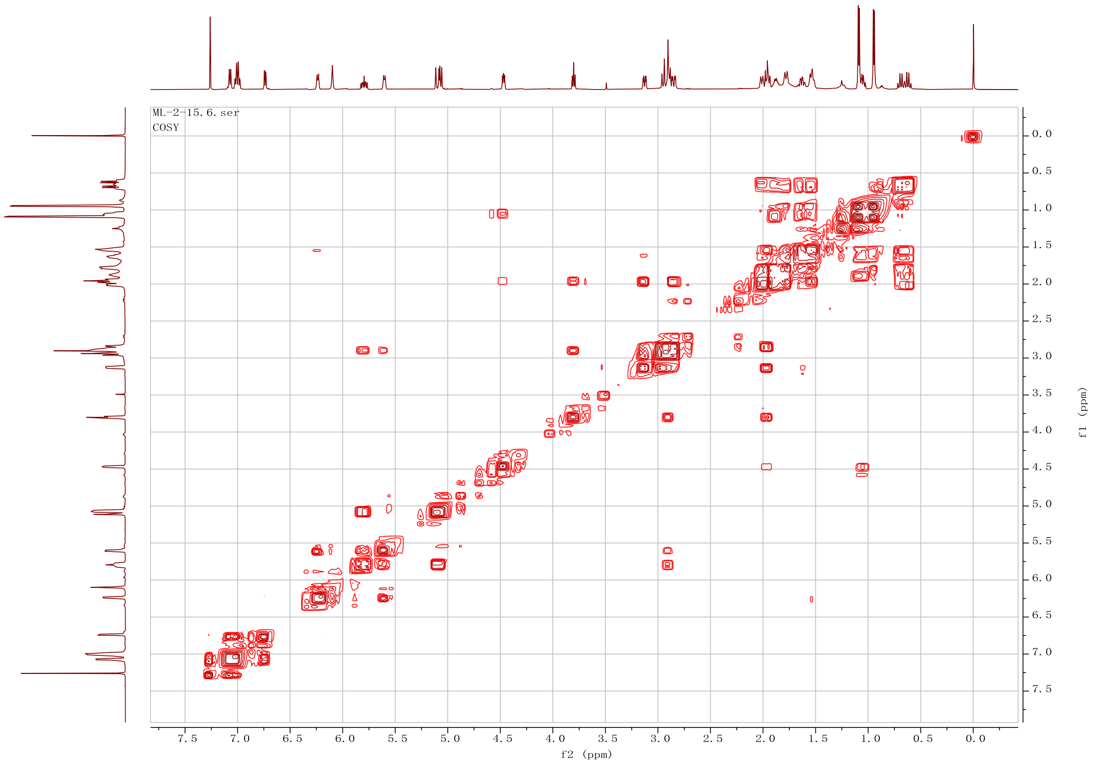

Enlarged figure

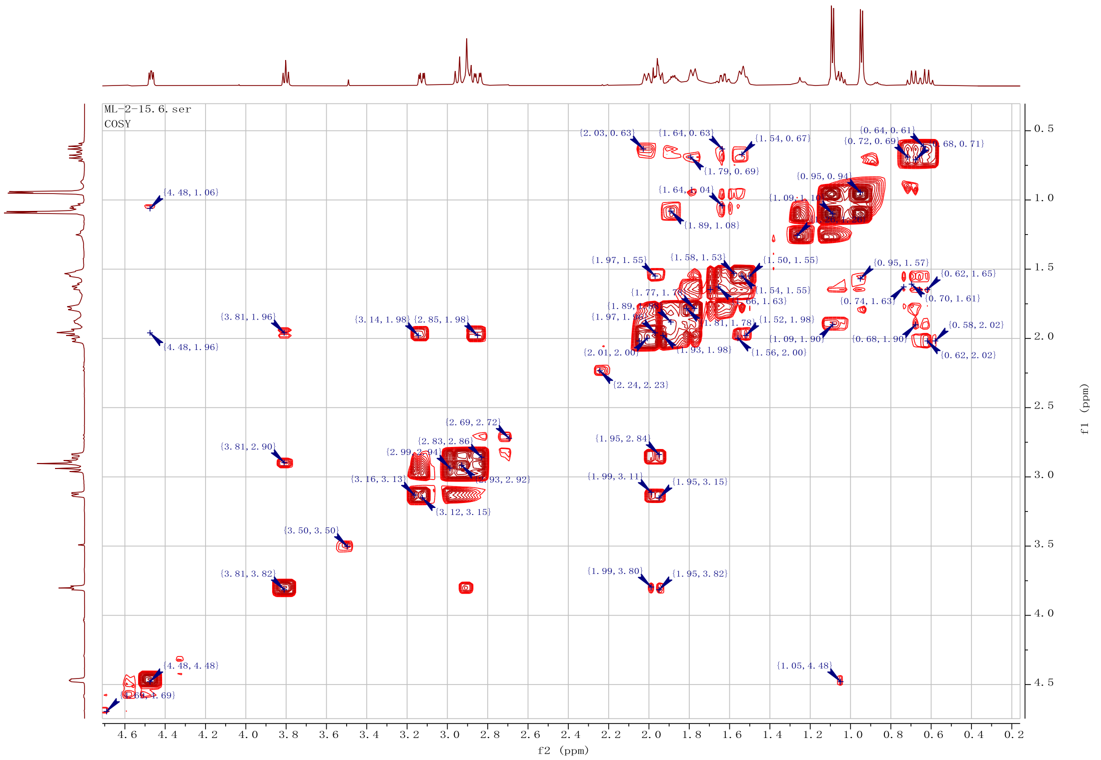

Enlarged figure

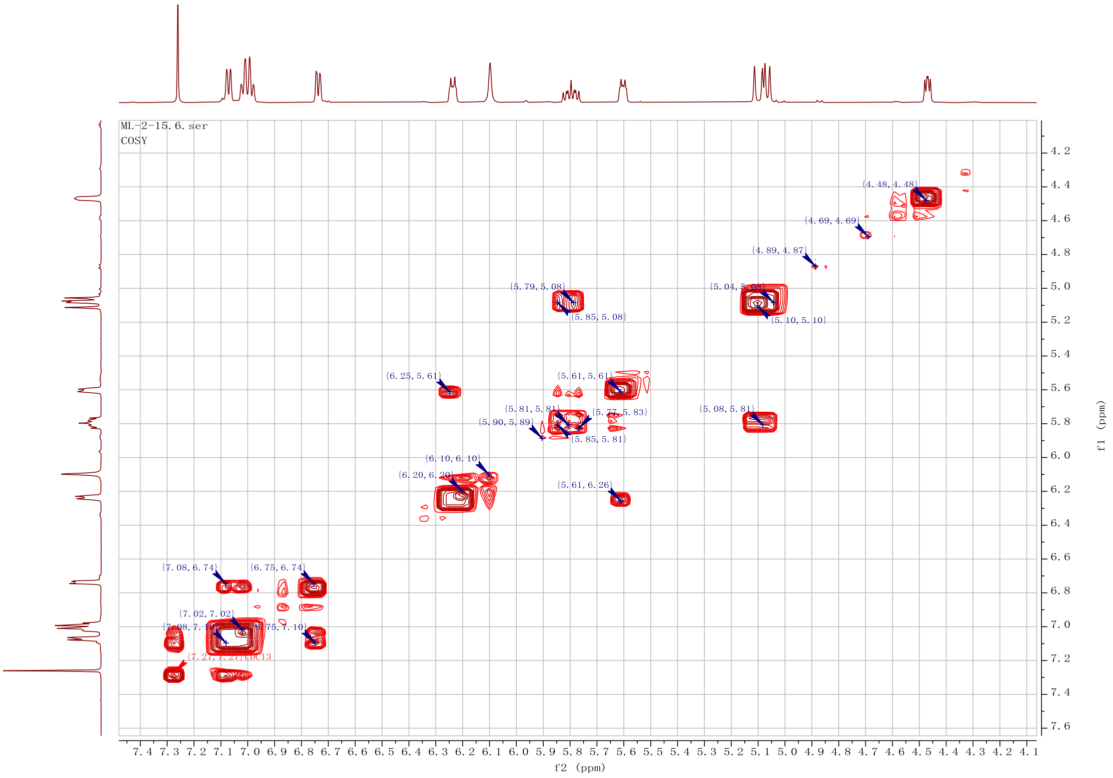

Figure S42 NOESY spectrum of xenoacremone H (**5**) in $CDCl_3$

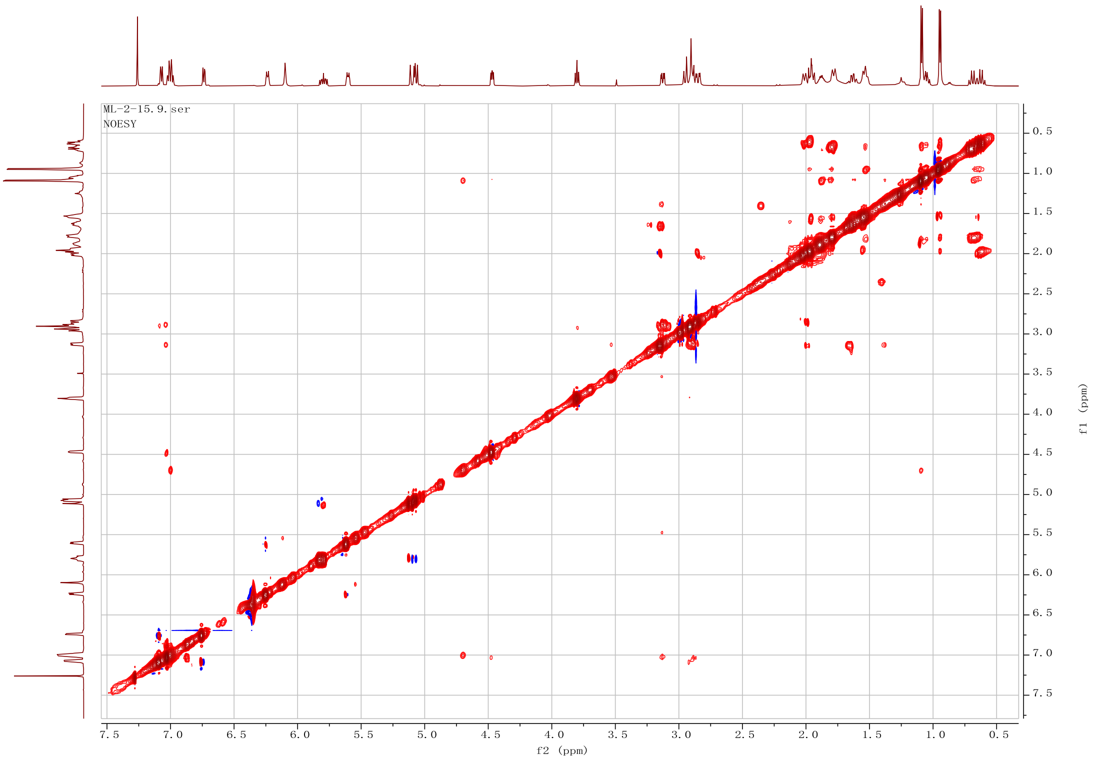

Enlarged figure

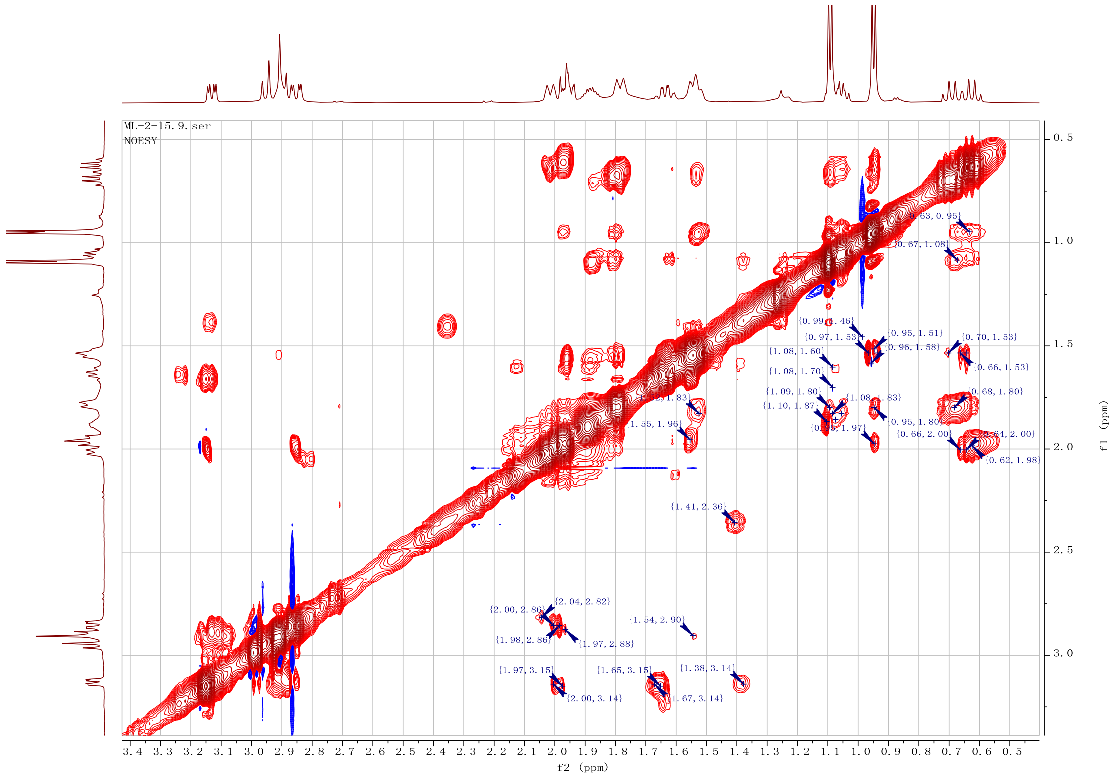

Enlarged figure

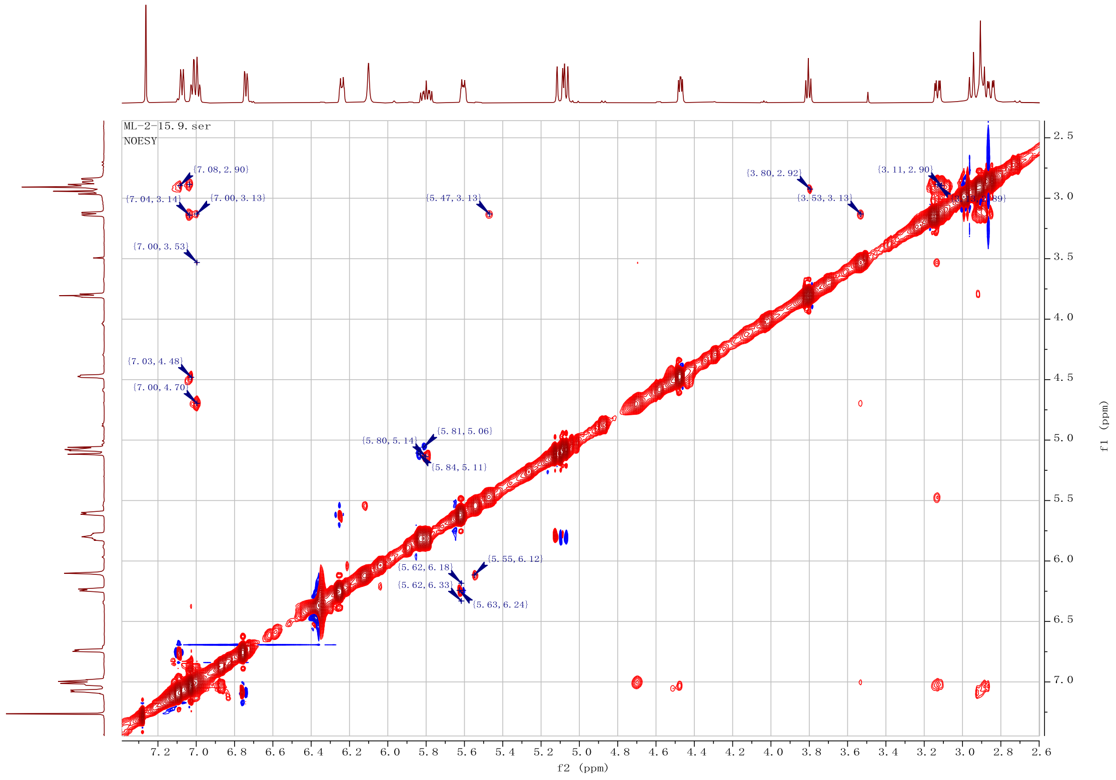

Figure S43 ECD spectrum of xenoacremone H (**5**) (in MeOH)

TDDFT theory, ωB97XD functional and TZVP level of theory, **5** has 2 conformations. Cam-B3LYP functional and TZVP level calculate 70 excited states, the solvent is methanol, σ=0.35eV, the result is shown in the figure.

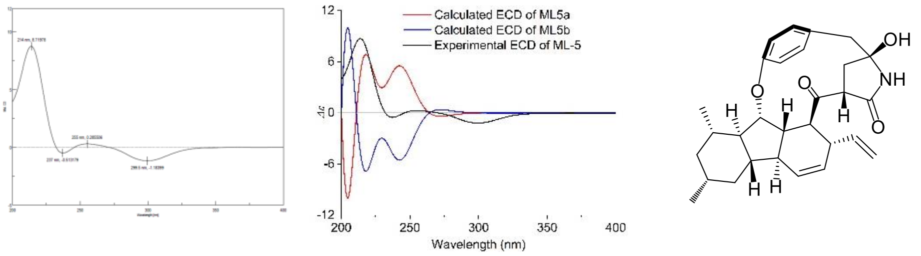

Figure S44 HRESI-MS spectrum of xenoacremone H (**5**)

Composition	i-FIT Confidence (%)	m/z RMS (PPM)	Intensity RMS (%)	Predicted m/z	m/z error (PPM)	m/z error (mDa)	DBE
C29H35 NO4	2.299482	4.885227	10.146764	462.2438 85	3.956426	1.824935	13.00 0000

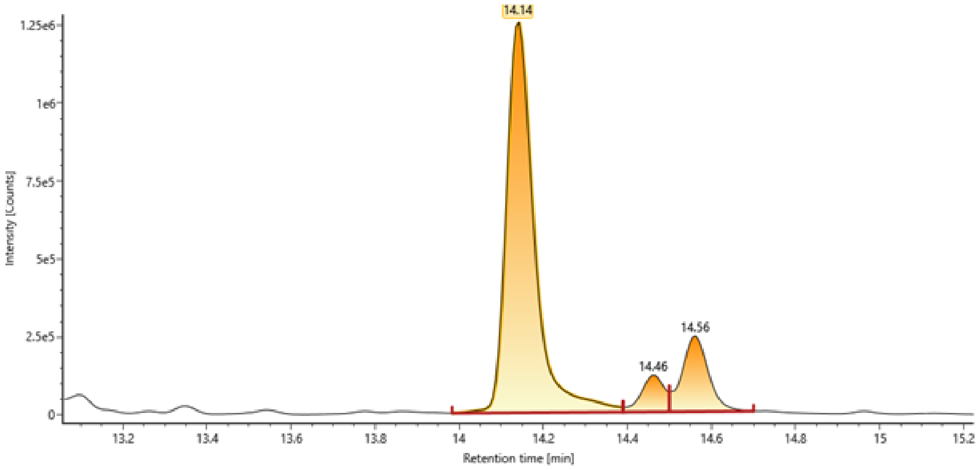

The conformers' data of compound 1：

1) Conformer -**1**C1

O	1.46993100	-2.12620900	0.31710900
O	-5.75808500	-0.70301900	-0.85144600
H	-5.76711800	-1.24594100	-1.65215800
O	-4.10394000	2.01195500	1.86274600
N	-4.55913100	-0.05486300	0.98495900
H	-5.27935300	-0.28759100	1.65765700
C	-0.63414700	-2.29555200	1.45310400
H	-0.14443900	-2.02759900	2.38005900
C	-1.86966000	-2.90573300	-0.95154500
H	-2.34919300	-3.14007700	-1.89501100
C	-2.02295300	-2.34637600	1.37359600
H	-2.61167800	-2.12304200	2.25477400
C	0.12628000	-2.46319900	0.30362600
C	0.93506900	0.30176300	0.22734000
H	0.70568400	-0.01948100	1.24107300
C	3.18221400	-0.49298900	-0.40367800
H	3.37820700	-0.03035800	-1.37912900
C	3.25698900	0.62686100	0.63880300
H	3.11982600	0.17167000	1.62746000
C	-2.89493500	1.15745200	-0.10467100
C	-1.52981300	0.83042700	0.53526900
C	-3.92511300	1.13503400	1.02461500
C	-0.39534700	0.65449200	-0.45306900
H	-0.69592600	-0.21602300	-1.03514300
C	1.70039000	-0.88570900	-0.43014200
H	1.36260200	-1.06484300	-1.45069200
C	2.02698100	2.26394700	-0.89252100
H	2.95708500	2.77079000	-1.12733200
C	-4.12204300	-2.29313800	-0.03994400
H	-4.72079700	-2.63864400	0.80519000
H	-4.46783700	-2.82512300	-0.92782400
C	4.24827500	-1.56674600	-0.20069000

H	4.06619000	-2.05606500	0.76197600
C	4.61343300	1.31019900	0.63221100
H	4.66026300	2.09287200	1.39606000
H	4.79396100	1.79216000	-0.33472600
C	-0.38098500	1.84648800	-1.46827600
H	-0.79342200	1.50067700	-2.41936800
C	5.71675500	0.26903500	0.87929900
H	5.55043100	-0.15575200	1.87715600
C	-2.65867800	-2.56982500	0.15463400
C	-2.75015800	2.54277400	-0.79127500
H	-3.24824500	3.29811700	-0.18204300
H	-3.25219700	2.51224000	-1.75729800
C	-0.48575200	-2.86034000	-0.88375800
H	0.11599800	-3.04682200	-1.76431600
C	1.97573800	1.43275400	0.35666900
H	1.73355900	2.07775700	1.20717700
C	4.22879000	-2.62674800	-1.30088500
H	4.38390800	-2.16670300	-2.28182500
H	3.27937200	-3.16406900	-1.32773200
H	5.02436300	-3.36151800	-1.14931300
C	7.10302600	0.90815700	0.85891200
H	7.31503700	1.34535800	-0.12186900
H	7.88372500	0.17254900	1.07126400
H	7.18289400	1.70520900	1.60328900
C	-3.41545500	0.04389700	-1.05456800
H	-3.94364500	0.50910900	-1.88374900
H	-2.62553600	-0.57284700	-1.46634800
C	0.99059700	2.40436800	-1.71645800
H	1.08720800	3.02611900	-2.60174700
C	5.62185700	-0.87630400	-0.13873200
H	6.39036400	-1.62370000	0.08234400
H	5.85339100	-0.47631100	-1.13450300
C	-1.30885300	2.99068400	-1.00124100
H	-1.29686700	3.77747900	-1.76130500

O	-0.84964500	3.53379000	0.24783700
H	0.09407400	3.73152400	0.15653700
C	-4.45154100	-0.79011800	-0.25379800
O	-1.39747700	0.74218100	1.73872000

No imaginary frequency.

Gibbs Free Energies (a.u.) = -1557.231210

2) Conformer -**1**C2

C	-5.54491500	-1.31662000	-0.31082100
C	-5.92270100	0.16438300	-0.13988300
C	-4.86159300	0.92851900	0.67224500
C	-3.49701600	0.71814900	0.04186100
C	-3.15310600	-0.77623100	0.00372800
C	-4.12914200	-1.56330700	-0.86358900
C	-2.23840100	1.29978500	0.68352800
C	-1.15294400	0.61596500	-0.16395800
C	-1.64297100	-0.85823900	-0.28550800
C	-2.05538700	2.77998200	0.69487800
C	-0.84672600	3.32279200	0.57277500
C	0.43946400	2.55348200	0.46176000
C	0.25552300	0.98543900	0.29215200
C	-7.31039100	0.30550900	0.48105500
C	-3.81064700	-3.05610200	-0.90793700
O	-1.10030200	-1.78293600	0.70309700
C	0.21064100	-2.17160500	0.53820500
C	0.71347000	-2.63268500	-0.67382800
C	2.08952900	-2.73922200	-0.84097700
C	2.96739000	-2.43664200	0.19895100
C	2.42377400	-2.20072700	1.46683100
C	1.05614000	-2.05829500	1.63845600
C	4.41662300	-2.15028800	-0.07193700
C	1.33303500	0.53932500	-0.66606500
C	4.68843400	-0.62592800	-0.14760200
C	3.82576500	0.16914500	-1.15574800

C	2.77337200	0.95773200	-0.35038300
N	4.35267900	0.02974600	1.10569200
O	1.07249000	0.07235000	-1.75923900
O	6.07937800	-0.51984600	-0.41322400
C	3.22987100	0.77187300	1.10549600
O	2.71674900	1.25761400	2.11047000
C	1.35350300	3.16489900	-0.61671400
C	2.74193700	2.49847900	-0.67862700
O	0.71654600	3.21025800	-1.90918000
H	-3.26578300	-1.14256900	1.03143100
H	-2.18043000	0.92071200	1.71387600
H	-3.54066900	1.09503400	-0.98842400
H	-1.27388300	1.01856400	-1.17499200
H	0.98942600	2.68984100	1.39509000
H	-6.28052400	-1.80275300	-0.95946500
H	-5.61808300	-1.81013000	0.66693300
H	-5.94917500	0.61686800	-1.13910200
H	-5.12235000	1.99065700	0.70790700
H	-4.85405600	0.56197800	1.70599700
H	-4.07805400	-1.16527400	-1.88471300
H	-1.42878900	-1.24997100	-1.27716200
H	-2.92774700	3.41484200	0.81766200
H	-0.73266500	4.40331800	0.58381500
H	0.46585300	0.54623700	1.26552700
H	-7.59995700	1.35636700	0.56863500
H	-8.06978000	-0.20157900	-0.12054800
H	-7.33339100	-0.13148700	1.48423500
H	-2.82105400	-3.24454600	-1.33043900
H	-3.83156200	-3.48761100	0.09718300
H	-4.54101100	-3.59401200	-1.51863600
H	0.05340600	-2.79474100	-1.51471900
H	2.48498000	-2.98333400	-1.81981500
H	3.07877700	-2.03862100	2.31305600
H	0.64404100	-1.76714600	2.59650000

H	5.06130200	-2.54777200	0.71551800
H	4.73036300	-2.58701500	-1.02056200
H	4.48133000	0.85856700	-1.68663100
H	3.36087500	-0.48043600	-1.89187100
H	4.75839000	-0.27921900	1.98052200
H	6.30442900	0.41553600	-0.52074100
H	1.50256700	4.21506500	-0.36722400
H	3.13225000	2.63414600	-1.68655700
H	3.41499600	3.01019000	0.00983400
H	0.52315400	2.31515000	-2.21740700

No imaginary frequency.

Gibbs Free Energies (a.u.) = -1557.230318

The conformers' data of compound 2:

1) Conformer-**2**C1

O	1.09047900	-1.85923100	1.10549500
O	-5.65725800	-0.83350700	-1.35095500
H	-5.77673600	0.08872700	-1.62515400
O	-3.11541100	1.28966800	1.80207600
N	-4.48240300	-0.06791100	0.56457100
H	-5.18509300	-0.17750100	1.28484400
C	-1.17567500	-1.99608300	1.87737600
H	-0.87853900	-1.55459300	2.82006000
C	-1.91649700	-3.07473200	-0.57393200
H	-2.20075900	-3.47545300	-1.53974800
C	-2.51931300	-2.19080600	1.57317800
H	-3.27031100	-1.90022500	2.29669800
C	-0.21355300	-2.27869800	0.91669900
C	1.12977300	0.60403000	0.77287600
H	1.22157800	0.46225600	1.85487200
C	2.94303000	-0.77669900	-0.18417300
H	2.97429200	-0.57348200	-1.26155100
C	3.49970400	0.48156300	0.50247700
H	3.60149700	0.25552300	1.57364700
C	-2.53399400	0.57275200	-0.49611000
O	-3.33648000	1.00303700	-1.62713300
C	-1.04505700	0.84101400	-0.64229100
C	-3.37319500	0.66881500	0.78209400
C	-0.25920100	1.26809700	0.57304400
H	-0.86920300	1.01911300	1.43525000
C	1.43770400	-0.80111700	0.17116100
H	0.86630900	-0.97270300	-0.73454800
C	2.29858600	2.64151300	-0.13910000
H	3.20303500	3.14116800	-0.47002700
C	-4.30235300	-2.41917100	-0.20353300
H	-5.07131800	-2.64083600	0.53824700
H	-4.49078100	-3.02592100	-1.08947300

C	3.82695500	-1.99767200	0.05728100
H	3.84246500	-2.20733400	1.13251700
C	4.86944000	0.84385200	-0.03413200
H	5.26174400	1.74050300	0.45439800
H	4.80377500	1.06052700	-1.10660400
C	-0.10713400	2.83594700	0.60741500
H	0.22535300	3.05735700	1.62431700
C	5.82531700	-0.33794300	0.19640300
H	5.91426100	-0.47551900	1.28119800
C	-2.90772900	-2.64622800	0.31168600
C	-1.92589400	4.43131400	1.23281500
H	-1.47927300	4.60875000	2.20639900
H	-2.83672500	4.97318700	1.00415800
C	-0.57506000	-2.90309700	-0.27509400
H	0.18711900	-3.17147000	-0.99454500
C	2.33633000	1.41465300	0.36137100
C	3.34330800	-3.24446700	-0.68166400
H	3.25130900	-3.04599400	-1.75399200
H	2.37403200	-3.58233500	-0.31650100
H	4.05361800	-4.06641600	-0.55637000
C	7.21647900	-0.04787400	-0.36134800
H	7.17793900	0.09791800	-1.44534900
H	7.90575500	-0.87248900	-0.16026600
H	7.63958900	0.85774800	0.08198000
C	-3.24371800	-0.39045400	-1.36101300
H	-2.73922300	-0.98555600	-2.10850200
C	1.00684000	3.37899100	-0.31282200
H	1.14418400	4.42719600	-0.04135600
O	0.63204700	3.42797600	-1.70920300
H	0.55729400	2.51444200	-2.02438300
C	5.25566400	-1.63626800	-0.39502200
H	5.92678500	-2.46645700	-0.15410300
H	5.25261600	-1.54658500	-1.48892900
C	-1.38777200	3.58286500	0.36584600

H	-1.86541000	3.43840000	-0.59923000
C	-4.46430600	-0.93746200	-0.60757300
O	-0.56043000	0.74126000	-1.75446200

No imaginary frequency.

Gibbs Free Energies (a.u.) = -1631.215883

2) Conformer -**2**C2

O	1.10220700	-1.94144000	0.94522800
O	-5.72484000	-0.68671200	-1.22424600
H	-5.85006300	0.25747000	-1.40585300
O	-3.06931000	1.09990300	2.04572700
N	-4.48589900	-0.10969900	0.71637000
H	-5.16398600	-0.29208500	1.44559300
C	-1.14709000	-2.14888800	1.74690700
H	-0.82621800	-1.80937200	2.72342900
C	-1.94891100	-2.96806800	-0.78435200
H	-2.25658400	-3.26810000	-1.77907200
C	-2.49781700	-2.31616900	1.45779500
H	-3.23058600	-2.10728900	2.22683000
C	-0.20888200	-2.32730300	0.73855900
C	1.14235900	0.53945200	0.83915000
H	1.23645200	0.29752700	1.90295600
C	2.96209400	-0.74664900	-0.22357400
H	3.00441200	-0.44107100	-1.27605900
C	3.51294900	0.44143500	0.58456900
H	3.59962700	0.11848900	1.63166200
C	-2.56370000	0.62013400	-0.33392200
O	-3.39456400	1.17255800	-1.38829700
C	-1.07456700	0.87082500	-0.50599200
C	-3.36265900	0.59128000	0.97375900
C	-0.24573400	1.22104700	0.70580900
H	-0.83558800	0.93777600	1.57167200
C	1.45344400	-0.80365500	0.11251800
H	0.88939200	-0.89206100	-0.80958900

C	2.31207800	2.63938400	0.08018700
H	3.21780700	3.16322500	-0.20640000
C	-4.32879400	-2.37326200	-0.28363100
H	-5.07410800	-2.67181000	0.45538500
H	-4.54114000	-2.89156200	-1.21907500
C	3.83962900	-1.98823400	-0.09244100
H	3.84091400	-2.30375700	0.95665900
C	4.89004800	0.84559400	0.09930500
H	5.27974100	1.69120300	0.67351700
H	4.83762800	1.16155500	-0.94894100
C	-0.08689600	2.78732300	0.79526900
H	0.29351100	2.94282500	1.81066700
C	5.83969200	-0.35654600	0.22922700
H	5.91407100	-0.59652200	1.29716800
C	-2.91831900	-2.64058600	0.16649100
C	-1.84568100	4.36735800	-0.16133000
H	-1.25006800	4.67336500	-1.01046000
H	-2.83177800	4.80857500	-0.06486500
C	-0.60049400	-2.82287300	-0.50328000
H	0.14350900	-3.01413400	-1.26538500
C	2.35055700	1.38407900	0.50499200
C	3.35876100	-3.15210100	-0.95799300
H	3.29231300	-2.84928400	-2.00758400
H	2.37752900	-3.51048500	-0.64768900
H	4.05677100	-3.99177500	-0.89863300
C	7.23874200	-0.02025900	-0.28083600
H	7.21466300	0.22655000	-1.34680900
H	7.92354500	-0.86243100	-0.14925800
H	7.65807400	0.83831500	0.25086000
C	-3.31131800	-0.24214100	-1.26979300
H	-2.83648200	-0.76049600	-2.08997000
C	1.00955900	3.36058000	-0.11394200
H	1.12506700	4.41232100	0.15366700
O	0.68337100	3.39024200	-1.52327400

H	0.57388000	2.47321400	-1.81824100
C	5.27469200	-1.59078500	-0.49024600
H	5.94107400	-2.44236000	-0.32165000
H	5.28559200	-1.39669000	-1.57046800
C	-1.41868800	3.48742500	0.73882400
H	-2.08506100	3.21521000	1.54975900
C	-4.50777600	-0.85966900	-0.53548900
O	-0.62514900	0.82284600	-1.63609300

No imaginary frequency.

Gibbs Free Energies (a.u.) = -1631.213339

The conformers' data of compound 3

1) Conformer -**3**C1

C	-1.67783800	-0.82663500	-0.02906600
C	-1.21588700	0.53105000	-0.61999400
C	-2.29353400	1.54588200	-0.19408300
C	-4.18457400	-1.71955000	0.12731700
C	-3.16379300	-0.59808400	0.33201600
C	-3.56289400	0.70687400	-0.38316200
C	-4.89775900	1.22307100	0.13358400
C	-0.91557200	3.17316700	-1.42270300
C	0.39320000	2.49485000	-1.05661600
C	0.23372900	1.01736100	-0.48056100
H	6.52063000	-0.59075300	-1.52999300
H	7.19356000	0.64705800	-0.44228800
H	5.70522600	0.98995000	-1.35580500
H	2.71315900	2.62312400	1.53377400
H	2.79985500	3.88422600	-1.29483300
H	2.68694900	4.77828600	0.32522800
H	-3.74208600	-2.78097000	1.97622900
H	-2.88091700	-3.41770400	0.56283400
H	-7.65683600	1.54702900	-0.02261100
H	0.84785700	0.38968000	-1.11915200
O	5.56284400	-0.27822600	0.31380100
H	-0.79569700	4.08025000	-2.01359900
H	-5.18899500	2.15103100	-0.37666100
C	6.26760000	0.21079500	-0.82592700
N	3.74020400	-1.11980800	1.39496500
O	2.78863400	1.78351700	2.02609000
H	-1.36308800	0.41741400	-1.70154100
H	-3.00446900	3.38909600	-1.25096800
C	3.29258000	0.63429700	-0.10341400
H	2.75425500	0.56362600	-1.04524300
C	1.89735600	-1.66304000	-2.28280600
C	0.50053000	-1.69677400	-2.30005100

C	2.25385500	4.06276200	-0.37042500
C	2.65116200	-0.53810500	1.93560900
C	2.36266100	0.75375100	1.12627600
C	0.52233600	-2.82142600	-0.16018900
H	3.92542400	1.52091100	-0.14805200
H	4.10405200	-1.98772500	1.77524600
H	2.44779900	-3.26516400	0.65617100
H	-3.66893200	0.49807300	-1.45969600
H	4.47398400	-1.35832200	-1.90966100
H	-1.10713100	-1.11828700	0.85455400
H	4.67805900	-2.57298400	-0.63910600
H	2.42710800	-1.18508200	-3.10307300
H	-0.02083100	-3.25814700	0.67242400
H	-0.05735500	-1.24716900	-3.11554600
H	0.57060900	3.65482000	0.82124100
H	-4.22526600	-1.96003200	-0.94461800
H	-4.60707400	-3.75669300	0.77664100
H	-5.55523700	-1.06284200	1.65894100
H	-8.11960700	-0.13940500	0.27073400
H	-7.29856000	0.78773500	1.53833400
H	-6.33050600	-1.94858300	0.34961600
H	-2.19163500	1.77147000	0.87757200
H	1.00802600	2.42167600	-1.96045700
C	4.19132500	-0.62135400	0.11601900
C	1.09815400	3.44507200	-0.10933900
H	-3.15959500	-0.36434500	1.40800700
H	-4.81446800	1.45831600	1.20556500
H	-6.07539900	-0.01397700	-1.16027700
O	2.04269000	-0.92150200	2.92889700
C	0.82074800	0.92414400	0.91214900
O	0.13363200	1.02415800	1.91725200
C	1.91242000	-2.80663600	-0.16843900
C	4.05651800	-1.73278300	-0.97136600
C	2.61791500	-2.13034900	-1.17654200

C	-0.17954700	-2.17556400	-1.18124800
O	-1.52561300	-1.89843500	-1.01404500
C	-3.83018100	-2.99200500	0.90226800
C	-7.34095700	0.61124400	0.45533300
C	-2.12582400	2.79805000	-0.99612300
C	-5.98241400	0.14853100	-0.07518600
C	-5.56661200	-1.18910000	0.56489400

No imaginary frequency.

Gibbs Free Energies (a.u.) = -1595.926337

2) Conformer -**3**C2

C	-1.67941500	-0.79426300	0.27525100
C	-1.21449800	0.39887300	-0.61582300
C	-2.41467800	1.38461700	-0.58225700
C	-4.13151000	-1.79349400	0.51431700
C	-3.19987000	-0.58119900	0.44757600
C	-3.59509900	0.41814400	-0.65301100
C	-5.00659900	0.94371300	-0.43343400
C	-1.01578000	3.00409800	-1.73541400
C	0.09208400	2.65931000	-0.75382100
C	0.19146000	1.08372300	-0.54845100
H	7.09538100	0.48230200	-1.69187100
H	5.45178100	0.39089900	-2.36774100
H	6.40179000	-1.10095100	-2.11582200
H	2.41222200	2.75239300	1.47810900
H	0.28313800	5.05254100	1.81027600
H	1.38948700	4.86434200	0.33620400
H	-3.81138900	-2.21849600	2.62588000
H	-2.78063500	-3.16976300	1.54065500
H	-7.74575500	0.96357600	-0.91947800
H	0.75166000	0.73348700	-1.41587800
O	5.72010700	-0.16936600	-0.36008300
H	-0.80369200	3.77647600	-2.47142900
H	-5.05246300	1.49359500	0.51898900

C	6.17516600	-0.10759700	-1.71060600
N	4.19115600	-0.57060100	1.27600800
O	2.84908700	2.41514900	0.67414900
H	-1.25467500	-0.01322400	-1.62962800
H	-3.03797200	2.69866200	-2.30295400
C	3.34292900	0.47649700	-0.65010800
H	2.67273100	0.07203000	-1.40381400
C	2.05120400	-2.63810500	0.54735400
C	0.66314800	-2.60579700	0.63535700
C	0.53982400	4.49885600	0.90991000
C	3.31813700	0.32762300	1.77492600
C	2.60392100	1.00783300	0.59054400
C	0.52229600	-2.23168000	-1.75308400
H	3.88921500	1.31172000	-1.09202200
H	4.80255000	-1.10813400	1.88242000
H	2.40103600	-2.11023400	-2.78724700
H	-3.56405200	-0.09697600	-1.62615400
H	4.50690900	-2.06011200	-1.75072200
H	-1.17535700	-0.83024000	1.23888500
H	4.78038200	-2.70088900	-0.12478400
H	2.63885100	-2.79410700	1.44649200
H	-0.08455000	-2.06005100	-2.63661700
H	0.16650000	-2.72867900	1.59283000
H	-1.00383700	3.10524600	1.13216900
H	-4.05295200	-2.34353100	-0.43372400
H	-4.49640400	-3.57533700	1.71542800
H	-5.68491100	-0.82348400	1.65848000
H	-8.14101900	-0.59043600	-0.16252900
H	-7.51712300	0.74293200	0.82416400
H	-6.27195400	-2.12581000	0.62904200
H	-2.46069100	1.86670600	0.40440700
H	1.05062400	2.99079100	-1.15843000
C	4.36782300	-0.59140900	-0.15919200
C	-0.16249400	3.43124700	0.52491100

H	-3.31005500	-0.04158000	1.40117800
H	-5.29424000	1.64919900	-1.22454200
H	-5.96396600	-0.72339200	-1.38027200
O	3.15102400	0.59579300	2.96306200
C	1.04418000	0.82004100	0.67896600
O	0.54515500	0.61116400	1.77388200
C	1.91360600	-2.26585600	-1.82802900
C	4.15233100	-2.02604000	-0.71772400
C	2.69548300	-2.39315700	-0.67316300
C	-0.09298400	-2.32514100	-0.50591300
O	-1.44365900	-2.06470000	-0.40065600
C	-3.78177900	-2.74433800	1.66234600
C	-7.43109800	0.24604500	-0.15132200
C	-2.22095500	2.43310600	-1.63451300
C	-5.99884300	-0.23422400	-0.39434400
C	-5.57772100	-1.27502200	0.65960900

No imaginary frequency.

Gibbs Free Energies (a.u.) = -1595.924788

3) Conformer -**3**C3

C	-1.61657300	-0.83799800	0.29420100
C	-1.15548400	0.35702400	-0.59482300
C	-2.37579400	1.31728200	-0.61766400
C	-4.05521500	-1.86568600	0.52620100
C	-3.14023600	-0.64184000	0.44411200
C	-3.53607800	0.32803500	-0.68294100
C	-4.95915400	0.83457600	-0.49346700
C	-0.97781100	2.94500700	-1.74891600
C	0.05636700	2.66883300	-0.68022100
C	0.22291100	1.08939100	-0.48995500
H	7.13716800	0.72340100	-1.67693400
H	5.48915900	0.65608800	-2.34530000
H	6.44847100	-0.84119100	-2.17265900
H	2.64199200	2.65913900	1.78324300

H	-2.00828500	4.45699800	0.12246300
H	-1.31425700	4.76362300	1.81255400
H	-3.74688400	-2.24620700	2.64822700
H	-2.69325600	-3.20206000	1.58924900
H	-8.07402400	-0.73834900	-0.21950300
H	0.80393900	0.78125800	-1.35909200
O	5.77482400	0.00070000	-0.36906800
H	-0.73859200	3.67938400	-2.51478500
H	-5.02492800	1.40675200	0.44458500
C	6.21992900	0.13018900	-1.71838000
N	4.26656000	-0.49874100	1.25766600
O	2.80454100	2.47311700	0.83887600
H	-1.15014200	-0.06953000	-1.60278800
H	-2.93829500	2.51244900	-2.44710400
C	3.38555600	0.62442600	-0.61269400
H	2.73179200	0.25094100	-1.39624700
C	2.17406900	-2.58221300	0.48352900
C	0.78729600	-2.60607700	0.59701700
C	-1.23384700	4.25121300	0.85660200
C	3.37484600	0.35177500	1.79847800
C	2.62979000	1.06081200	0.65416100
C	0.58896600	-2.15844500	-1.77399300
H	3.91805100	1.49615700	-0.99720600
H	4.89598100	-1.04710300	1.83532500
H	2.44166100	-1.92367500	-2.83546700
H	-3.48274700	-0.20874100	-1.64355900
H	4.56213100	-1.83872200	-1.84041200
H	-1.12280800	-0.87140500	1.26315100
H	4.89059500	-2.54733800	-0.25358000
H	2.78325500	-2.74972300	1.36600300
H	-0.03972800	-1.98466800	-2.64157800
H	0.31323000	-2.77828200	1.55839600
H	0.51378100	3.26543000	1.39864800
H	-3.95924000	-2.43254500	-0.41029900

H	-4.40421900	-3.62991300	1.75781700
H	-5.63366400	-0.89250100	1.63296000
H	-7.47999700	0.62683400	0.74180500
H	-7.69256600	0.80293100	-1.00890300
H	-6.19164800	-2.22712500	0.62880900
H	-2.43740200	1.83826100	0.34730000
H	1.03486800	3.02378500	-1.02197300
C	4.43132200	-0.45033400	-0.17922300
C	-0.22710500	3.41036900	0.61714800
H	-3.26714000	-0.08312700	1.38472800
H	-5.24781700	1.51654200	-1.30467900
H	-5.88182000	-0.86883500	-1.41015600
O	3.20963100	0.57790500	2.99607300
C	1.07820900	0.81253200	0.73292400
O	0.58783000	0.54401900	1.81861900
C	1.97871900	-2.13029600	-1.87374500
C	4.23243600	-1.86091400	-0.79887400
C	2.78632800	-2.26830200	-0.73807200
C	-0.00033200	-2.31812300	-0.52041100
O	-1.35716300	-2.10755100	-0.38098000
C	-3.70168700	-2.78933800	1.69496100
C	-7.37618900	0.10833100	-0.22062300
C	-2.16332300	2.32666000	-1.70552000
C	-5.93456100	-0.35661200	-0.43677000
C	-5.50999600	-1.36582200	0.64614400

No imaginary frequency.

Gibbs Free Energies (a.u.) = -1595.923060
